# Twenty Novel Disease Group-Specific and 12 New Shared Macrophage Pathways in Eight Groups of 34 Diseases Including 24 Inflammatory Organ Diseases and 10 Types of Tumors

**DOI:** 10.3389/fimmu.2019.02612

**Published:** 2019-11-14

**Authors:** Bin Lai, Jiwei Wang, Alexander Fagenson, Yu Sun, Jason Saredy, Yifan Lu, Gayani Nanayakkara, William Y. Yang, Daohai Yu, Ying Shao, Charles Drummer, Candice Johnson, Fatma Saaoud, Ruijing Zhang, Qian Yang, Keman Xu, Kevin Mastascusa, Ramon Cueto, Hangfei Fu, Susu Wu, Lizhe Sun, Peiqian Zhu, Xuebin Qin, Jun Yu, Daping Fan, Ying H. Shen, Jianxin Sun, Thomas Rogers, Eric T. Choi, Hong Wang, Xiaofeng Yang

**Affiliations:** ^1^Centers for Inflammation, Translational and Clinical Lung Research, Lewis Katz School of Medicine at Temple University, Philadelphia, PA, United States; ^2^Department of Gastrointestinal Surgery, The Second Affiliated Hospital of Nanchang University, Nanchang, China; ^3^Department of Ultrasound, The Second Affiliated Hospital of Nanchang University, Nanchang, China; ^4^Division of Abdominal Organ Transplantation, Department of Surgery, Lewis Katz School of Medicine at Temple University, Philadelphia, PA, United States; ^5^Metabolic Disease Research, Cardiovascular Research, & Thrombosis Research, Departments of Pharmacology, Microbiology and Immunology, Lewis Katz School of Medicine at Temple University, Philadelphia, PA, United States; ^6^Department of Clinical Sciences, Lewis Katz School of Medicine at Temple University, Philadelphia, PA, United States; ^7^Division of Vascular and Endovascular Surgery, Department of Surgery, Lewis Katz School of Medicine at Temple University, Philadelphia, PA, United States; ^8^Tulane National Primate Research Center, School of Medicine, Tulane University, Covington, LA, United States; ^9^Department of Cell Biology and Anatomy, University of South Carolina School of Medicine, Columbia, SC, United States; ^10^Cardiothoracic Surgery Research Laboratory, Texas Heart Institute, Houston, TX, United States; ^11^Department of Surgery, Baylor College of Medicine, Houston, TX, United States; ^12^Center for Translational Medicine, Department of Medicine, Sidney Kimmel Medical College, Thomas Jefferson University, Philadelphia, PA, United States

**Keywords:** macrophages, disease-specific and shared pathways, immune checkpoint receptors, trained immunity, immunometabolism pathways

## Abstract

The mechanisms underlying pathophysiological regulation of tissue macrophage (Mφ) subsets remain poorly understood. From the expression of 207 Mφ genes comprising 31 markers for 10 subsets, 45 transcription factors (TFs), 56 immunometabolism enzymes, 23 trained immunity (innate immune memory) enzymes, and 52 other genes in microarray data, we made the following findings. (1) When 34 inflammation diseases and tumor types were grouped into eight categories, there was differential expression of the 31 Mφ markers and 45 Mφ TFs, highlighted by 12 shared and 20 group-specific disease pathways. (2) Mφ in lung, liver, spleen, and intestine (LLSI-Mφ) express higher M1 Mφ markers than lean adipose tissue Mφ (ATMφ) physiologically. (3) Pro-adipogenic TFs C/EBPα and PPARγ and proinflammatory adipokine leptin upregulate the expression of M1 Mφ markers. (4) Among 10 immune checkpoint receptors (ICRs), LLSI-Mφ and bone marrow (BM) Mφ express higher levels of CD274 (PDL-1) than ATMφ, presumably to counteract the M1 dominant status via its reverse signaling behavior. (5) Among 24 intercellular communication exosome mediators, LLSI- and BM- Mφ prefer to use RAB27A and STX3 than RAB31 and YKT6, suggesting new inflammatory exosome mediators for propagating inflammation. (6) Mφ in peritoneal tissue and LLSI-Mφ upregulate higher levels of immunometabolism enzymes than does ATMφ. (7) Mφ from peritoneum and LLSI-Mφ upregulate more trained immunity enzyme genes than does ATMφ. Our results suggest that multiple new mechanisms including the cell surface, intracellular immunometabolism, trained immunity, and TFs may be responsible for disease group-specific and shared pathways. Our findings have provided novel insights on the pathophysiological regulation of tissue Mφ, the disease group-specific and shared pathways of Mφ, and novel therapeutic targets for cancers and inflammations.

## Introduction

As we reported previously (1–5), monocytes and macrophages (Mφ) play significant roles in driving cardiovascular inflammations induced by various metabolic cardiovascular disease-related danger-associated molecular patterns (DAMPs) such as hyperlipidemia, hyperglycemia, hyperhomocysteinemia, and chronic kidney disease. Also, monocyte and Mφ differentiation during various metabolic cardiovascular diseases has been characterized (5). In recent years, a complicated relationship between the bone marrow, monocytes/Mφ, and the development of atherosclerotic plaques has begun to be revealed (6). The roles of Mφ in modulating foam cell formation (7) and inflammation resolution (8) have also been reported. Moreover, several additional developments have been made, including in tissue Mφ characterization, Mφ polarization, subset characterization (9), clonal production, and trained immunity (trained immunity) (10). A recent success on the CANTOS trials with anti-interleukin-1β (IL-1β) monoclonal antibody Canakinumab (11) further emphasized the significant roles of inflammatory cytokines in the pathogenesis of metabolic cardiovascular diseases, in which monocytes and Mφs secrete cytokines in large numbers and amounts in response to the stimulation of DAMPs or conditional DAMPs that we had reported (12). However, the molecular mechanisms underlying several vital aspects of Mφ remain poorly determined: (1) the expression of Mφ markers and Mφ transcription factors, (2) pathways in the regulating roles of Mφ in various diseases, and (3) the differentiation and transdifferentiation of tissue Mφ subsets.

Macrophages play significant roles in the pathogenesis of various diseases including cardiovascular (13), metabolic (14), infectious (15), respiratory (16), digestive (17), autoimmune (18), and many types of cancers (19, 20). However, three important questions remain: whether Mφ use the same pathways and play the same roles or whether they use disease-specific pathways and play disease-specific roles in addition to the shared roles and pathways; whether 10 Mφ subset markers and newly identified 27 TFs (21) and other 18 Mφ subset TFs are differentially expressed in tissues; and whether all these newly reported proinflammatory features of Mφ are differentially expressed in various tissues. Addressing these issues will improve our understanding of the disease-specific and shared roles and pathways of Mφ in the pathogenesis of various diseases and cancers and will lead to the identification of novel therapeutic targets specific to those diseases and cancers.

Determining novel mechanisms underlying macrophage disease-specific and shared pathways first requires an understanding of how macrophages respond to environmental and tissue functional cues from several aspects such as cell surface receptor signaling, cell-cell interaction receptor signaling, cell-cell communication signaling, intracellular immunometabolic pathways, and transcription factors. Macrophages are present in almost all tissues of the body, displaying distinct location-specific phenotypes and gene expression profiles (22). In addition to central roles in innate immunity and as modifiers of the adaptive immune responses, tissue Mφ play supportive functions to the tissues they reside in Hoeksema and Glass (13).

Several cell-surface-specific mechanisms could promote macrophage heterogeneity. First, by varying stimuli such as different cytokines or DAMPs to act on Mφ cell surface receptors, Mφ can be “polarized” into as many as total 10 macrophage subsets including the typical proinflammatory M1 Mφ and anti-inflammatory M2 Mφ (9). Also, as many as 28 T cell co-stimulation receptors and co-inhibition/immune checkpoint receptors as cell-cell contact signaling receptors may serve as a second cell surface mechanism to shape the antigen-presenting functions of Mφ (23). Finally, recent reports showed that exosomes are local and distal cell-cell communication vehicles (24), which may serve as the third cell-surface mechanism.

We also reported that exosomes secreted from immune cells such as macrophages might propagate inflammation from the first inflamed cells to the secondary inflammatory cells (25); exosomes regulate inflammation and immune responses via intercellular exosome communications (26). In addition to cell surface mechanisms, the M1 Mφ proinflammatory metabolic pathways and M2 metabolic pathways have been identified (27) as the intracellular mechanisms. Another development is the recognition of innate immune memory (trained immunity) pathways such as increased glycolysis pathway, enhanced acetyl-CoA (activated acetate, cellular acetyl donor) generation, and increased expression of mevalonate pathway enzymes (28, 29), which were “zoomed in” to through extensive metabolic remodeling of 2,722 experimentally elucidated pathways (https://metacyc.org). Recent reports also identified numerous transcription factors involved in Mφ differentiation and Mφ subset polarization.

**Figure 1:**
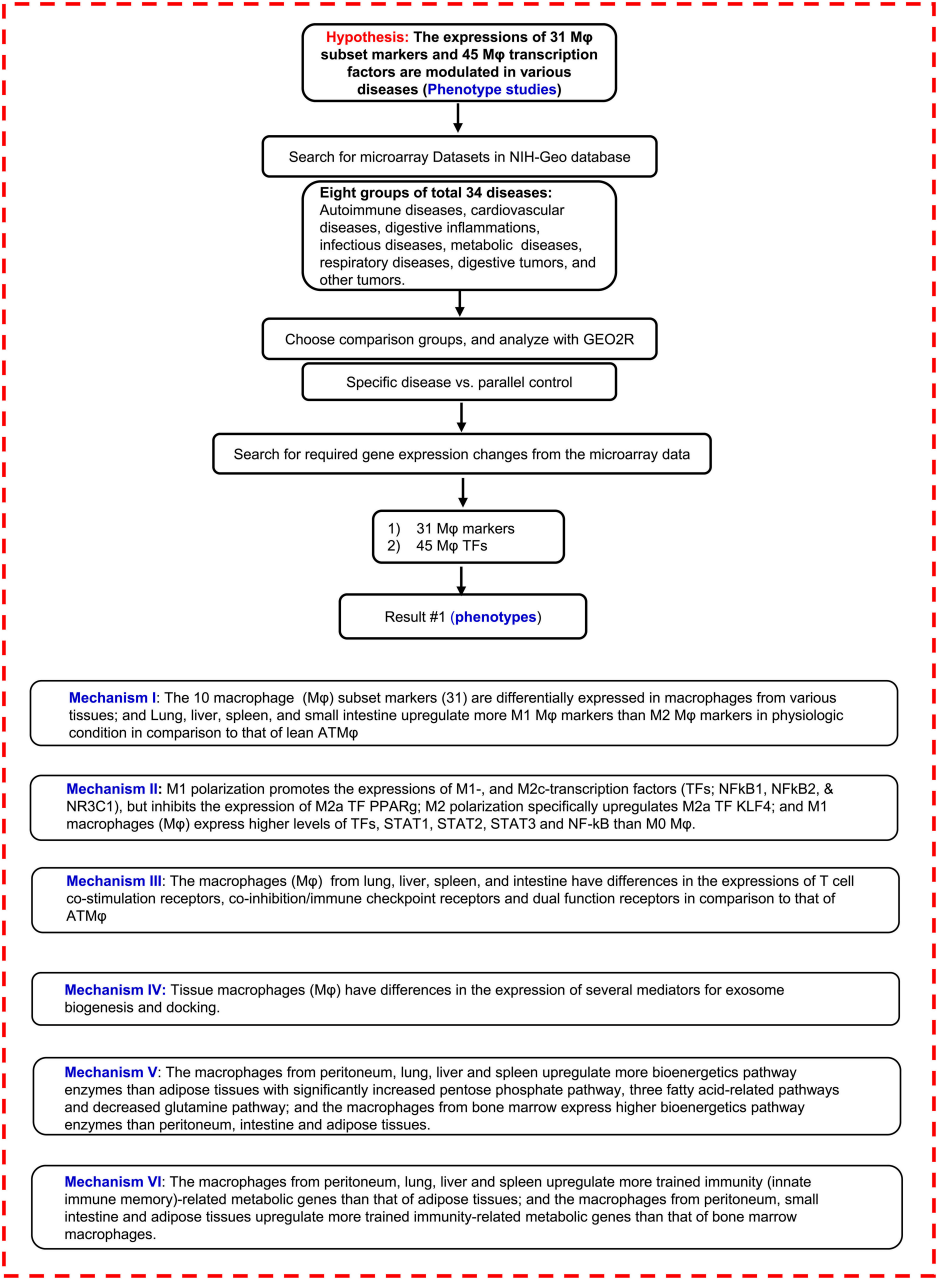
Flow chart of database mining strategy and two parts of data organization. (I) database mining strategy was used to analyze the macrophage (Mφ) marker tissue expression profiles in physiological conditions. (II) Mφ marker and Mφ transcription factor expression changes were analyzed on experimental data from the microarray datasets in different diseases. 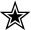 NIH-Geo website: www.ncbi.nlm.nih.gov/geo/; GEO2R website: www.ncbi.nlm.nih.gov/geo/geo2r/. Mφ, macrophages; TFs, transcription factors; T-reg, CD4+ regulatory **T** cell; KO, knock out; WT, wild type; ATMφ, adipose tissue macrophage; M1, type I macrophage; M2, type 2 macrophage.

Regardless of significant progress in the field, several important questions remain. The first is whether, under various disease conditions, Mφ uses both disease-specific signaling pathways and shared pathways. To determine the molecular mechanisms underlying disease-specific and shared pathways of macrophages, we examined macrophage features from tissue-specific differential expression of Mφ cell surface markers, transcription factors, Mφ cell-cell contact signaling receptors (T cell co-stimulation receptors and co-inhibition/immune checkpoint receptors), Mφ cell-cell communication vesicle–exosome biogenesis and docking machinery, and Mφ intracellular metabolism pathways such as bioenergy metabolism pathways and trained immunity (innate immune memory) pathways. We then narrowed in on the following questions: whether tissues have differential expression of Mφ subset markers and transcription factors, whether tissue Mφ have different inflammatory and trained immunity (innate immune memory) potentials, and whether tissue Mφ have different bioenergy metabolism pathways and trained immunity pathways. To address these issues, we determined the expression of 207 Mφ genes in several tissues such as lung, liver, intestine, spleen, and bone marrow-derived, including 10 subset markers, 45 transcription factors (TFs), and 127 other regulatory genes by analyzing the microarray experimental data sets that other investigators deposited in the NIH-NCBI GEO DataSets database, as shown in Figure 1. Of note, we pioneered this type of novel experimental data mining analysis in 2004 (30), which has allowed us to generate original findings and novel hypotheses for our experimental projects. The significant differences between our experimental database mining approaches and traditional literature reviews are detailed in Table 1. Based on the expression changes of 31 ten-Mφ-subset markers and 45 TFs in eight groups of a total of 34 diseases, including 10 types of cancers, we have identified 20 novel disease group-specific and 12 new shared macrophage pathways. In addition, we also found new signaling and metabolic pathways underlying tissue Mφ subset regulation in pathophysiological conditions as novel mechanisms for Mφ heterogeneity, which serve as novel therapeutic targets specific to cancers and inflammations.

**Table 1:** A novel research publication type utilizing big-omics experimental database mining analyses leads to original new findings and generates new hypotheses.

**Category**	**Big-omics database mining**	**Traditional literature review**
Analysis of experimental data (NIH Geo DataSets with microarray experimental data, etc.)	Yes	No
Original new findings	Yes	No
Association research (gene co-expression patterns at the same pathology or stimuli)	Yes	No
Causative research (upstream regulator gene-deficient microarrays, …)	Yes	No
Panoramic view at multiple mechanisms and pathways	Yes	Yes
Improvement of our understanding	Yes	Yes
Searchable database requirements and tools	Yes	No
New publication types after–omics and high throughput experimental data generation	Yes	No
Different focuses from original papers	Yes	No
Use of Ingenuity Pathway Analysis (IPA) to analyze experimental data	Yes	No
Bioinformatic prediction	No	No
Future experimental verification	Yes	Yes
Face the low-throughput problems in verifying high-throughput–omics data (also see Yao et al. Nature Immunology, PMID: 31209400)	Yes	No
Summary of previous reports	No	Yes
Example for our database mining paper on IL-35 (highly cited by 173 papers)		PMID: 22438968
Example for traditional literature review: a Nature Immunology review that cited our database mining paper on IL-35		PMID: 22990890
Our experimental papers verifying the findings originated from our database mining paper on IL-35		PMIDs: 26085094; 29371247
Use of multiple NIH databases including PubMed database (https://www.ncbi.nlm.nih.gov/books/NBK143764/)	Yes	No PubMed database only

*Comparisons were made regarding various aspect between this study, with a big-omics experimental database mining approach, and traditional literature reviews*.

## Results

### Expression of 31 Mφ Markers and 45 Mφ TFs Is Modulated in Eight Groups of a Total of 34 Diseases, Including 24 Inflammatory Organ Diseases and 10 Types of Cancers; and Both Shared and Disease-Specific Pathways for Each Group of Disease/Tumor Have Been Identified

Mφ play a key role in the pathogenesis of various diseases. However, two critical questions remain: whether Mφ use the same pathways and play the same roles or whether they use disease-specific pathways and play disease-specific roles in addition to the shared roles and pathways. To improve our understanding of the roles of Mφ in various diseases, we examined the expression of 31 Mφ subset markers and 45 Mφ transcription factors (Table 2) in eight groupings of a total of 34 diseases, including four types of autoimmune diseases, four types of cardiovascular diseases, four types of digestive diseases, four types of infectious diseases, four types of metabolic diseases, four types of respiratory diseases, five types of digestive cancers, and five types of other cancers. As shown in Table 3A, some Mφ markers were upregulated in more than 30% of the 34 diseases, including three M1 markers, CXCL11, CXCL10, and CXCL9, 2 M2 markers, CCL18 and IL1RN, and one M4 marker, MMP7, suggesting that these markers may play significant roles in the pathogenesis of the diseases. In addition, the diseases with Mφ markers upregulated in more than 30% of the 34 diseases were of eight types, including #5 myocardial infarction, #6 coronary artery disease, #10 gastritis, #11 Crohn's ileitis, #12 Crohn's colitis, #29 esophageal cancer, #32 ovarian carcinoma, and #34 renal carcinoma, suggesting that these diseases may have significant Mφ marker activities with the pathogenic processes. Moreover, as shown in Table 3B, some Mφ transcription factors (TFs) were upregulated in more than 30% of the 34 diseases, including M1 TF STAT1 and three other Mφ TFs such as HMGA1, E2F3, and NME1, suggesting that these TFs play significant roles in the pathogenesis of the diseases. Furthermore, the diseases having Mφ TFs upregulated in more than 30% among the 34 diseases were of six types, including #6 coronary artery disease, #12 Crohn's colitis, #28 hepatocellular cancer, #29 esophageal cancer, #32 ovarian carcinoma, and #33 lung cancer, suggesting that these diseases have significant Mφ TF activities with the pathogenic processes.

**Table 2:** A total of 207 macrophage (Mφ)-related regulator genes in seven representative groups were studied in this paper, including 31 Mφ subset marker genes, 18 Mφ subset transcription factor genes (TF), 27 Mφ general transcription factor genes, 28 T cell co-stimulation and co-inhibition receptor genes, 56 bioenergetics pathway enzyme genes, 23 trained immunity (innate immune memory) pathway genes, and 24 exosome biogenesis/docking mediator genes.

**Category**	**Type**	**Gene list**	**Number**	**Total number**	**PMID**	**Note**
Mφ markers (cell surface)	M1	IL1B, TNF, IL6, CXCL11, CXCL10, CXCL9, IL23A, IL12A, IL12B, ARG2	10	43–12 = 31	24998279	Detailed information see Figure S1
	M2a	MRC1, CD163, STAB1, CCL18, CD200R1, F13A1, IL1RN, ARG1, PDE4DIP, Chil4, Chil3, Retnla	12			
	M2b	IL10, IL12B, IL12A	3			
	M2c	MRC1, ARG1	2			
	M2d	TNF, IL12A, IL12B	3			
	M4	MMP7, MRC1, S100A8	3			
	Mox	HMOX1, NFE2L2, TXNRD1, SRXN1	4			
	M(hb)	CD163, MRC1	2			
	Mhem	CD163	2			
	HA-mac	CD163, HLA-DRB1, HLA-DRA	3			
Mφ TFs	M1	HIF1A, RELA, IRF3, STAT1, STAT2	5	19–1 = 18	25228902 25506346 28228760 23640482 25505468 26954942 26972048 25755062 25367649	Detailed information see Figure S2
(41⋆) (nuclear proteins)	M2a	PPARD, PPARG, KLF4, AKT1	4			
	M2b	MAPK1, STAT3	2			
	M2c	NFKB1, NFKB2, NR3C1, NFE2	4			
	M2d	N/A	0			
	M4	N/A	0			
	Mox	NR1H3	1			
	M(hb)	ATF1	1			
	Mhem	NR1H3, NR1H2	2			
	HA-mac	N/A	0			
	General Mφ TFs	CREB1, HMGA1, SMAD4, ZNF148, HBP1, CKLF, ZNF281, FOXO3, HEY1, ETS2, HIF1A, STAT4, MELTF, BATF3, NFE2, NFKB1, RIT1, HIVEP1, JUNB, NFX1, FOXN3, STAT3, PWWP3A, MXD4, E2F3, CEBPD, NME1	27	27	24530056	
Co-stimulation and co-inhibition receptors (cell-cell interaction receptors)	Co-stimulation receptors	ICOSLG, CD70, TNFSF14, CD40, TNFSF9, TNFSF4, TNFSF15, TNFSF18, TNFSF8, TIMD4, SLAMF1, CD48, SEMA4A, CD58	14	28	23470321 27192563	Detailed information see Figure S3
	Co-inhibition receptors	LGALS9, NECTIN3, TNFRSF14, PDCD1LG2, CD274, CD276, VTCN1, VSIR, HHLA2, BTNL2	10			
	Dual-function receptors	CD80, CD86, PVR, IL2RB	4			
Bioenergetics pathway enzymes (intracellular metabolism I-immunometabolism)	TCA cycle	CS, ACO1, ACO2, IDH2, IDH3A, OGDH, SUCLA2, SUCLG1, SUCLG2, SDHA, SDHB, FH, MDH2	13	56	23317369 25945836 26024507 25594225	Detailed information see Figure S4
	Pentose phosphate pathway	G6PD, PGLS, PGD, RPE, RPI, TALDO1, TKT	7			
	Glutamine pathway	SLC38A1, SLC38A2, GLS1, GLUD1, GOT2, GPT2, SLC1A5	7			
	Fatty Acid synthesis pathway	FATP, CD36, SLC27A1, SLC27A2, SLC27A3, SLC27A4, SLC27A5, SLC27A6, ACSL1, ACSL3, ACSL4, ACSL5, ACSL6, CPT1A, CPT1B, CPT2	16			
	Fatty Acid B-oxidation pathway	ACADVL, HADHA, HADHB, ACADS, ACADSB, ACADM, ACADL, ACAD8, ACAD9, ACAD10, ACAD11, ECHS1, HADH	13			
Trained immunity pathway enzymes (intracellular metabolism II-trained immunity)	Glycolysis pathway	GLUT1, HK, GPI, PFK1, ALDOA, TPI1, GAPDH, PGK, PGAM, ENO, PK, LDH, PDH1, MPC1	14	24–1 = 23	24911170 30298120 25594225	Detailed information see Figure S5
	Mevalonate metabolism pathway	ACLY, HMGCS1, HMGCR, MVK, PMVK, MVD, FDPS	7			
	Acetyl-CoA generating enzyme	ACLY, ACSL1, ACSL5	3			
Exosome biogenesis/docking mediators (local and distal cell-cell communication vehicles)	Biogenesis mediators	RAB11A, STX6, ARF6, RAB27A, RAB31, SEC22B, STX18, STX3, VAMP3, YKT6, TSG101, PDCD6IP	12	24	29109687	Detailed information see Figure S6
	Docking mediators	CAV1, CD44, SELE, ADGRE1, LGALS3, LGALS1, ICAM-1, ITGA6, ITGB1, ITGB3, ITGB4, LAMP1	12			
		Total number		207		

**Table 3A:** The expressions of 31 macrophage markers in 10 Mφ subsets are modulated in 8 groups of 34 diseases.

**Mφ**		**Autoimmune diseases**				**Cardiovascular diseases**				**Digestive inflammatory**				**Infection diseases**				**Metabolic diseases**				**Respiratory diseases**				**Digestive tumors**					**Other tumors**					**Up-regulated disease**		**Down-regulated disease**	
**Subsets**	**Markers**	**1**	**2**	**3**	**4**	**5**	**6**	**7**	**8**	**9**	**10**	**11**	**12**	**13**	**14**	**15**	**16**	**17**	**18**	**19**	**20**	**21**	**22**	**23**	**24**	**25**	**26**	**27**	**28**	**29**	**30**	**31**	**32**	**33**	**34**	**Number**	**%**	**Number**	**%**
M1	IL1B	↓				↑	↑	↓		↑		↑	↑	↓	↑	↓	↓								↑					↑		↓		↓		8	23.5	7	20.6
	TNF	↓									↑				↑		↓		↑					↓	↑			↓		↑			↑	↓		6	17.6	5	14.7
	IL6	↓				↑	↑			↑		↑	↑	↓	↑	↓									↑			↑						↓	↓	8	23.5	5	14.7
	CXCL11	↑				↑	↑				↑	↑	↑	↑		↑		↑									↑	↑		↑	↑		↑		↑	15	44.1	0	0.0
	CXCL10	↑				↑	↑				↑	↑	↑			↑											↑		↑	↑	↑				↑	12	35.3	0	0.0
	CXCL9	↑				↑	↑			↑	↑	↑	↑	↑												↑			↑	↑	↑				↑	13	38.2	0	0.0
	IL23A			↑		↓				↑		↑	↑													↓		↑		↑	↑		↑	↑	↓	9	26.5	3	8.8
	IL12A						↑																			↑				↓			↑	↓		3	8.8	2	5.9
	IL12B						↑									↓												↑		↑						3	8.8	1	2.9
	ARG2	↓		↑			↑					↓		↓				↓										↑				↓		↑	↓	4	11.8	6	17.6
M2a	MRC1			↓		↑	↑											↓					↑							↓			↑			4	11.8	3	8.8
	CD163		↑				↑	↓	↓		↑	↑	↑		↑							↑	↑					↓	↓	↓				↓	↑	9	26.5	6	17.6
	STAB1					↑	↓				↑				↑										↑	↓		↓		↑			↑		↑	7	20.6	3	8.8
	CCL18	↑				↑	↑				↑		↑													↑	↑	↓	↑	↑			↑	↓	↑	11	32.4	2	5.9
	CD200R1				↓	↓	↑								↓		↓											↓							↑	2	5.9	5	14.7
	F13A1			↓				↑			↑			↓									↑			↓		↓		↓	↓		↑	↓		4	11.8	7	20.6
	IL1RN	↑	↑			↑	↑		↑	↑		↑	↑		↑				↑		↓				↑	↓	↓		↑		↑		↑	↓	↑	15	44.1	4	11.8
	ARG1		↑	↑		↓	↑					↑			↑				↑				↑		↑	↓	↓									8	23.5	3	8.8
	PDE4DIP			↓	↓	↑	↑					↑	↑	↓				↓				↑				↑		↓		↓	↓		↓			6	17.6	8	23.5
	Chil4																																			0	0.0	0	0.0
	Chil3																																			0	0.0	0	0.0
	Retnla																																			0	0.0	0	0.0
M2b	IL10				↓	↑	↑						↑	↑											↑									↑	↑	7	20.6	1	2.9
	IL12B						↑																					↑		↑						3	8.8	0	0.0
	IL12A						↑																			↑				↓			↑	↓		3	8.8	2	5.9
M2c	MRC1			↓		↑	↑											↓					↑							↓			↑			4	11.8	3	8.8
	ARG1		↑	↑		↓	↑					↑			↑				↑				↑		↑	↓	↓									8	23.5	3	8.8
M2d	TNF	↓									↑				↑		↓		↑									↓		↑			↑	↓		5	14.7	4	11.8
	IL12A						↑																			↑				↓			↑	↓		3	8.8	2	5.9
	IL12B						↑									↓												↑		↑						3	8.8	1	2.9
M4	MMP7					↑				↑	↑	↑	↑			↓										↑		↑	↑		↑		↑		↓	10	29.4	2	5.9
	MRC1			↓		↑	↑											↓					↑							↓			↑			4	11.8	3	8.8
	S100A8	↑		↑			↓				↑	↑	↑							↓								↑		↑	↓			↓		7	20.6	4	11.8
Mox	HMOX1					↑	↑			↑			↓		↑	↑									↑	↑		↓			↑		↓	↓	↑	9	26.5	4	11.8
	NFE2L2					↑	↑		↓			↓		↓				↓					↑			↓						↓	↓			3	8.8	7	20.6
	TXNRD1					↑	↑	↓	↓					↓									↑				↑		↑							5	14.7	3	8.8
	SRXN1			↑		↑	↑						↑	↓													↑	↑		↑		↑				8	23.5	1	2.9
M(hb)	CD163		↑				↑	↓	↓		↑	↑	↑		↑							↑	↑					↓	↓	↓				↓	↑	9	26.5	6	17.6
	MRC1			↓		↑	↑											↓					↑							↓			↑			4	11.8	3	8.8
Mhem	CD163		↑				↑	↓	↓		↑	↑	↑		↑							↑	↑					↓	↓	↓				↓	↑	9	26.5	6	17.6
HA-mac	CD163		↑				↑	↓	↓		↑	↑	↑		↑							↑	↑					↓	↓	↓				↓	↑	9	26.5	6	17.6
	HLA-DRB1	↑					↓				↑		↑					↑					↓					↓					↑	↓	↑	6	17.6	4	11.8
	HLA-DRA	↑								↑	↑		↑								↓							↓			↑		↑	↓	↑	7	20.6	3	8.8
Up- regulated gene	Number	8	3	5	0	16	20	1	1	8	12	12	16	3	8	3	0	2	3	0	0	2	6	0	8	6	5	8	6	11	8	1	12	3	12				
	%	25.8	9.7	16.1	0.0	51.6	64.5	3.2	3.2	25.8	38.7	38.7	51.6	9.7	25.8	9.7	0.0	6.5	9.7	0.0	0.0	6.5	19.4	0.0	25.8	19.4	16.1	25.8	19.4	35.5	25.8	3.2	38.7	9.7	38.7				
Down-regulated gene	Number	4	0	3	3	3	3	3	3	0	0	2	1	8	1	4	3	4	0	1	2	0	1	1	0	6	2	10	1	5	3	3	3	12	4				
	%	12.9	0.0	9.7	9.7	9.7	9.7	9.7	9.7	0.0	0.0	6.5	3.2	25.8	3.2	12.9	9.7	12.9	0.0	3.2	6.5	0.0	3.2	3.2	0.0	19.4	6.5	32.3	3.2	16.1	9.7	9.7	9.7	38.7	12.9				

*First, some Mϕ markers were upregulated in more than 30% of the 34 diseases, including three M1 markers, CXCL11, CXCL10, and CXCL9, 2 M2 markers, CCL18 and IL1RN, and one M4 marker, MMP7. Second, the diseases having Mϕ markers upregulated in more than 30% of the 34 diseases were of eight types, namely #5 myocardial infarction, #6 coronary artery disease, #10 gastritis, #11 Crohn's ileitis, #12 Crohn's colitis, #29 esophageal cancer, #32 ovarian carcinoma, and #34 renal carcinoma (For detailed expression data, see Figures S9–S12). 1, Rheumatoid arthritis; 2, Systemic lupus erythematosus; 3, Psoriasis; 4, Asthma; 5, Myocardial infarction; 6, Coronary artery disease; 7, Abdominal aortic aneurysm; 8, Aortic occlusive disease; 9, Ulcerative colitis; 10, Gastritis; 11, Crohn's ileitis; 12, Crohn's colitis; 13, Tuberculous Meningitis coinfected with HIV; 14, Sepsis; 15, Chronic Hepatitis C virus; 16, Tuberculosis; 17, Type 2 diabetes; 18, Type 1 diabetes; 19, Metabolic syndrome; 20, Familial Hypercholesterolemia; 21, Chronic obstructive pulmonary disease; 22, Pulmonary arterial hypertension; 23, Asthma; 24, Pneumonia; 25, Gastric adenocarcinoma; 26, Hepatocellular cancer; 27, Colorectal adenocarcinoma; 28, Pancreatic cancer; 29, Esophageal cancer; 30, Breast Adenocarcinoma; 31, Prostate cancer; 32, Ovarian carcinoma; 33, Lung cancer; 34, Renal carcinoma*.

**Table 3B:** The expressions of 18 macrophage subset transcription factors and 27 macrophage general transcription factors are modulated in 8 groups of 34 diseases.

**Mφ**		**Autoimmune diseases**				**Cardiovascular diseases**				**Digestive inflammatory**				**Infection diseases**				**Metabolic diseases**				**Respiratory diseases**				**Digestive tumors**					**Other tumors**					**Up-regulated disease**		**Down-regulated disease**	
**Subsets**	**TFs**	**1**	**2**	**3**	**4**	**5**	**6**	**7**	**8**	**9**	**10**	**11**	**12**	**13**	**14**	**15**	**16**	**17**	**18**	**19**	**20**	**21**	**22**	**23**	**24**	**25**	**26**	**27**	**28**	**29**	**30**	**31**	**32**	**33**	**34**	**Number**	**%**	**Number**	**%**
M1	HIF1A							↓		↑		↑	↑	↓			↓	↓		↓			↑				↑							↑	↓	6	17.6	6	17.6
	RELA	↓												↓																		↑				1	2.9	2	5.9
	IRF3						↓																									↓	↑	↑		2	5.9	2	5.9
	STAT1	↑		↑	↑		↑		↓	↑		↑	↑	↓		↑	↑						↑				↑		↑	↑	↑		↑	↑	↑	17	50.0	2	5.9
	STAT2								↓				↑			↑	↑	↑											↑	↑		↑	↓	↑	↑	9	26.5	2	5.9
M2a	PPARD			↑		↑	↑			↑			↓															↓	↑	↑		↓	↑	↑		8	23.5	3	8.8
	PPARG	↓				↑	↑	↓	↓	↓			↓	↓	↑						↑				↑	↓	↑		↑		↓			↓		7	20.6	9	26.5
	KLF4	↓				↓				↓			↓	↓			↓									↓		↓	↑	↓	↓		↓	↓	↓	1	2.9	13	38.2
	AKT1													↓																			↑			1	2.9	1	2.9
M2b	MAPK1	↑					↑						↑	↓				↓									↑						↓	↓	↑	5	14.7	4	11.8
	STAT3			↑			↑		↓	↑			↑	↓			↑											↑		↑		↓	↑		↓	8	23.5	4	11.8
M2c	NFKB1						↑		↓					↓																						1	2.9	2	5.9
	NFKB2	↓								↑			↑													↓			↑	↑				↑	↑	6	17.6	2	5.9
	NR3C1				↓	↓	↑					↓	↑	↓				↓		↓								↓		↓	↓	↓		↓	↑	3	8.8	11	32.4
	NFE2					↑	↓		↑				↑	↓			↑								↑						↓		↑	↓		6	17.6	4	11.8
Mhb	ATF1						↑					↓	↓	↓				↓								↑		↓					↓			2	5.9	6	17.6
Mox	NR1H3	↑											↓																↑		↓		↑		↑	4	11.8	2	5.9
Mhem	NR1H2								↑					↓					↑		↓		↓						↑							3	8.8	3	8.8
	NR1H3	↑											↓																↑		↓		↑		↑	4	11.8	2	5.9
uncertain	CREB1	↑					↓							↓			↓	↓											↑	↑			↓	↑		4	11.8	5	14.7
	HMGA1					↑	↑		↑					↓						↓						↑	↑	↑	↑	↑	↑		↑	↑		11	32.4	2	5.9
	SMAD4					↓	↑	↓	↓			↑	↓	↓			↑	↓							↓	↓		↓	↓	↓		↓	↓			3	8.8	13	38.2
	ZNF148							↓	↓					↓				↓					↑				↑						↓	↑		3	8.8	5	14.7
	HBP1					↓			↓					↓				↓					↑					↓		↓			↓			1	2.9	7	20.6
	CKLF	↑					↑		↓				↑	↓				↓		↓						↑	↑		↑		↑		↑			8	23.5	4	11.8
	ZNF281						↑			↑			↑	↓												↑			↑	↑	↑	↑		↑		9	26.5	1	2.9
	FOXO3	↓						↓	↓																				↓	↓		↓				0	0.0	6	17.6
	HEY1					↑	↑		↓			↑		↓			↓			↑			↓		↑	↑		↓		↑	↓	↓		↓	↑	8	23.5	8	23.5
	ETS2	↓				↓	↓	↓	↓				↑	↓						↓					↑		↓	↑	↑	↑		↓	↓	↓		6	17.6	11	32.4
	HIF1A					↑	↓		↑	↑		↑	↑	↓			↓						↑				↑							↑	↓	8	23.5	4	11.8
	STAT4		↓										↑															↓							↑	2	5.9	2	5.9
	MELTF			↓		↑						↓	↑													↑			↑	↑			↑	↑		7	20.6	2	5.9
	BATF3											↑	↑																				↑			3	8.8	0	0.0
	NFE2								↑				↑	↓			↑		↑		↓				↑						↓		↑	↓		6	17.6	4	11.8
	NFKB1						↑		↓					↓																						1	2.9	2	5.9
	RIT1			↑		↑	↑			↑			↓	↓				↓					↑				↑	↓	↑			↓			↑	8	23.5	5	14.7
	HIVEP1						↑							↓							↓		↑													2	5.9	2	5.9
	JUNB	↓							↑				↑	↓			↓			↓	↑						↓		↑		↓			↓		4	11.8	7	20.6
	NFX1	↓					↑		↓			↑		↓			↑						↑													4	11.8	3	8.8
	FOXN3					↓		↓	↓			↓	↓	↓			↓	↑								↓		↓		↓	↓		↓			1	2.9	12	35.3
	STAT3			↑					↓	↑			↑	↓			↑	↓		↓								↑		↑		↓	↑		↓	7	20.6	6	17.6
	PWWP3A																																			0	0.0	0	0.0
	MXD4							↓	↓											↑								↓			↓			↓		1	2.9	5	14.7
	E2F3						↑		↓			↑	↑				↑									↑	↑	↑	↑	↑	↑	↑	↑	↑		13	38.2	1	2.9
	CEBPD	↓										↑	↑					↓			↓								↓			↓		↓		2	5.9	6	17.6
	NME1					↑	↑	↓	↓		↑	↑	↑	↓				↓								↑		↑		↑	↑	↑	↑	↑		12	35.3	4	11.8
Up- regulated gene	Number	5	0	4	1	8	18	0	4	7	1	9	18	0	2	2	7	2	1	2	2	0	7	0	4	8	9	5	17	13	6	5	13	19	12				
	%	12.2	0.0	9.8	2.4	19.5	43.9	0.0	9.8	17.1	2.4	22.0	43.9	0.0	4.9	4.9	17.1	4.9	2.4	4.9	4.9	0.0	17.1	0.0	9.8	19.5	22.0	12.2	41.5	31.7	14.6	12.2	31.7	46.3	26.8				
Down-regulated gene	Number	9	1	1	1	6	4	8	18	2	0	4	8	28	0	0	6	12	0	6	3	0	2	0	1	5	2	11	3	6	9	10	10	13	4				
	%	20.0	2.2	2.2	2.2	13.3	8.9	17.8	40.0	4.4	0.0	8.9	17.8	62.2	0.0	0.0	13.3	26.7	0.0	13.3	6.7	0.0	4.4	0.0	2.2	11.1	4.4	24.4	6.7	13.3	20.0	22.2	22.2	28.9	8.9				

*First, some Mφ transcription factors (TFs) were upregulated in more than 30% of the 34 diseases, including M1 TF STAT1 and three other Mφ TFs, namely HMGA1, E2F3, and NME1. Second, the diseases having Mφ TFs upregulated in more than 30% among the 34 diseases were of six types, namely #6 coronary artery disease, #12 Crohn's colitis, #28 hepatocellular cancer, #29 esophageal cancer, #32 ovarian carcinoma, and #33 lung cancer (For detailed expression data, see the Figures S13–S16). 1, Rheumatoid arthritis; 2, Systemic lupus erythematosus; 3, Psoriasis; 4, Asthma; 5, Myocardial infarction; 6, Coronary artery disease; 7, Abdominal aortic aneurysm; 8, Aortic occlusive disease; 9, Ulcerative colitis; 10, Gastritis; 11, Crohn's ileitis; 12, Crohn's colitis; 13, Tuberculous Meningitis coinfected with HIV; 14, Sepsis; 15, Chronic Hepatitis C virus; 16, Tuberculosis; 17, Type 2 diabetes; 18, Type 1 diabetes; 19, Metabolic syndrome; 20, Familial Hypercholesterolemia; 21, Chronic obstructive pulmonary disease; 22, Pulmonary arterial hypertension; 23, Asthma; 24, Pneumonia; 25, Gastric adenocarcinoma; 26, Hepatocellular cancer; 27, Colorectal adenocarcinoma; 28, Pancreatic cancer; 29, Esophageal cancer; 30, Breast Adenocarcinoma; 31, Prostate cancer; 32, Ovarian carcinoma; 33, Lung cancer; 34, Renal carcinoma*.

We then determined whether there are disease-specific signaling pathways and shared pathways based on the expression changes of Mφ subset markers and Mφ TFs in eight groups of 34 diseases and tumors. After analyzing the Ingenuity Pathway Analysis results of the top 10 pathways in both upregulated and downregulated Mφ subset markers and Mφ TFs, respectively, we compared all the upregulated pathways, downregulated pathways, and the pathways either upregulated or downregulated in some diseases (upper panel, middle panel, and lower panel of Tables 3C,D). As shown in Table 3C, we found three disease-specific pathways upregulated and 14 disease-specific pathways downregulated. As shown in Table 3D, we found 16 disease-specific pathways upregulated and 16 disease-specific pathways downregulated. We also compiled a list of pathways that are shared in several groups of diseases and tumors.

**Table 3C:** Ingenuity Pathway Analyses showed that the top 10 pathways involved in 31 macrophage markers of 10 Mφ subsets are modulated in 8 groups of 34 diseases.

**Pathways modulated by Mφ** **markers**		**Disease type**	**Autoimmune diseases**	**Cardiovascular diseases**	**Digestive inflammatory disease**	**Infection disease**	**Metabolic diseases**	**Respiratory disease**	**Digestive tumors**	**Other tumors**
Up-regulated pathways in eight group of diseases	Hematopoiesis from Pluripotent Stem Cells	1		↑						
	IL-17 Signaling	1								↑
	Acute Phase Response Signaling	1						↑		
	Glucocorticoid Receptor Signaling^⋆^	2				↑		↑		
	IL-17A Signaling in Gastric Cells^⋆^	3	↑			↑				↑
	Pathogenesis of Multiple Sclerosis	6	↑	↑		↑	↑		↑	↑
	Agranulocyte Adhesion and Diapedesis	7	↑	↑	↑	↑	↑		↑	↑
	Granulocyte Adhesion and Diapedesis	7	↑	↑	↑	↑	↑		↑	↑
	N(↑)		4	4	2	5	3	2	3	5
Down-regulated pathways in eight group of diseases	Role of Pattern Recognition Receptors in Recognition of Bacteria and Viruses	1	↓							
	TREM1 Signaling	1	↓							
	IL-12 Signaling and Production in Macrophages^⋆^	1		↓						
	Role of BRCA1 in DNA Damage Response^⋆^	1			↓					
	Sirtuin Signaling Pathway^⋆^	1			↓					
	Unfolded protein response^⋆^	1			↓					
	Extrinsic Prothrombin Activation Pathway	1				↓				
	NRF2-Mediated Oxidative Stress Response^⋆^	1				↓				
	Thioredoxin Pathway	1				↓				
	Vitamin-C Transport	1				↓				
	Production of Nitric Oxide and Reactive Oxygen Species in Macrophages	1					↓			
	Aryl Hydrocarbon Receptor Signaling	1								↓
	Atherosclerosis Signaling	1								↓
	Xenobiotic Metabolism Signaling	1								↓
	Allograft Rejection Signaling	2						↓	↓	
	Calclum-induced T Lymphocyte Apoptosis	2						↓	↓	
	OX40 Signaling Pathway	2						↓	↓	
	T Helper Cell Differentiation	2						↓	↓	
	Role of IL-17A in Psoriasis	2		↓			↓			
	CD40 Signaling^⋆^	2			↓		↓			
	Arginine Degradaion VI(Arginase 2 Pathway)	3			↓	↓	↓			
	Antigen Presentation Pathway	3		↓				↓	↓	
	Autoimmune Thyroid Disease Signaling	3		↓				↓	↓	
	B Cell Development	3		↓				↓	↓	
	Nur77 Signaling in T Lymphocytes	3		↓				↓	↓	
	Citrulline Biosynthesis	3			↓	↓	↓			
	Urea Cycle	3			↓	↓	↓			
	Superpathway of Citrulline Metabolism	3			↓	↓	↓			
	Arginine Degradation I (Arginase Pathway)	3			↓	↓	↓			
	N(↓)		2	6	9	9	8	8	8	3
Dual-regulated pathways in eight groups of diseases	Hepatic Fibrosis/Hepatic Stellate Cell Activation	2	↓							↑
	Role of IL-17F in Allergic Inflammatory Airway Diseases^⋆^	2							↑	↓
	Dendritic Cell Maturation^⋆^	3	↑	↓	↑					
	Differential Regulation of Cytokine Production in Intestinal Epithelial Cells by IL-17A and IL-17F	3	↓					↑		↑
	LXR/RXR Activation	3					↓		↑	↓
	Altered T Cell and B Cell Signaling in Rheumatoid Arthritis	5	↑↓	↑↓	↑		↑	↑↓		
	Differential Regulation of Cytokine Production in Macrophages and T Heiper Cells by IL-17A and IL-17F	6	↓	↑	↑		↑		↑	↑↓
	Neuroinflammation Signaling Pathway	6	↓			↑↓	↑	↑	↓	↑↓
	Graft-vs.-Host Disease Signaling	7	↑	↓	↑		↑	↑↓	↓	↓
	IL-10 Signaling	7	↓	↑	↑↓	↑	↑↓	↑	↑	
	Role of Cytokines in Mediating Communication between Immune Cells	7	↑↓	↑↓	↑	↑		↑	↑	↓
	Communication between Innate and Adaptive Immune Cells	8	↑↓	↑	↑	↑	↑	↑	↑	↑
	Role of Hypercytokinemia/Hyperchemokinemia in the Pathogenesis of Influenza	8	↑	↑	↑	↑	↑	↑	↑	↓
	N(↑)		3	4	7	4	6	6	7	3
	N(↓)		5	2	0	0	1	0	2	5
	N(↑↓)		3	2	1	1	1	2	0	2

⋆ *The pathways modulated in 8 groups of 34 diseases both by 31 Mφ markers and 41 Mφ transcription factors*.

**Table 3D:** Ingenuity Pathway Analyses showed that the top 10 pathways involved in 18 macrophage subset transcription factors and 27 macrophage general transcription factors are modulated in 8 groups of 34 diseases.

**Pathways modulated by Mφ** **TFs**		**Disease type**	**Autoimmune diseases**	**Cardiovascular diseases**	**Digestive inflammatory disease**	**Infection disease**	**Metabolic diseases**	**Respiratory disease**	**Digestive tumors**	**Other tumors**
Up-regulated pathways in eight group of diseases	Role of JAK1 and JAK3 in γc Cytokine Signaling	1	↑							
	CNTF Signaling	1	↑							
	Thrombopoietin Signaling	1	↑							
	EGF Signaling	1	↑							
	GM-CSF Signaling	1	↑							
	IL-17A Signaling in Gastric Cells^⋆^	1		↑						
	NRF2-Mediated Oxidative Stress Response^⋆^	1		↑						
	Parkinson's Signaling	1		↑						
	Cyclins and Cell Cycle Regulation	1				↑				
	Cell Cycle Regulation by BTG Family Proteins	1				↑				
	Estrogen-Mediated S-phase Entry	1				↑				
	Role of CHK Proteins in Cell Cycle Checkpoint Control	1				↑				
	Notch Signaling	1					↑			
	Adrenomedullin Signaling Pathway	1						↑		
	IL-15 Production	1								↑
	Role of PKR in Interferon Induction and Antiviral Response	1								↑
	Interferon Signaling	2				↑				
	Oncostatin M Signaling	2	↑						↑	
	Tec Kinase Signaling	2			↑					↑
	Dendritic Cell Maturation^⋆^	3			↑				↑	↑
	Role of JAK family kinases in IL-6-type Cytokine Signaling	4	↑		↑			↑	↑	
	IL-22 Signaling	5	↑	↑	↑			↑	↑	
	N(↑)		8	4	4	5	2	3	4	4
Down-regulated pathways in eight group of diseases	ERK5 Signaling	1		↓						
	HGF Signaling	1		↓						
	FGF Signaling	1		↓						
	IGF-1 Signaling	1		↓						
	VDR/RXR Activation	1			↓					
	FXR/RXR Activation	1			↓					
	Apelin Endothelial Signaling Pathway	1				↓				
	Estrogen-Dependent Breast Cancer Signaling	1				↓				
	ILK Signaling	1				↓				
	NGF Signaling	1				↓				
	Prostate Cancer Signaling	1				↓				
	Factors Promoting Cardiogenesis in Vertebrates	1						↓		
	HIPPO Signaling	1						↓		
	Regulation of IL-2 Expression in Activated and Anergic T Lymphocytes	1						↓		
	TGF-β Signaling	1						↓		
	Cancer Drug Resistance By Drug Efflux	1							↓	
	BMP Signaling Pathway	2					↓	↓		
	Role of IL-17F in Allergic Inflammatory Airway Diseases^⋆^	2				↓	↓			
	PXR/RXR Activation	2	↓						↓	
	Sumoylation Pathway	2						↓	↓	
	Antiproliferative Role of TOB in T Cell Signaling	3						↓	↓	↓
	Cardiomyocyte Differentiation via BMP Receptors	3						↓	↓	↓
	Glucocorticoid Receptor Signaling^⋆^	4		↓			↓		↓	↓
	MIF-mediated Glucocorticoid Regulation	4	↓			↓	↓		↓	
	N(↓)		2	5	2	7	4	8	7	3
Dual-regulated pathways in eight group of diseases	Adipogenesis Pathway	2						↑		↓
	Cell Cycle: G1/S Checkpoint Regulation	2				↑		↓		
	Chronic Myeloid Leukemia Signaling	2						↓		↑
	PEDF Signaling	2	↓				↑			
	Role of BRCA1 in DNA Damage Response^⋆^	2			↓	↑				
	Th17 Activation Pathway	2	↓		↑					
	Thyroid Cancer Signaling	2			↓		↑			
	Activation of IRF by Cytosolic Pattern Recognition Receptors	3		↓		↑	↑			
	CD40 Signaling^⋆^	3		↑	↓		↓			
	IL-12 Signaling and Production in Macrophages^⋆^	3	↓					↑		↑
	LPS-Stimulated MAPK Signaling	3		↑		↓	↓			
	PPAR Signaling	3	↓	↑	↓					
	IL-17A Signaling in Fibroblasts	4	↓		↑		↓		↓	
	iNOS Signaling	4		↑		↓			↑	↑
	Osteoarthritis Pathway	4	↓					↑	↓	↓
	Pancreatic Adenocarcinoma Signaling	4			↑		↓		↑	↑
	PI3K Signaling in B Lymphocytes	4		↑		↓	↓			↓
	Polyamine Regulation in Colon Cancer	4			↓		↑	↑		↓
	Sirtuin Signaling Pathway^⋆^	4	↓		↓			↑	↑	↓
	Unfolded Protein Response^⋆^	4			↓		↑		↓	↓
	FLT3 Signaling in Hematopoietic Progenitor Cells	5	↑	↓	↑		↑		↑	
	ERK/MAPK Signaling	6	↑	↑↓	↓		↓	↑		↓
	JAK/Stat Signaling	7	↓	↓	↑	↑	↑		↑	↑
	Role of JAK1, JAK2 and TYK2 in Interferon Signaling	7		↓	↑	↑	↑	↑	↑	↑
	N(↑)		2	5	6	5	8	7	6	6
	N(↓)		8	4	8	3	6	2	3	7
	N(↑↓)		0	1	0	0	0	0	0	0

⋆ *The pathways modulated in 8 groups of 34 diseases both by 31 Mφ markers and 41 Mφ transcription factors*.

As shown in Table 3A, we found that among 21 disease-upregulated pathways, one pathway communication between innate and adaptive immune cells is shared among eight groups of diseases. We also found that three pathways, namely the role of hypercytokinemia/hyperchemokinemia in the pathogenesis of influenza, agranulocyte adhesion and diapedesis, and granulocyte adhesion and diapedesis, are shared by seven groups of diseases; four pathways, namely differential regulation of cytokine production in Mφ and T helper cells by IL-17A and IL-17F, IL-10 signaling, the role of cytokines in mediating communication between immune cells, and pathogenesis of multiple sclerosis, are shared by 7 groups of diseases; and one pathway, altered T cell and B cell signaling in rheumatoid arthritis, is shared by five groups of diseases. In contrast, as shown in Table 3B, among 42 disease-downregulated pathways, 22 (52%) pathways are shared by two or more groups of disease, and 20 disease-specific downregulated pathways may be important for the pathogenesis of the diseases. Furthermore, as shown in Table 3C, 13 Mφ pathways are upregulated and/or downregulated in some disease groups in two different directions, suggesting that some Mφ functional pathways are modulated in disease-specific manners.

These results suggest that the expression changes of Mφ TFs in eight groups of 34 diseases are more disease-specific than that of Mφ subset markers, allowing the identification of 20 disease-specific and 12 shared (more than 4 groups of diseases) modulations of Mφ TFs pathways in eight groups of 34 diseases. As shown in Table 3E, in detail, we found five upregulated disease-specific pathways in autoimmune diseases, three upregulated disease-specific pathways in cardiovascular diseases, two upregulated disease-specific pathways in digestive inflammatory diseases, four upregulated disease-specific pathways in infectious diseases, one disease-specific pathway in metabolic disease, one disease-specific pathway in respiratory disease, one upregulated pathway (shared with autoimmune disease) and one downregulated specific pathway in digestive tumors, and two upregulated disease-specific pathways in other tumors. In addition, we found 12 pathways that are shared by more than four groups of diseases and tumors. These results demonstrate for the first time that the expressions of Mφ TFs are modulated in both disease-specific, and shared signaling pathways; these results provide insights on the roles of Mφ in various diseases and novel therapeutic targets for modulating Mφ TFs and Mφ functions for those diseases and tumors. These results have also demonstrated for the first time that certain “high hierarchical” functional pathways in pathological Mφ are more important in the pathogenesis of various diseases than other pathways, making them novel pathological Mφ-specific therapeutic pathways; disease-specific Mφ pathways are also important for the pathogenesis of the diseases and are disease-specific therapeutic targets.

**Table 3E:** Twenty new disease group-specific and 12 shared (more than 4 groups of diseases) Mφ reprogramming pathways have been identified in eight groups of 34 diseases and tumors.

**A. Specific pathways (upregulated except #; unique for each group of diseases)**	
Autoimmune diseases	Role of JAK1 and JAK3 in γc Cytokine Signaling
	CNTF Signaling
	Thrombopoietin Signaling
	EGF Signaling
	GM-CSF Signaling
Cardiovascular diseases	IL-17A Signaling^*^
	NRF2-Mediated Oxidative Stress Response
	Parkinson's Signaling
Digestive inflammatory disease	VDR/RXR Activation#
	FXR/RXR Activation#
Infection disease	Cyclins and Cell Cycle Regulation
	Cell Cycle Regulation by BTG Family Proteins
	Estrogen-mediated S-phase Entry
	Role of CHK Proteins in Cell Cycle Checkpoint Control
Metabolic diseases	Notch Signaling
Respiratory disease	Adrenomedullin signaling pathway
Digestive tumors	Oncostatin M Signaling
	Cancer Drug Resistance By Drug Efflux (#, downregulated)
Other tumors	IL-15 Production
	Role of PKR in Interferon Induction and Response
**B. Shared pathways (upregulated and shared by more than four major disease groups)**	
Altered T Cell and B Cell Signaling in Autoimmune Disease	
Differential Regulation of Cytokine Production in Macrophages and T Heiper Cells by IL-17A and IL-17F	
Neuroinflammation Signaling Pathway	
Graft-vs.-Host Disease Signaling	
IL-10 Signaling	
Role of Cytokines in Mediating Communication between Immune Cells	
Communication between Innate and Adaptive Immune Cells	
Role of Hypercytokinemia/Hyperchemokinemia in the Pathogenesis of Disease	
FLT3 Signaling in Hematopoietic Progenitor Cells	
ERK/MAPK Signaling	
JAK/Stat Signaling	
Role of JAK1, JAK2 and TYK2 in Interferon Signaling	

* *Some of the pathway names were simplified to avoid potential confusion*.

### Macrophages (Mφ) in Lung, Liver, Spleen, and Intestine Express Higher M1 Mφ Markers Than Lean Adipose Tissue in Physiological Conditions

**Figure 2:**
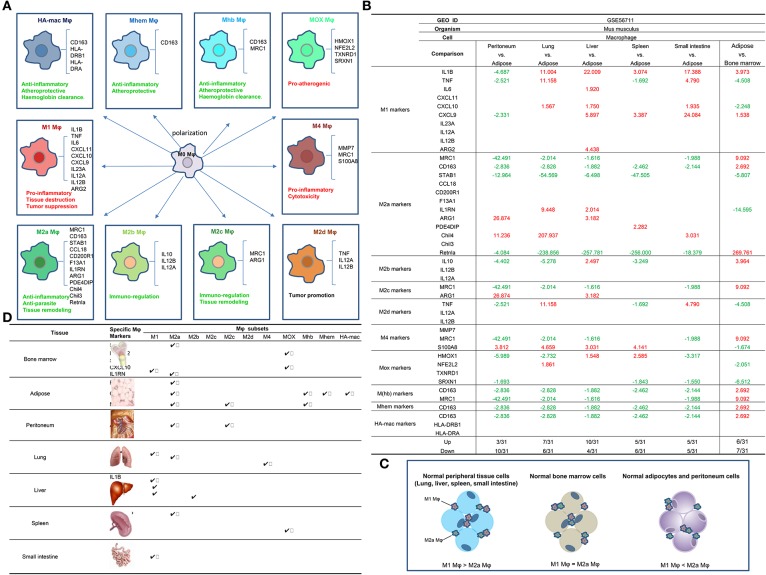
**(A)** Macrophages (Mφ) can be polarized into 10 (potentially more) different subsets, and the markers and main functions of 10 Mφ subsets are different (PMID: 24998279; 25319333; 25973901). M1, M4, and Mox are proinflammatory while the rest of the Mφ subsets are anti-inflammatory. **(B) Mechanism I:** The 10 macrophage (Mφ) subset markers (30) are differentially expressed in macrophages from various tissues, and lung, liver, spleen, and small intestine upregulate more M1 Mφ markers than M2 Mφ markers in the physiologic condition in comparison to lean ATMφ. (1) Retnla, CD163, and MRC1 are relatively adipose tissue-specific Mφ (ATMφ) markers; (2) STAB1, NFE2L2, and SRXN1 are relatively bone marrow-specific Mφ markers; (3) ARG1 is a relatively specific Mφ marker for peritoneum, Chil4 is a relatively specific Mφ marker for lung, IL1B is a relatively specific Mφ marker for liver, PDE4DIP and HMOX1 are relatively specific Mφ markers for spleen, and CXCL9 is a relatively specific Mφ marker for small intestine. **(C)** Mφ subset markers are differentially expressed in various tissues. **(D)** Tissue Mφ have different compositions of Mφ subsets as judged by the expressions of Mφ subset markers. (1) The genes of macrophage subtypes such as Mhb, Mhem, and HA-mac are relatively adipose tissue-specific. (2) The genes of Mox are bone marrow macrophage-specific. (3) Retnla is a M2a subset marker in peripheral tissues. (4) ILIB and CXCL9 are specific markers for small intestine M1 macrophages. (5) ARG1 is a specific marker for peritoneal M2a and M2c macrophages.

To determine the novel mechanisms underlying disease-specific and shared macrophage pathways, we and others previously reported that metabolic disease risk factors serve as conditional danger-associated molecular patterns (conditional DAMPs) (12, 31, 32) and induce monocyte/Mφ differentiation into Ly6Chigh-(1-3) and CD40+ proinflammatory monocytes (4), and accelerate vascular inflammation. Other studies also reported that, under certain experimental conditions such as stimulation with lipopolysaccharide (LPS) and interferon-γ (IFN-γ) for M1 polarization or interleukin-4 (IL-4) for M2 polarization (33), Mφ can be polarized into multiple subsets including proinflammatory M1 and M4 Mφ, anti-inflammatory M2, M(Hb), and Mhem Mφ (34) (Figure 2A). However, whether the different physiological environments present in tissues affect the expression of residential Mφ subsets markers and other regulators has not been studied (35). We hypothesized that various tissue environments with tissue differentiation potentials, DAMPs/conditional DAMPs, cytokines, and cell-cell contacts induce differential expression patterns of Mφ subset markers.

To examine this hypothesis in a very comprehensive manner, we collected 31 Mφ markers of 10 Mφ subsets, as reported in a recent publication (9) (Table 2). As summarized in Figure 2A, the 10 Mφ subsets perform various functions in regulating inflammation, immune responses, anti-oxidant, and tumor promotion (9). We included 10 Mφ markers in our analysis, and these markers are differentially expressed in different Mφ subsets. Furthermore, we also made a list of the transcription factors (TFs) of 18 Mφ subsets, which are critical for the development and maintenance of seven out of 10 Mφ subsets (Table 2). The data for TFs that are critical for development of M2d, M4, and HA-Mac were not available at the time we conducted the analysis. Moreover, we included an additional 27 Mφ TFs that were identified in Mφ by RNA-Seq analysis (21).

By examining the expression of these Mφ regulators in Mφ microarray datasets deposited in the NI-NCBI GEO DataSets database (https://www.ncbi.nlm.nih.gov/gds/), we found that the expression of M1 Mφ markers is higher in Mφ that reside in tissues such as lung, liver, spleen, and intestine (LLSI) compared to lean adipose tissue Mφ (ATMφ), physiologically (Figure 2B). This suggests that the majority of lung, liver, spleen, and intestine (LLSI) residential Mφ are M1 Mφ (Figure 2C); therefore, these tissues have more potential to produce inflammatory responses than adipose tissue. In addition, as shown in Figures 2B,D, we found that: (1) Retnla, CD163, and MRC1 are relatively ATMφ-specific markers; (2) STAB1, NFE2L2, and SRXN1 are relatively bone marrow (BM)-specific Mφ markers; (3) ARG1 is a relatively specific Mφ marker for peritoneum, M2a, and M2c; (4) Chil4 is a relatively specific Mφ marker for lung and M2a; (5) IL1B is a relatively specific Mφ marker for liver and M1; (6) PDE4DIP and HMOX1 are relatively specific Mφ markers for spleen, M2a, and MOX; and (7) CXCL9 is a relatively specific Mφ marker for small intestine and M1. Our results suggest that these Mφ subset markers modulated in the tissues may play important roles in tissue-specific Mφ functions and subset compositions.

### Pro-adipogenic Transcription Factors C/EBPα and PPARγ, and Proinflammatory Adipokine Leptin Upregulate the Expression of M1 Mφ Markers

Adipose tissue releases more than 50 hormones, cytokines, and chemokines, collectively called adipokines, which regulate several physiological processes concerning energy, glucose metabolism, and immunity in an autocrine, paracrine, or systemic manner as well as several pathological processes including proinflammatory or anti-inflammatory processes, thereby contributing to insulin resistance and other inflammations (36). Adipose tissue from lean individuals releases anti-inflammatory adipokines such as adiponectin, transforming growth factor-β (TGF-β), IL-10, IL-4, IL-13, IL-1 receptor antagonist (IL-1Ra), and apelin. In contrast, obese adipose tissue secretes proinflammatory cytokines such as tumor necrosis factor-α (TNF-α), IL-6, leptin, visfatin, resistin, angiotensin II, and plasminogen activator inhibitor 1 (37). About one-third of obese adults and 10% of non-obese adults are metabolically healthy obese (MHO) (38, 39). A series of reports suggest that patients with metabolically healthy obesity (MHO) have significantly higher rates of type II diabetes (40), metabolic syndrome (41), and chronic kidney disease (42) than metabolically healthy lean individuals. The molecular mechanisms underlying the pathogenesis of MHO remained poorly determined. In the search for master regulators responsible for MHO with the features of being pro-inflammatory/proatherogenic but anti-adipogenic, we reported that microRNA-155 (miR155) and, potentially, microRNA-221 are such master regulators for MHO(44). Deficiencies in those master regulators such as miR155 in an atherogenic apolipoprotein E (ApoE)^−/−^ background led to the establishment of MHO in mice, significantly improving our understanding of the molecular mechanisms underlying MHO (43). Our recent findings further suggest that elevated adipokine resistin and leptin in a miR155^−/−^/ApoE^−/−^ MHO model fed a high-fat diet for 12 weeks may serve as a driver for the newly termed “second wave of inflammation status” in the MHO model (44). Along the same line, the issue of whether proinflammatory adipokines secreted by obese adipose tissues promote the expression of M1 Mφ markers and other proinflammatory regulators remained poorly defined.

We hypothesize that proinflammatory cytokine interferon-γ (IFN-γ) and lipopolysaccharide (LPS) upregulate M1 markers and regulators but not M2 (45). To test this hypothesis, we examined the expression of Mφ markers and Mφ TFs involved in M1 and M2 Mφ polarization. As shown in Table 4A, when we examined the TF expression in the Mφ polarization from human CD14+ monocytes, we made the following important findings: (a) M1 polarization promotes the expression of the Mφ TFs for M1 and surprisingly also for M2c (IL-10 polarization); (b) the Krüppel-like family of transcription factor 4 (KLF4) was upregulated explicitly during M2a polarization (IL-4 polarization); and (c) four proinflammatory TFs (STAT1, STAT2, STAT3, and NF-kB) are more upregulated in M1 than in M2 polarization, suggesting their importance in promoting M1 Mφ polarization (46).

**Table 4A:** **Mechanism II:** M1 polarization promotes the expressions of M1- and M2c-transcription factors (TFs; NFkB1, NFkB2, and NR3C1) but inhibits the expression of M2a TF PPARg; M2 polarization specifically upregulates M2a TF KLF4, and M1 macrophages (Mφ) express higher levels of TFs, STAT1, STAT2, STAT3, and NF-kB than M0 Mφ.

	**GEO ID**	**GSE85346**			
	**Comparision**	**M1 vs. M0**	**M2a vs. M0**	**M2b vs. M0**	**M2c vs. M0**
M1 TFs	HIF1A	2.247			
	RELA	5.401			
	IRF3	2.132			
	STAT1	9.433		3.077	
	STAT2	3.353		1.787	
M2a TFs	PPARD				
	PPARG	−14.550		−1.988	
	KLF4		4.123		
	AKT1				
M2b TFs	MAPK1				
	STAT3	5.058			1.699
M2c TFs	NFKB1	4.065		1.787	
	NFKB2	8.639			
	NR3C1	2.439			
	NFE2				
Mox TFs	NR1H3				
M(hb) TFs	ATF1				
Mhem TFs	NR1H3				
	NR1H2				
	Up	9/18	1/18	3/18	1/18
	Down	1/18	0/18	1/18	0/18

In addition, as shown in Table 4B, we also determined whether pro-adipogenic TFs, proinflammatory, and anti-inflammatory adipokines can regulate Mφ subset marker expression. The results showed the following. (1) pro-adipogenic TFs CCAAT/enhancer-binding protein α (C/EBPα) and peroxisome proliferator-activated receptor γ (PPARγ) promote the expression of M1 markers interleukin-1β (IL-1β), tumor necrosis factor-α (TNF-α), and C-X-C motif chemokine 10 (CXCL10), suggesting that during adipogenesis, pro-adipogenic TF-mediated signaling mechanisms have the potential to promote M1 subset polarization. Of note, previous reports found that C/EBPα (47) and PPARγ (48) promote M2 polarization. One of our explanations is that pro-adipogenic TFs C/EBPα and PPARγ may tend to promote M2 in lean adipose tissues but that, in hypertrophic obese adipose tissues, these TFs may promote polarization of proinflammatory M1. Further detailed transcriptomic studies will be required to address this discrepancy. (2) C/EBPα suppresses the expression of the M2a markers stabilin 1 (STAB1), coagulation factor XIII A chain (F13A1), chitinase-like 4 (Chil4), and Chil3. (3) C/EBPβ also suppresses the expression of the M2a marker arginase 1 (ARG1). (4) Deficiencies in anti-inflammatory adopkines such as secreted frizzled-related protein 5 (SFRP5) and adiponectin do not change the expression markers of all 10 types of Mφs, suggesting that the anti-inflammatory regulation of these adipokines acts via Mφ composition modulation-independence mechanisms. (5) Proinflammatory adipokine leptin promotes M1 marker gene expression and inhibits the marker expressions of M2 and other Mφ subsets.

**Table 4B:** Proadipogenic transcription factors C/EBPa and PPARg promote the expression of M1 macrophage markers, C/EBPa and C/EBPb inhibit the expressions of M2 macrophage markers, and higher expressions of Mhb, Mhem, and HA-mac subtype markers in adipose tissues may result from stimulation in adipose tissue environments rather than that in adipogenesis.

		**Adipose transcription factors deficient**			**Anti-inflammatory adopkine deficient**		**Proinflammatory adipokine deficient**		
	**GEO ID**	**GSE55760**	**GSE59585**	**GSE14004**	**GSE37514**	**GSE50183**	**GSE66073**	**GSE46320**	**GSE27017**
	**Comparision**	**C/EBPa KD**	**C/EBPb KO**	**PPARg siRNA**	**SFRP5 KO**	**adiponectin deficient**	**APJ ko**	**PAI-1 KO**	**Leptin deficiency**
M1 markers	IL1B	−1.641							2.204
	TNF	−1.651							
	IL6			2.703					−9.353
	CXCL11								
	CXCL10			−1.801					−15.123
	CXCL9								−2.723
	IL23A								
	IL12A								
	IL12B								
	ARG2								1.612
M2a markers	MRC1								7.143
	CD163								
	STAB1	1.534							8.693
	CCL18								
	CD200R1								12.597
	F13A1	3.694					3.926		2.446
	IL1RN								3.095
	ARG1		1.582						3.675
	PDE4DIP			−13.880					
	Chil4	12.446							
	Chil3	11.791							2.351
	Retnla								−16.512
M2b markers	IL10						−1.822		
	IL12B								
	IL12A								
M2c markers	MRC1								7.143
	ARG1		1.582						3.675
M2d markers	TNF	−1.651							
	IL12A								
	IL12B								
M4 markers	MMP7								
	MRC1								7.143
	S100A8	5.599		−9.351					2.345
Mox markers	HMOX1								2.025
	NFE2L2	1.647		−2.126					
	TXNRD1								
	SRXN1								
M(hb) markers	CD163								
	MRC1								7.143
Mhem markers	CD163								
HA-mac markers	CD163								
	HLA-DRB1								
	HLA-DRA								
	Up	6/31	1/31	1/31	0/31	0/31	1/31	0/31	11/31
	Down	2/31	0/31	4/31	0/31	0/31	1/31	0/31	4/31

### Tissue Mφ From Lung, Liver, Spleen, Intestine, and Bone Marrow (BM) Express Higher Levels of T Cell Co-inhibition Receptor CD274 (PDL-1) Among 10 Co-inhibition Receptors Than That of Lean Adipose Tissues

**Figure 3:**
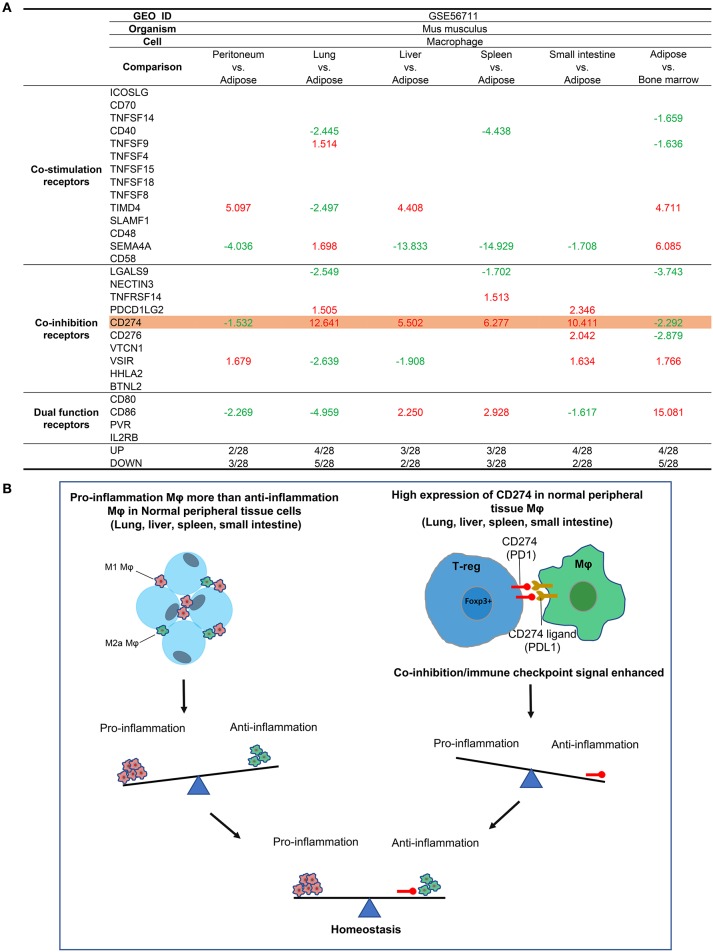
**Mechanism III:** The macrophages (Mφ) from lung, liver, spleen, and intestine have differences in the expressions of T cell co-stimulation receptors, co-inhibition/immune checkpoint receptors, and dual-function receptors in comparison to that of ATMφ. **(A)** First, Mφ from lung, liver, spleen and intestine express CD274 much higher than adipose tissue macrophages; second, the Mφ from peritoneum and adipose tissue express lower levels of CD274 than that of bone marrow, suggesting that decreased expression of CD274 is a remarkable feature of adipose tissue macrophages; third, lung Mφ upregulates the expression of TNFSF9, SEMA4A (co-stimulation), and PDCD1GL2 in comparison to lean ATMφ; and fourth, liver Mφ upregulates TIMD4 (co-stimulation) and CD86 (dual) in comparison to lean ATMφ. **(B)** The proposed model of **A**.

Since Mφs are prototypic professional antigen-presenting cells (APCs) that modulate CD4+ T cell activation by providing T cell activation signal #1 and co-stimulation/co-inhibition-based signal #2(50), we also examined the expression of 28 T cell co-stimulation and co-inhibition (immune checkpoint) receptors (23), including 14 co-stimulation receptors, 10 co-inhibition receptors, and 4 dual-functional (both co-stimulation and co-inhibition) receptors in tissue Mφ (Figure 3A), as we reported previously (49). As shown in Figure 3A, we found that: (i) the Mφ from LLSI express co-inhibition receptor CD274 (programmed death-ligand 1, PDL1) in much higher levels than ATMφ and (ii) the Mφ from peritoneum and ATMφ express lower levels of CD274 than BM Mφ. It has been reported that CD274 has significant reverse signaling activities (50). Antitumor immune response-enhancing transcription factor Forkhead box O (FoxO) inhibits CD274 expression (51), suggesting that CD274 expression may be responsible via reverse signaling for hiding immune response-enhancing features of tumor cells. Also, CD274 signals via conserved intracellular sequence motif “RMLDVEKC” inhibit JAK1-induced STAT3 activation and overcome interferon-mediated cytotoxicity (50). To correlate with the reported findings, our results suggest that: (i) peripheral tissue Mφ, including LLSI Mφ, express higher levels of T cell co-inhibition receptor CD274 than ATMφ, to contribute to the establishment of immune tolerance at physiological conditions; and (ii) since our data suggested that LLSI tissue Mφ are more proinflammatory than other Mφ, higher expression of CD274 in LLSI Mφ suggests that the high homeostatic and anti-inflammatory functions of CD274 (programmed death-ligand 1, PD-L1) via its reverse signaling in Mφ (52) may counteract the tissue Mφ proinflammatory status (Figure 3B) in addition to CD274 inhibition of T cell activation via PD-1 (programmed cell death protein 1, CD279) binding-mediated forward signaling (53, 54).

### Tissue Mφ From Lung, Liver, Spleen, Intestine, and Bone Marrow Prefer to Use RAB27A and STX3 Than RAB31 and YKT6 in Mediating Exosome Biogenesis and Docking, Suggesting New Inflammatory Exosome Markers and a New Inflammatory Exosome Status

**Figure 4:**
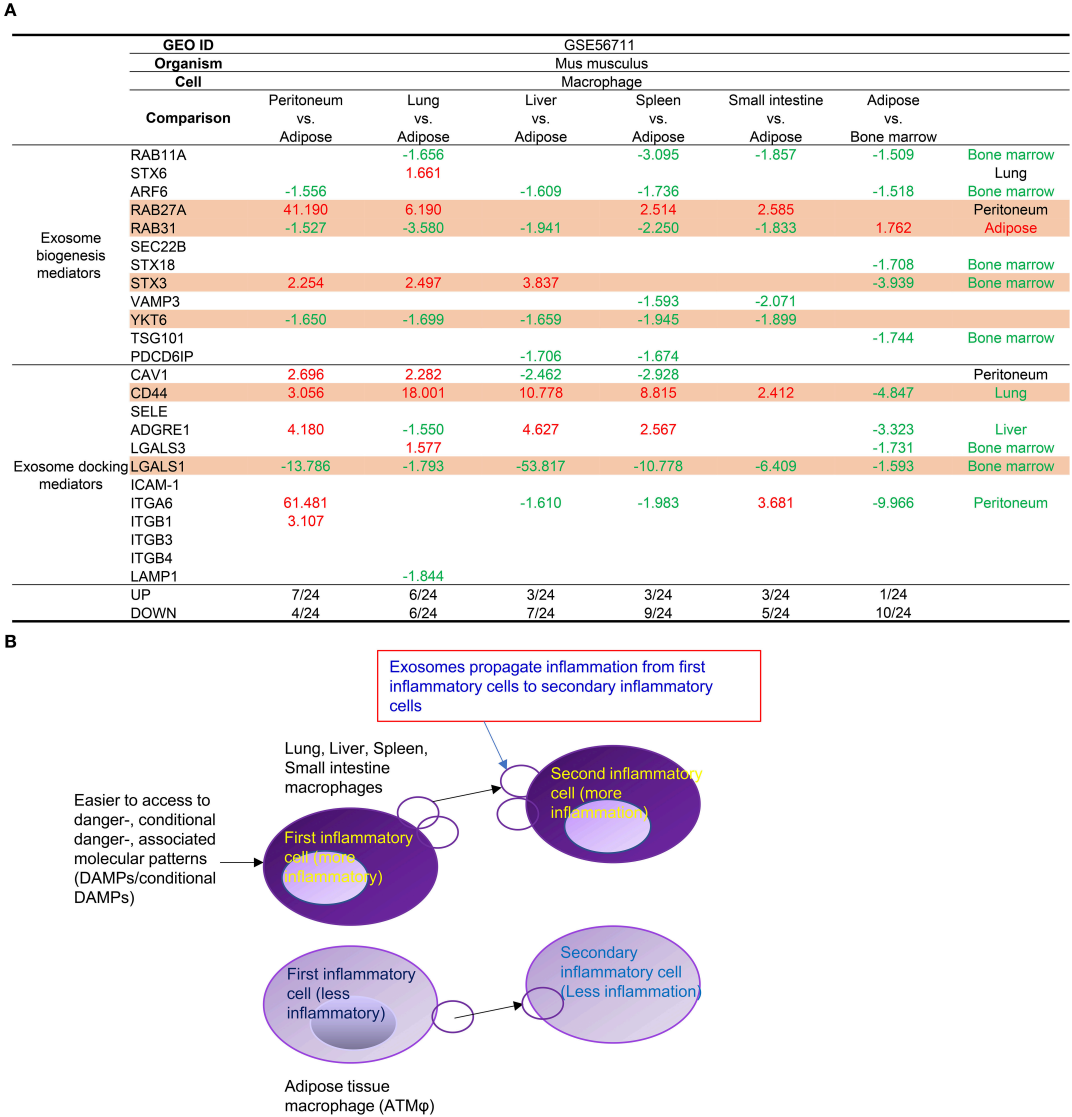
**(A) Mechanism IV**. Tissue macrophages (Mφ) have differences in the expression of several mediators for exosome biogenesis and docking. First, Mφ from peritoneum, lung, liver (STX3), spleen, and small intestine prefer to use RAB27A and STX3 than RAB31 and YKT6 in mediating exosome biogenesis and CD44 for docking in comparison to lean ATMφ. In addition, Mφ from lung also upregulates STX6 (biogenesis), CAV1, and LGALS3 (docking) comparing to lean ATMφ. Moreover, Mφ from peritoneum and intestine upregulate ITGA6 for docking in comparison to lean ATMφ. **(B)** Mφ from peritoneum, lung, liver (STX3), spleen, and small intestine prefer to use RAB27A and STX3 rather than RAB31 and YKT6 in mediating exosome biogenesis and CD44 for docking, presumably to make exosomes more effective in propagating inflammation than adipose tissue macrophages. As cell-cell communication vehicles, exosomes propagate inflammation from first inflammatory cells to secondary inflammatory cells [see also Figure 5 of our previous report for more evidence and the experimental data of others (PMID: 27842563)].

In addition to the above-discussed cell surface mechanisms such as Mφ markers and cell-cell interaction (co-stimulation and co-inhibition/immune checkpoint) receptors, as cell-cell communication mechanisms of Mφ and other cell types, exosomes can transport and deliver a large cargo of proteins, lipids, and nucleic acids and can modify cell and organ function. In addition to their key role as vehicles of intercellular communication, exosomes are increasingly recognized as biomarkers and prognosticators of disease (55). We reported that exosomes might modulate inflammation and immune responses (26) and propagate inflammation (25). We also examined the expression levels of 12 exosome biogenesis mediators and 12 exosome docking mediators in the tissue Mφs (Figure 4A). The results in Figure 4A showed that Mφ from peritoneum, lung, liver, spleen, and small intestine prefer to use RAB27A and syntaxin 3 (STX3) than RAB31 and YKT6 in mediating exosome biogenesis and docking and that adipose tissue Mφs use more Rab31, YKT6, and LGALS1 in mediating exosome biogenesis and docking. Of note, it has been reported that RAB27A-dependent exosome production inhibits chronic inflammation and enables acute response to inflammatory stimuli (56) and that microRNA-30c-2-3p regulates RAB31 and functions as an oncogene in gastric cancer tumorigenesis and development by interacting with glioma-associated oncogene homolog 1(57). The results suggest that the differences in exosome biogenesis and docking in tissue Mφs may be related to their proinflammatory functional status (Figure 4B) as we reported previously (26), that Rab GTPases not only regulate the pathogenesis of cancer and neurodegenerative diseases (58) but may also regulate inflammation functions of Mφ exosomes, and that syntaxin 3-identified homozygous likely deleterious variant (59) may regulate inflammatory Mφ exosomes.

### Levels of Immunometabolism Pathway Enzymes Are Higher in Mφ in Peritoneal, Lung, Liver, Spleen, and Intestine Than in Adipose Tissue Mφ

**Figure 5:**
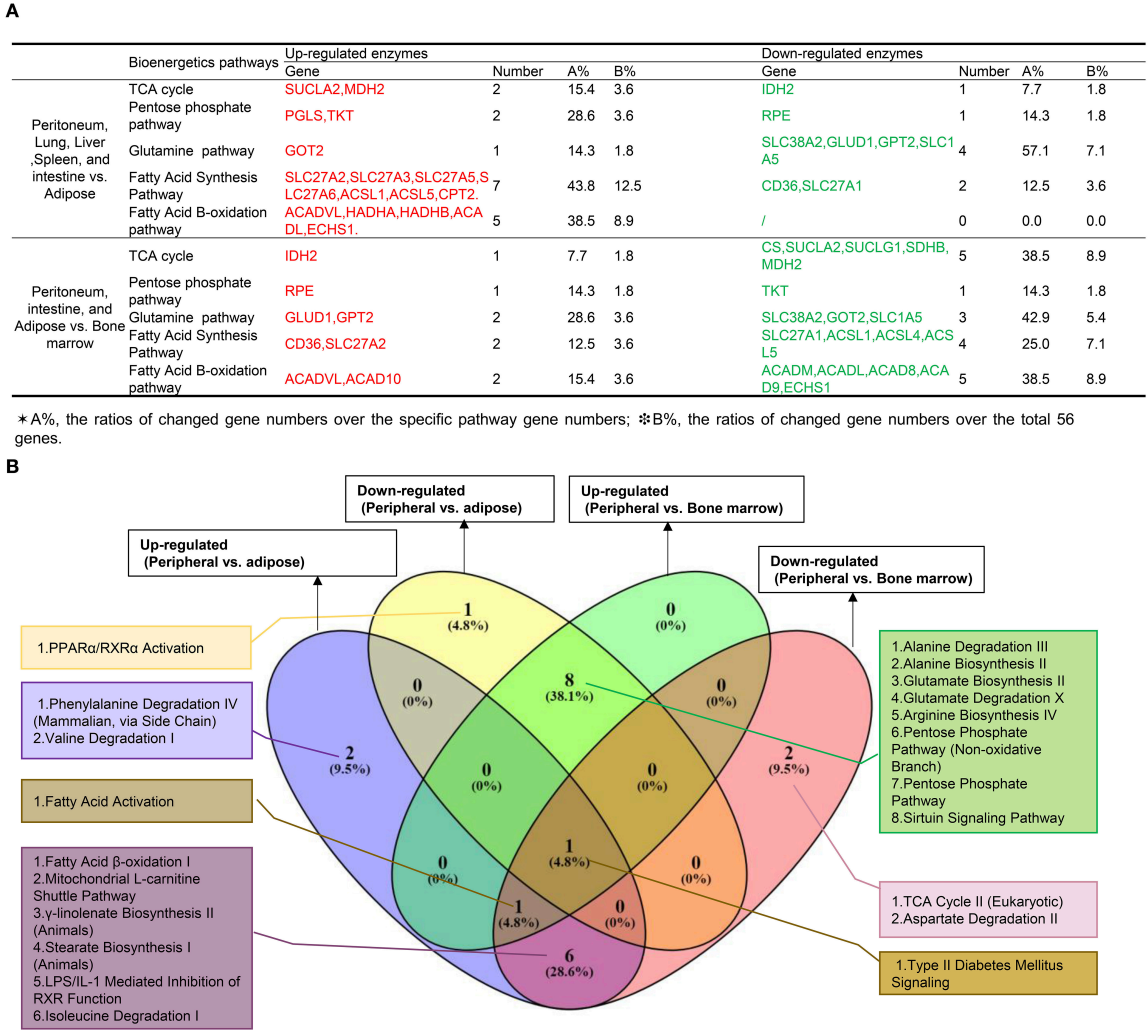
**(A) Mechanism V:** The macrophages from peritoneum, lung, liver, and spleen upregulate more bioenergetics pathway enzymes (immunometabolism pathway) than adipose tissues, with a significantly increased pentose phosphate pathway, three significantly increased fatty acid-related pathways, and a decreased glutamine pathway, and the macrophages from bone marrow express higher bioenergetics pathway enzymes than peritoneum, intestine, and adipose tissues (For detailed expression data, see Figure S7). **(B)** Ingenuity Pathway Analysis of bioenergetics/immunometabolism pathway markers.

Since Mφ bioenergetics metabolism, as an immunometabolism pathway (60), regulates their polarizations (61), we hypothesized that tissue Mφs from different tissues would have various metabolic pathway enzyme genes expressed at different levels. To test this hypothesis, we collected 59 metabolic enzymes involved in six metabolic pathways including the tricarboxylic acid (TCA) cycle (13 enzymes), pentose phosphate pathway (Warburg-Limpam-Dickens cycle and phosphogluconate shunt, 7 enzymes), glutamine pathway (7 enzymes) (62), fatty acid pathway (16 enzymes), fatty acid B-oxidation pathway (63) (13 enzymes), and fatty acid C pathway (9 enzymes), as shown in Table 1. Of note, six genes overlapped in different bioenergetics metabolic pathways. Comparing the Mφ from peritoneal and LLSI tissues with that of adipose tissues (Figure 5A), we found that 2 out of 13 TCA cycle enzymes, 2 out of seven pentose phosphate pathway enzymes, one out of seven glutamine pathway enzymes, 7 out of 16 enzymes in the fatty acid pathway, 5 out of 13 regulators in the fatty acid β-oxidation pathway, and 3 out of 9 fatty acid C pathway enzymes were upregulated. We also found that 1 out of 13 TCA cycle enzymes, 1 out of seven pentose phosphate pathway enzymes, four out of seven glutamine pathway enzymes, and 2 out of 16 enzymes in the fatty acid pathway were downregulated. In addition, comparing the Mφ from peritoneal, intestine, and adipose tissue with that of bone marrow, we found that 1 out of 13 TCA cycle enzymes, 1 out of seven pentose phosphate pathway (PPP) enzymes, 2 out of seven glutamine pathway enzymes, 2 out of 16 enzymes in the fatty acid pathway, and 2 out of 13 regulators in the fatty acid β-oxidation pathway were upregulated and that 5 out of 13 TCA cycle enzymes, 1 out of seven pentose phosphate pathway enzymes, three out of seven glutamine pathway enzymes, 4 out of 16 enzymes in the fatty acid pathway, 5 out of 13 enzymes in the fatty acid β-oxidation pathway and 3 out of 9 fatty acid C pathway enzymes were downregulated. These results suggest that Mφ in peritoneal, lung, liver, spleen, and intestine may upregulate bioenergetics pathway enzyme expression more than in Mφ in adipose tissue Mφ, where expression of the enzymes in the PPP pathway and the three fatty acid pathways increased and expression of glutamine pathway enzymes decreased. Surprisingly, BM-derived Mφ expresses higher bioenergetics pathway enzymes than that of Mφ in peritoneum, intestine, and adipose tissues. As shown in Figure 5B, our Ingenuity Pathway Analysis (IPA) showed that: (1) comparing all the differences among tissue Mφ from peritoneal, lung liver, spleen, intestine, adipose tissue, and bone marrow, type II diabetes signaling is shared; (2) the fatty acid activation pathway is also shared among three groups: (a) upregulated genes in peripheral tissue Mφ vs. ATMφ; (b) upregulated genes in peripheral tissue Mφ vs. BM Mφ; and (c) downregulated genes in peripheral tissue Mφ vs. BM Mφ; and (3) the fatty acid β-oxidation pathway is among the top pathways shared by two groups of upregulated genes in Mφ in peritoneal, lung, liver, spleen, and intestine vs. ATMφ and downregulated genes in Mφ in peritoneal, intestine and ATMφ vs. BM Mφ.

### Expression of Trained Immunity (Innate Immune Memory)-Related Metabolic Genes Is Higher in Mφ From Peritoneum, Lung, Liver, and Spleen Than in ATMφ, and the Expression of Trained Immunity-Related Metabolic Genes Is Higher in Mφ From Peritoneum, Small Intestine and Adipose Tissues Than in Bone Marrow Mφ

**Figure 6:**
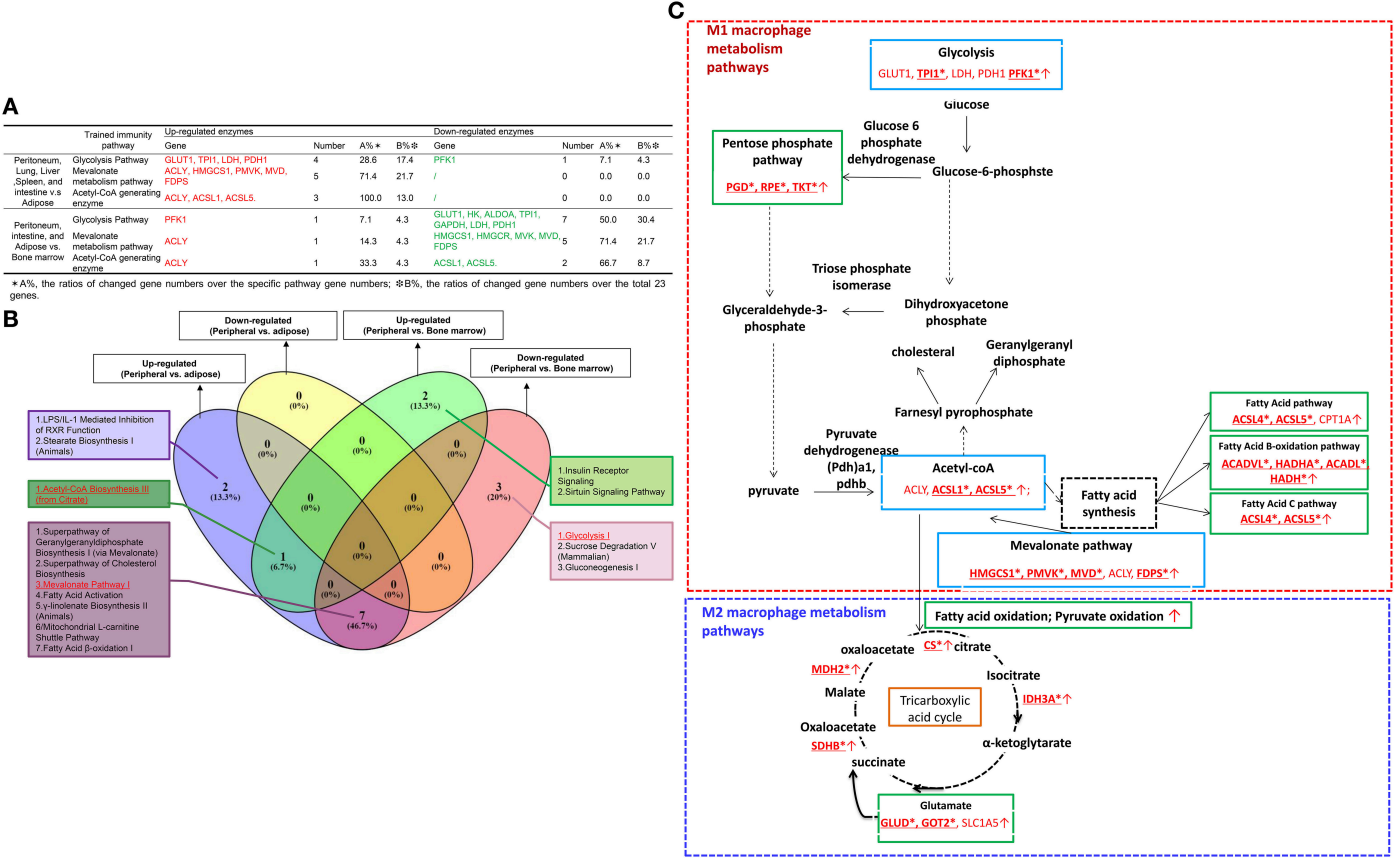
**(A) Mechanism VI:** The macrophages from peritoneum, lung, liver, and spleen upregulate more trained immunity (innate immune memory)-related metabolic genes than that of adipose tissues, and the macrophages from peritoneum, small intestine, and adipose tissues upregulate more trained immunity-related metabolic genes than bone marrow macrophages (For detailed expression data, see Figure S8). **(B)** Ingenuity pathway–Venn Diagram analyses show that the macrophages (Mφ) from peritoneum, lung, liver, spleen, and intestine upregulate more shared trained immunity regulators such as mevalonate pathway regulators than Mφ from bone marrow. **(C)** The expression changes of 19 new enzymes involved in intracellular immunometabolism pathways (IMPs) and trained immunity pathways (TIPs) of M1 Mφ and 6 new enzymes involved in IMPs and TIPs of M2 Mφ may be the mechanisms underlying the higher M1 Mφ proinflammatory status of lung, liver, spleen, and intestine and the disease group-specific pathways and shared disease pathways (PMIDs:28381829; 27396447;26694790; 30679807; 28396078). The green boxes are bioenergetics pathways (Figure 5A), and the blue boxes are trained immunity pathways **(A)**. SLC1A5, GLUT1, LDH, PDH, ACLY, and CPT1A are mentioned in previous studies. However, IDH is found to decrease in M1, but not sure in M2. ^*^25 others (bolded and underlined) are not found in the reviews listed above.

One of the major differences between the adaptive immune system and innate immune systems is that the cells in the adaptive immune system such as T cells have an antigen-specific memory function (64). However, recently it became clear that innate immune cells also have trained immunity (innate immune memory) functions in the form of increases in three key metabolic pathways: glycolysis, acetyl-CoA synthesis, and the mevalonate pathway (65). Thus, in addition to the bioenergetics metabolic pathway analysis, we further examined whether tissue Mφ have differences in the expression of trained immunity pathway enzymes. We hypothesize that the expression of trained immunity pathway enzyme genes in Mφ from peripheral tissues such as lung, liver, spleen, and intestine is higher in than that of ATMφ. As shown in Table 1, we found that 24 enzymes are involved in three pathways of trained immunity functions including 14 enzymes in glycolysis, three enzymes in acetyl-CoA generation, and 7 enzymes in the mevalonate pathway (28, 66). As shown in Figure 6A, Mφ in peritoneal, lung, liver, spleen, and intestine upregulate higher levels of 11 out of 24 enzymes in trained immunity pathways in comparison to ATMφ. In addition, Mφ in peritoneal and intestine and ATMφ upregulate 2 out of 24 enzymes and downregulate 14 out of 24 enzymes in comparison to BM Mφ. The Ingenuity Pathway Analysis showed, as seen in Figure 6B, that the top two pathways involved in upregulation of trained immunity enzymes in Mφ are LPS/IL-1-mediated inhibition of RXR function and stearate biosynthesis I. The top pathway shared by peripheral Mφ and ATMφ upregulation enzymes is acetyl-CoA biosynthesis III. The two pathways among the top three pathways shared by peripheral Mφ and BM Mφ are the superpathway of cholesterol biosynthesis and mevalonate pathway I.

Figure 6C summarizes the findings from Figure 5A (in the green boxes) and Figure 6A (in the blue boxes) into a new map related to the Mφ metabolic pathways identified in M1 and M2. We found that the 19 new enzyme expression changes (with ^*^, bolded, and underlined) involved in immunometabolism pathways and trained immunity pathways may be the mechanisms underlying the higher M1 proinflammatory status of lung, liver, spleen, and intestine. Also, we found that six new enzyme changes in M2-related pathways may also be the mechanisms underlying the higher M2 anti-inflammatory status of adipose tissue Mφ as well as the disease group-specific pathways and shared disease pathways.

## Discussion

Macrophages play a key role in the pathogenesis of various diseases including cardiovascular diseases (13), metabolic diseases (14), infectious diseases (15), respiratory diseases (16), digestive diseases (17), autoimmune diseases (18), and many types of cancers (19, 20). However, it remained unclear whether Mφ use the same pathways and play the same roles or use disease-specific pathways and play disease-specific roles in addition to the shared roles and pathways. To address this question and also to identify the potential mechanisms underlying this issue, we performed a novel type of big—omics database mining analysis, which we pioneered in 2004 (30). We have made the following significant findings. (1) The expression of 31 Mφ markers and 45 Mφ TFs are modulated in eight groups comprising a total of 34 diseases including 10 types of cancers, and both shared and disease-specific pathways for each group of disease/tumor have been identified. To identify the potential mechanisms underlying the Mφ heterogeneity related to disease-group-specific pathways, we examined several novel aspects of Mφ. (2) The expression of M1 Mφ markers is higher in Mφ in lung, liver, spleen, and intestine compared to in lean adipose tissue Mφ in physiological conditions. (3) Pro-adipogenic transcription factors C/EBPα and PPARγ and proinflammatory adipokine leptin upregulate the expression of M1 Mφ markers. Our results correlated well with a recent report implicating a pleiotropic protein prohibitin in regulating adipose-immunometabolism (67). (4) Immunologically peripheral tissue Mφ from lung, liver, spleen, intestine, and bone marrow express higher levels of T cell co-inhibition/immune checkpoint receptor CD274 (programmed death-ligand 1, PDL-1) among ten co-inhibition receptors than that of lean adipose tissues, presumably to counteract the M1 dominant status via its reverse signaling and high homeostatic and anti-inflammatory functions (52). Our results reveal a new mechanism underlying the toxicities of the anti-PD-1 and anti-PD-L1 immune checkpoint antibodies (68). (5) Tissue Mφ from lung, liver, spleen, intestine, and bone marrow prefer to use RAB27A and STX3 than RAB31 and YKT6 in mediating exosome biogenesis and docking, suggesting new inflammatory exosome markers and a new inflammatory exosome status for propagating inflammation from inflamed cells to secondary inflammatory cells as we reported previously (25). Our results correlated well with recent findings that inflammation leads to distinct populations of extracellular vesicles (69). (6) To address why Mφ in peripheral tissues have a higher M1 status than those in adipose tissues, we found that Mφ in peritoneal, lung, liver, spleen, and intestine upregulate higher levels of immunometabolism pathway enzymes than adipose tissue Mφ (ATMφ). (7) To address the potential mechanism underlying the higher M1 proinflammatory status of Mφ in peripheral tissues, we found that Mφ from peritoneum, lung, liver, and spleen upregulate more trained immunity (innate immune memory)-related metabolic genes than that of adipose tissues and that the macrophages from peritoneum, small intestine, and adipose tissues upregulate more trained immunity-related metabolic genes than bone marrow macrophages. Taken together, our results suggest that multiple mechanisms such as those at the cell surface including M1 Mφ markers, cell-cell contact receptors, cell-cell communication exosomes, intracellular immunometabolism and trained immunity, and Mφ nuclear transcription factors may be responsible for the disease group-specific pathways and shared pathways that we found in eight groups of 34 diseases.

Since CD274 reverse signaling works via its interaction with CD279 (PD1) expressed in CD4+ T cells, CD8+ T cells, B cells, macrophages, and dendritic cells (70), these analyses emphasize the following. (1) CD279+ T cells (both CD4+ and CD8+) and B cells have significant innate immune functions in controlling CD274+ Mφ proinflammatory status and maintaining tissue homeostasis in addition to having antigen-specific adaptive immune functions. (2) T cell co-stimulation and co-inhibition receptors serve as prototypic cell surface receptor-mediated cell-cell contact signaling in addition to classical signaling pathways from cytokine receptors, growth factor receptors, pathogen-associated molecular pattern receptors (PAMP-Rs), danger-associated molecular pattern receptors (DAMP-Rs), and conditional DAMP-Rs, as we reported previously (12). (3) Our results suggest a potential molecular mechanism underlying the clinical finding that elevated immune-related adverse effects (irAEs) of systematically injected anti-PD-L1 monoclonal antibody (mAb) (Durvalumab) in patients with cancers (71). Blocking CD274 reverse signaling could activate all the CD274+ tissue macrophages and contribute to elevated immune-related adverse effects (irAEs). (4) Since CD279 is also expressed in tumor-associated macrophages (TAMs) (72), our results also suggest a possibility that CD279-CD274 interaction on TAMs may suppress the anti-tumor functions of TAMs via reverse signaling.

**Figure 7:**
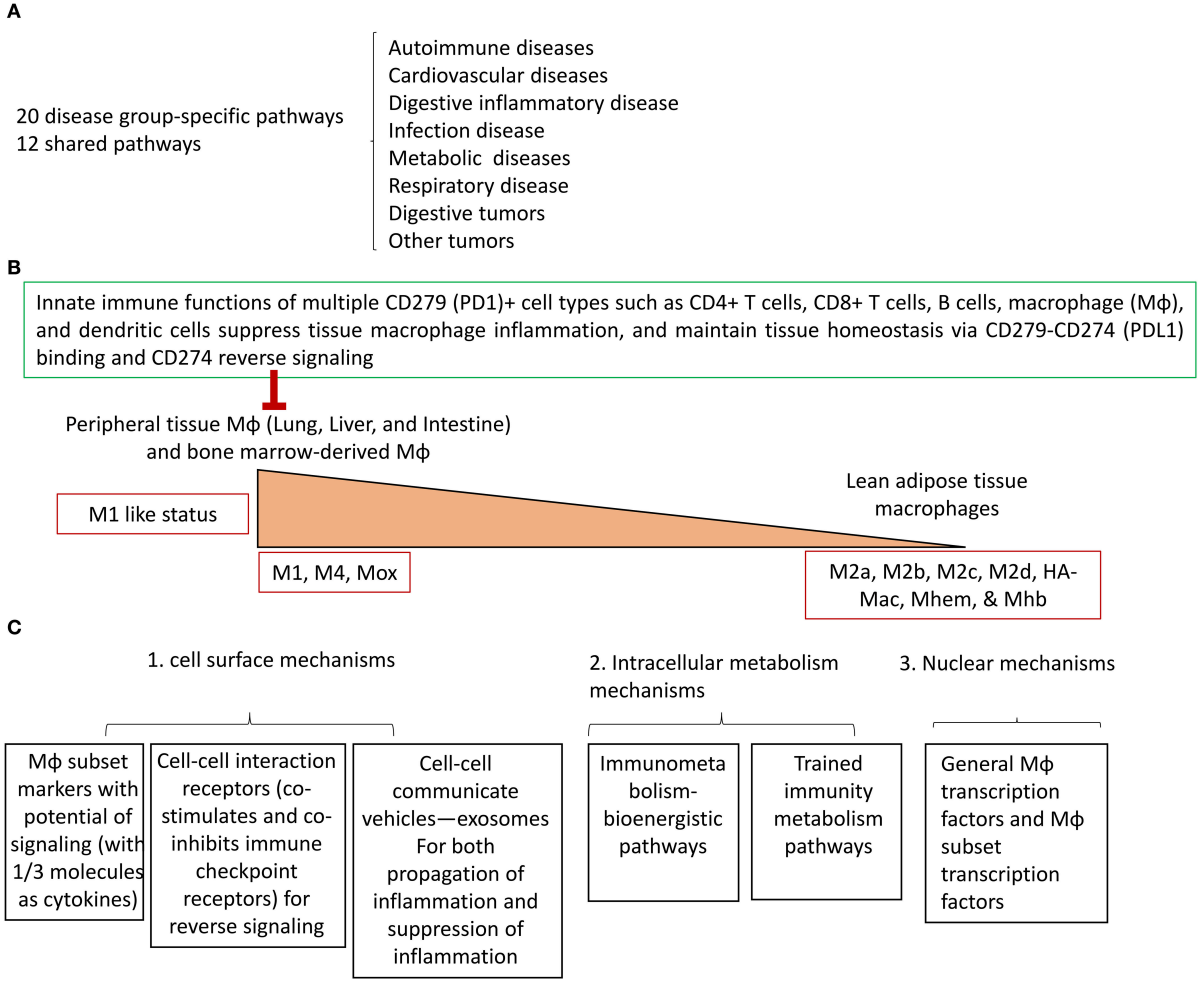
A new working model. **(A)** Twenty novel disease group-specific-, and 12 new shared- macrophage pathways have been identified in eight groups of 34 diseases including 24 inflammatory organ diseases and 10 types of tumors as the phenotypic findings. **(B)** To identify potential mechanisms underlying the macrophage phenotypes, we identified new tissue mechanisms that macrophages in peripheral tissues have higher M1 like pro-inflammatory status than lead adipose tissue macrophages, which are controlled by high expression of the immune checkpoint/co-inhibition receptor CD274 via its reverse signaling. **(C)** We identified six new cell and molecular mechanisms including three cell surface mechanisms, two groups of intracellular metabolism pathways and two groups of nuclear transcription factors.

To summarize our findings, we have proposed a new working model with three connected parts. Figure 7A illustrates the first part: based on the expression levels of two groups of 31 Mφ subset markers (9) and 45 Mφ transcription factors in the eight groups of 34 diseases (also see Table 2), we have identified for the first time 20 novel Mφ disease group-specific pathways and 12 disease-shared pathways (shared in more than four major disease groups). These results have demonstrated two aspects for the first time. First, the pathogenesis of various diseases and tumors significantly modulates Mφ signaling pathways in disease group-specific and shared manners. Figure 7B illustrates the second: the potential tissue mechanisms underlying the above-mentioned Mφ heterogeneity in diseased conditions. Based on the differential expression of regulators including M1 markers, M1 TFs, co-stimulation and co-inhibition/immune checkpoint receptors, cell-cell communication exosome biogenesis machinery, M1 bioenergetic enzymes, and trained immunity enzymes, we proposed a new concept of tissue M1 Mφ status. We found that first, Mφs in liver, small intestine, and bone marrow-derived Mφ have the highest macrophage inflammation potential and, second, adipose tissue from lean animals and surprisingly spleen have low Mφ inflammation potential. Of note, white adipose tissue hypertrophy recruits significant numbers of inflammatory cells including Mφ (73). The new data have demonstrated that various tissue Mφ have significant differences in M1 proinflammatory status, which could be controlled by high expression of a co-inhibition receptor such as CD274 (PDL1)-initiated anti-inflammatory reverse signaling. It is noteworthy that each tissue has its own composition of embryonically derived and adult-derived Mφ, but it is unclear whether the Mφ of distinct origins are functionally interchangeable or have unique roles at steady state (74). These issues can be examined in the future when new microarray/RNA-sequencing (RNA-Seq) data are available. Figure 7C illustrates the third part of the model: three novel major cell/molecular mechanisms underlying the above-mentioned Mφ heterogeneity in diseased conditions. The six novel cell and molecular mechanisms include three cell surface mechanisms (Mφ subset markers with potential of signaling, cell-cell interaction receptors (co-stimulation and co-inhibition/immune checkpoint receptors), and inflammation-modulating cell-cell communication exosomes), two new metabolism mechanisms (the immunometabolism/bioenergistic and trained immunity metabolic pathways), and, finally, nuclear transcription factors. Taken together, the new tissue, cell, and molecular mechanisms may contribute to the novel Mφ signaling heterogeneity in diseased conditions that we have found.

We acknowledge that carefully designed *in-vitro* and *in-vivo* experimental models will be needed to verify all the results we report here. These experimental models will enable the consolidation of the Mφ disease group-specific pathways in various pathological conditions. However, the big data mining analyses that we pioneered in 2004 (30) have provided significant insights into the Mφ disease group-specific and shared pathways and heterogeneity, homeostasis, and functions of Mφ in various diseases and cancers/tumors and have also identified novel therapeutic targets for treating cancers/tumors and inflammation, tissue regeneration, and tissue repair.

## Materials and Methods

### Expression Profile of Mφ Subset Markers, Exosome Biogenesis Mediators, Exosome Docking Mediators, Bioenergic Pathway Enzymes, T Cell Co-stimulation and Co-inhibition Receptors, and Mφ Transcription Factors in Mφs

Microarray datasets were collected from the National Institutes of Health (NIH)-National Center for Biotechnology Information (NCBI) GEO DataSets (https://www.ncbi.nlm.nih.gov/gds/) databases and analyzed with GEO2R (https://www.ncbi.nlm.nih.gov/geo/geo2r/). The numbers of 11 GEO datasets in non-diseased conditions are as follows: GSE56711, GSE85346, GSE55760, GSE59585, GSE14004, GSE37514, GSE50183, GSE66073, GSE46320, GSE27017, and GSE56711. The numbers of 32 GEO datasets in diseased conditions are as follows: GSE55235, GSE81622, GSE27335, GSE57376, GSE46451, GSE27411, GSE16879, GSE29507, GSE48080, GSE65517, GSE40224, GSE19339, GSE23561, GSE57691, GSE23561, GSE6088, GSE55100, GSE25724, GSE65204, GSE37768, GSE53408, GSE48080, GSE45670, GSE79973, GSE74656, GSE41657, GSE16515, GSE75037, GSE70951, GSE46602, GSE36668, and GSE75038. The number of the GEO dataset in gene knock-out mice is as follows: GSE40493.

As shown in Figure 1, 207 regulator genes in seven groups were studied in this paper, including 31 Mφ subset marker genes, 18 Mφ subset transcription factor genes (TF), 27 Mφ general transcription factor genes (21), 28 T cell co-stimulation and co-inhibition receptor genes, bioenergetics pathway enzymes genes and trained immunity pathway gene numbers are totally 80. The logic flow and rationale are explained in Figure 1 and Table 2. We also analyzed the expression of four house-keeping genes for all of the GEO datasets used. The house-keeping gene list was extracted from a related report (74).

Genes with a more than 1.5-fold expression change were defined as the upregulated genes, while genes with an expression change of less than 1.5-fold were defined as downregulated genes.

### Ingenuity Pathway Analysis

We utilized Ingenuity Pathway Analysis (IPA, Ingenuity Systems, http://pages.ingenuity.com/rs/ingenuity/images/IPA_data_sheet.pdf) to characterize clinical relevance and molecular and cellular functions related to the genes identified in our microarray analysis. The differentially expressed genes were identified and uploaded into IPA for analysis. The core and pathways analysis was used to identify molecular and cellular pathways, as we reported previously (25, 75, 76).

## Data Availability Statement

Publicly available datasets were analyzed in this study. This data can be found here: https://4dgenome.research.chop.edu, https://www.ncbi.nlm.nih.gov/gds/.

## Author Contributions

BL and JW carried out the data gathering and data analysis and prepared tables and figures. AF, YSu, JSa, YL, GN, WY, DY, YSh, CD, CJ, FS, RZ, QY, KX, KM, RC, HF, SW, LS, PZ, XQ, JY, DF, YHS, JSu, TR, EC, and HW aided with analysis of the data. XY supervised the experimental design, data analysis, and manuscript writing. All authors read and approved the final manuscript.

### Conflict of Interest

The authors declare that the research was conducted in the absence of any commercial or financial relationships that could be construed as a potential conflict of interest.

## Abbreviations

TFs: transcription factors
LLSI: lung, liver, spleen, and intestine
Mφ: Macrophages
DAMPs: danger associated molecular patterns
IL-1β: interleukin-1β
ATMφ: adipose tissues Mφ
BM: bone marrow
Treg: regulatory T cell
APC: antigen presenting cell
STAT1: signal transducer and activator of transcription 1
NCBI: National Center for Biotechnology Information
LPS: lipopolysaccharide
IFN-γ: interferon- γ
IL-4: interleukin-4
TGF-β: transforming growth factor-β
IL-1Ra: IL-1 receptor antagonist
MHO: metabolically healthy obese
KLF: Krüppel-like family
C/EBPα: CCAAT/enhancer-binding protein α
PPARγ: peroxisome proliferator-activated receptor γ
CXCL10: C-X-C motif chemokine 10
STAB1: stabilin 1
F13A1: coagulation factor XIII A chain
Chil4: chitinase-like 4
ARG1: arginase 1
SFRP5: secreted frizzled-related protein 5
PDL1: programmed death-ligand 1
FoxO: Forkhead box O
PD-L1: programmed death ligand 1
PPP: pentose phosphate pathway
IPA: Ingenuity Pathway Analysis
STX3: syntaxin 3
VAT: visceral adipose tissue
IRF4: interferon regulatory factor 4
MARE: Maf recognition elements
Tfh: follicular T helper cell
PAMP-Rs: pathogen associated molecular pattern receptors
DAMP-Rs: danger associated molecular pattern receptors
irAEs: immune-related adverse effects
mAb: monoclonal antibody
TAMs: tumor associated macrophages.
