# Supplementary material for: Twenty Novel Disease Group-Specific and 12 New Shared Macrophage Pathways in Eight Groups of 34 Diseases Including 24 Inflammatory Organ Diseases and 10 Types of Tumors
Source: Front Immunol. 2019 Nov 14;10:2612. doi: 10.3389/fimmu.2019.02612 (PMC6880770; doi:10.3389/fimmu.2019.02612)
Supplement: Supplementary file 1 [file Presentation_1.PPTX]

## Slide 1
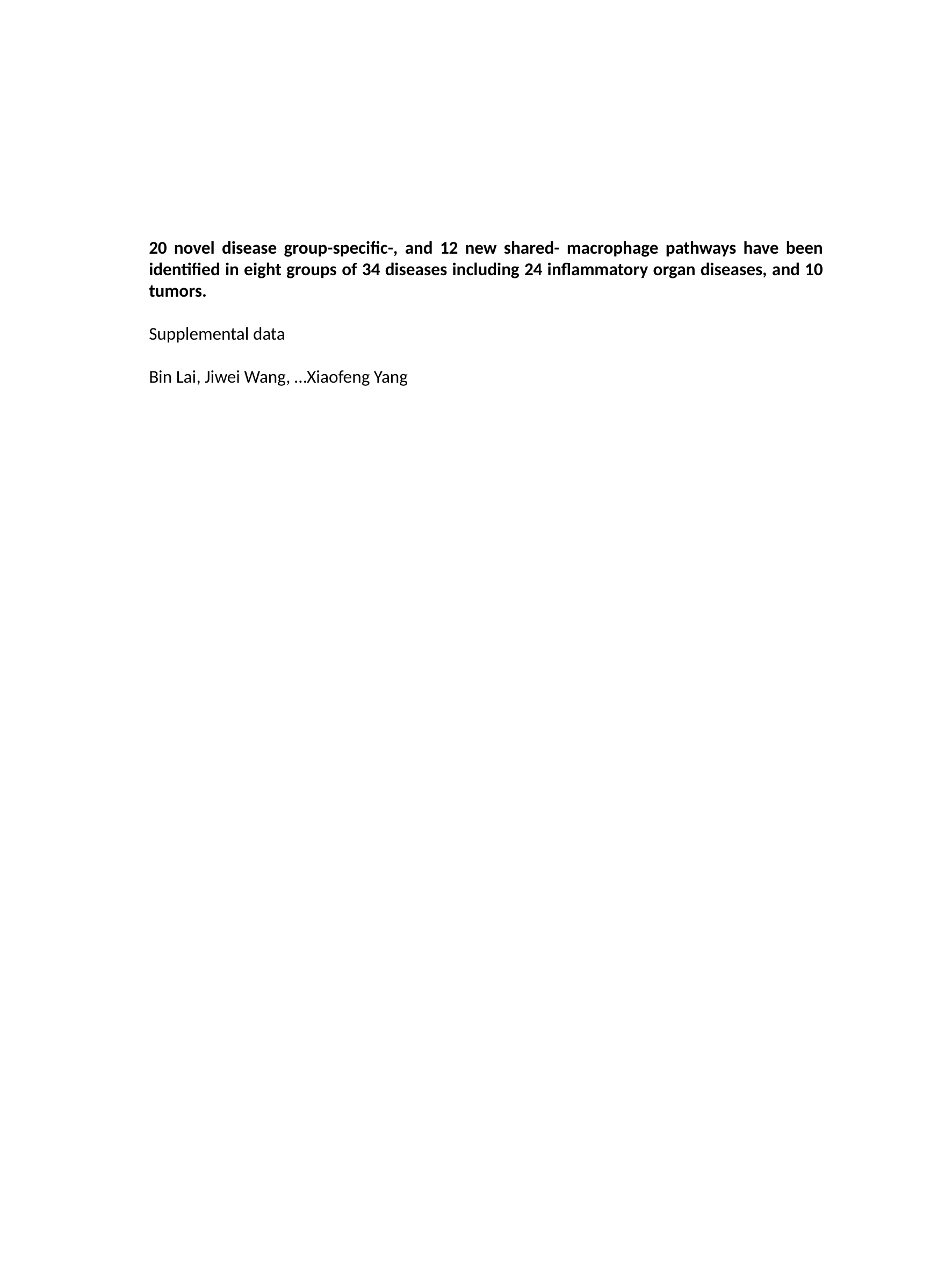

20 novel disease group-specific-, and 12 new shared- macrophage pathways have been identified in eight groups of 34 diseases including 24 inflammatory organ diseases, and 10 tumors.
Supplemental data
Bin Lai, Jiwei Wang, …Xiaofeng Yang

## Slide 2
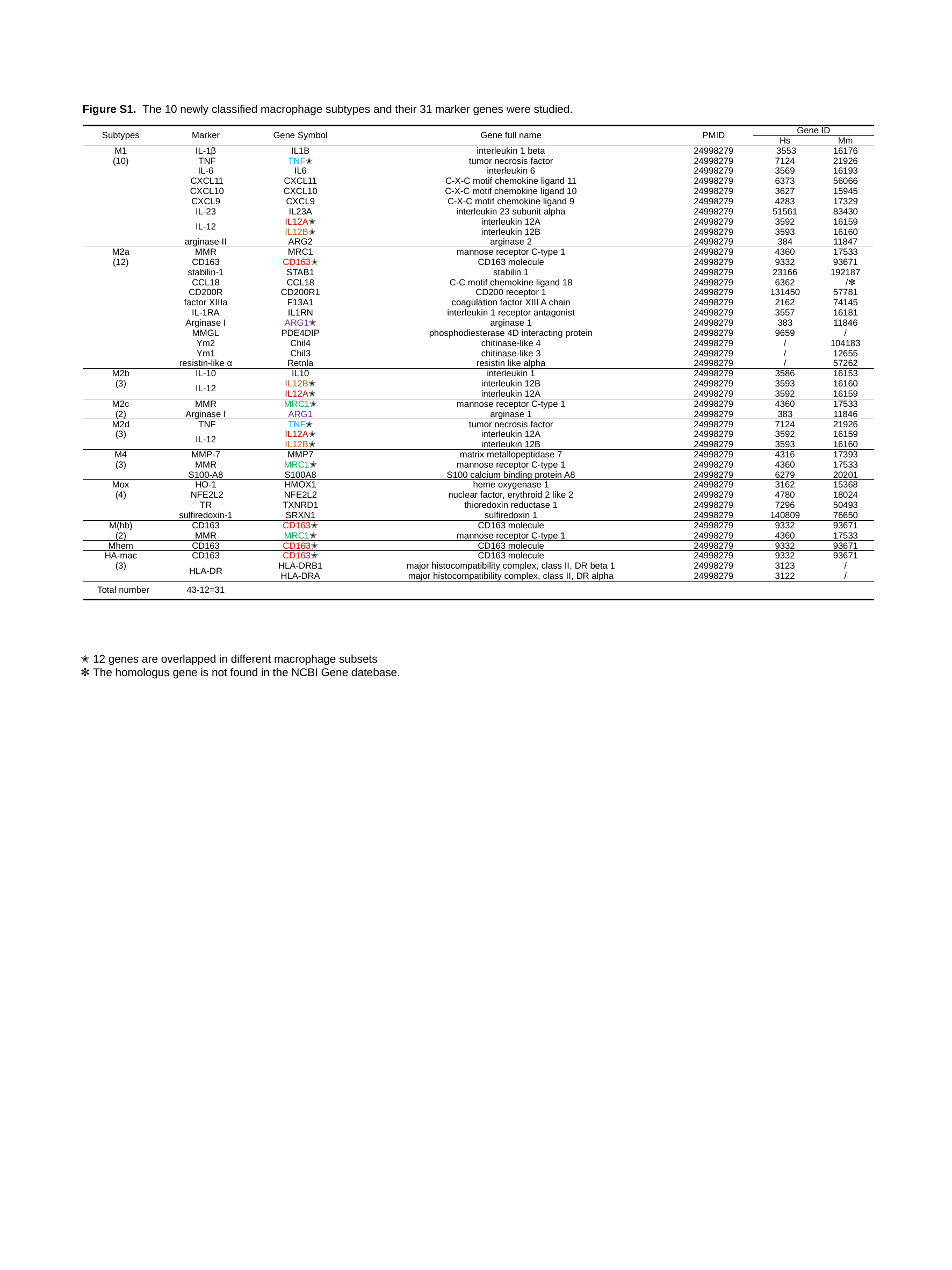

Figure S1. The 10 newly classified macrophage subtypes and their 31 marker genes were studied.
| Subtypes | Marker | Gene Symbol | Gene full name | PMID | Gene ID | |
| --- | --- | --- | --- | --- | --- | --- |
| | | | | | Hs | Mm |
| M1 | IL‑1β | IL1B | interleukin 1 beta | 24998279 | 3553 | 16176 |
| (10) | TNF | TNF✭ | tumor necrosis factor | 24998279 | 7124 | 21926 |
| | IL‑6 | IL6 | interleukin 6 | 24998279 | 3569 | 16193 |
| | CXCL11 | CXCL11 | C-X-C motif chemokine ligand 11 | 24998279 | 6373 | 56066 |
| | CXCL10 | CXCL10 | C-X-C motif chemokine ligand 10 | 24998279 | 3627 | 15945 |
| | CXCL9 | CXCL9 | C-X-C motif chemokine ligand 9 | 24998279 | 4283 | 17329 |
| | IL‑23 | IL23A | interleukin 23 subunit alpha | 24998279 | 51561 | 83430 |
| | IL‑12 | IL12A✭ | interleukin 12A | 24998279 | 3592 | 16159 |
| | | IL12B✭ | interleukin 12B | 24998279 | 3593 | 16160 |
| | arginase II | ARG2 | arginase 2 | 24998279 | 384 | 11847 |
| M2a | MMR | MRC1 | mannose receptor C-type 1 | 24998279 | 4360 | 17533 |
| (12) | CD163 | CD163✭ | CD163 molecule | 24998279 | 9332 | 93671 |
| | stabilin‑1 | STAB1 | stabilin 1 | 24998279 | 23166 | 192187 |
| | CCL18 | CCL18 | C-C motif chemokine ligand 18 | 24998279 | 6362 | /✼ |
| | CD200R | CD200R1 | CD200 receptor 1 | 24998279 | 131450 | 57781 |
| | factor XIIIa | F13A1 | coagulation factor XIII A chain | 24998279 | 2162 | 74145 |
| | IL‑1RA | IL1RN | interleukin 1 receptor antagonist | 24998279 | 3557 | 16181 |
| | Arginase I | ARG1✭ | arginase 1 | 24998279 | 383 | 11846 |
| | MMGL | PDE4DIP | phosphodiesterase 4D interacting protein | 24998279 | 9659 | / |
| | Ym2 | Chil4 | chitinase-like 4 | 24998279 | / | 104183 |
| | Ym1 | Chil3 | chitinase-like 3 | 24998279 | / | 12655 |
| | resistin-like α | Retnla | resistin like alpha | 24998279 | / | 57262 |
| M2b | IL‑10 | IL10 | interleukin 1 | 24998279 | 3586 | 16153 |
| (3) | IL‑12 | IL12B✭ | interleukin 12B | 24998279 | 3593 | 16160 |
| | | IL12A✭ | interleukin 12A | 24998279 | 3592 | 16159 |
| M2c | MMR | MRC1✭ | mannose receptor C-type 1 | 24998279 | 4360 | 17533 |
| (2) | Arginase I | ARG1 | arginase 1 | 24998279 | 383 | 11846 |
| M2d | TNF | TNF✭ | tumor necrosis factor | 24998279 | 7124 | 21926 |
| (3) | IL-12 | IL12A✭ | interleukin 12A | 24998279 | 3592 | 16159 |
| | | IL12B✭ | interleukin 12B | 24998279 | 3593 | 16160 |
| M4 | MMP‑7 | MMP7 | matrix metallopeptidase 7 | 24998279 | 4316 | 17393 |
| (3) | MMR | MRC1✭ | mannose receptor C-type 1 | 24998279 | 4360 | 17533 |
| | S100‑A8 | S100A8 | S100 calcium binding protein A8 | 24998279 | 6279 | 20201 |
| Mox | HO-1 | HMOX1 | heme oxygenase 1 | 24998279 | 3162 | 15368 |
| (4) | NFE2L2 | NFE2L2 | nuclear factor, erythroid 2 like 2 | 24998279 | 4780 | 18024 |
| | TR | TXNRD1 | thioredoxin reductase 1 | 24998279 | 7296 | 50493 |
| | sulfiredoxin-1 | SRXN1 | sulfiredoxin 1 | 24998279 | 140809 | 76650 |
| M(hb) | CD163 | CD163✭ | CD163 molecule | 24998279 | 9332 | 93671 |
| (2) | MMR | MRC1✭ | mannose receptor C-type 1 | 24998279 | 4360 | 17533 |
| Mhem | CD163 | CD163✭ | CD163 molecule | 24998279 | 9332 | 93671 |
| HA-mac | CD163 | CD163✭ | CD163 molecule | 24998279 | 9332 | 93671 |
| (3) | HLA-DR | HLA-DRB1 | major histocompatibility complex, class II, DR beta 1 | 24998279 | 3123 | / |
| | | HLA-DRA | major histocompatibility complex, class II, DR alpha | 24998279 | 3122 | / |
| Total number | 43-12=31 | | | | | |
✭ 12 genes are overlapped in different macrophage subsets
✼ The homologus gene is not found in the NCBI Gene datebase.

## Slide 3
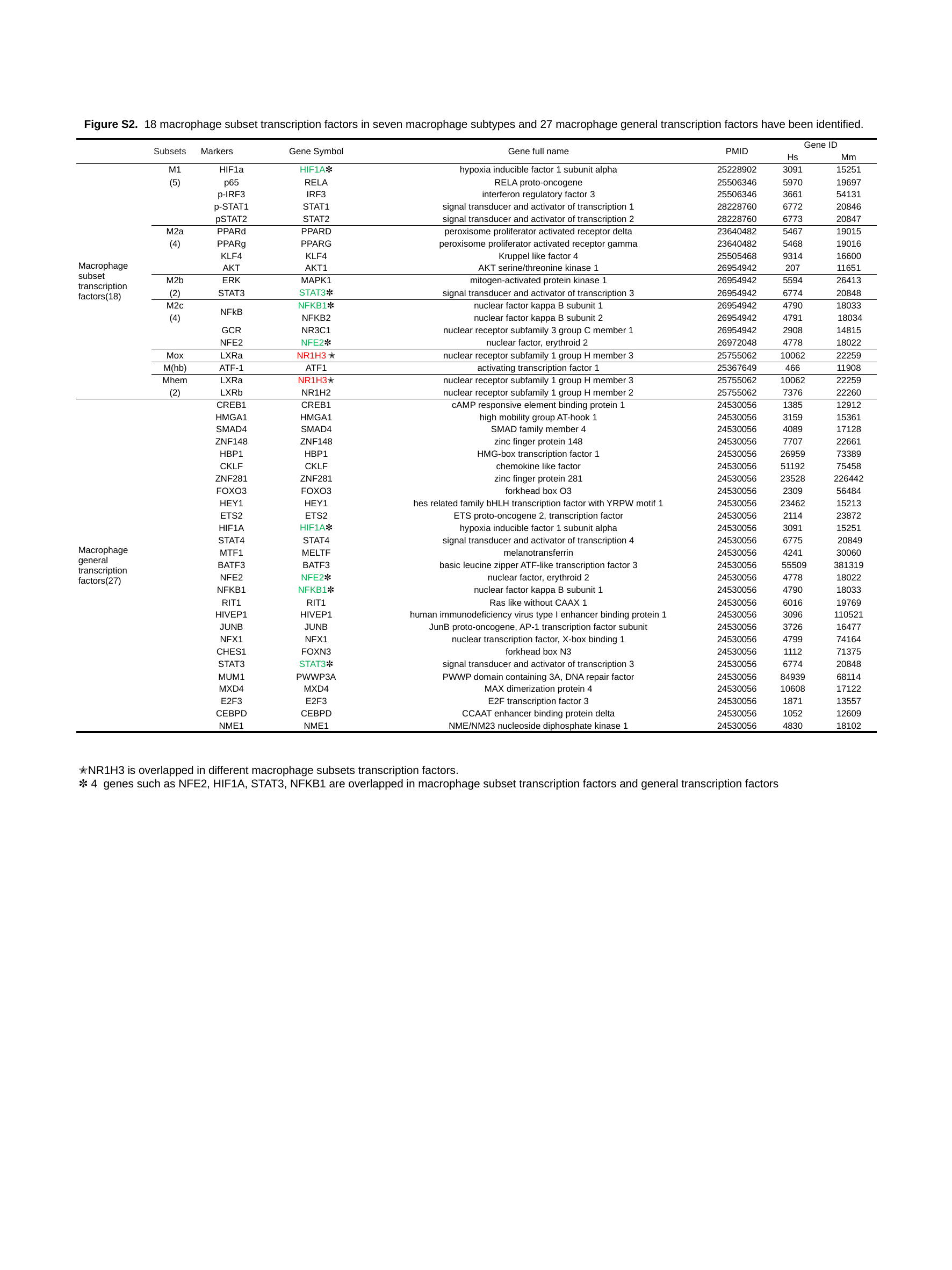

# Figure S2. 18 macrophage subset transcription factors in seven macrophage subtypes and 27 macrophage general transcription factors have been identified.
| | Subsets | Markers | Gene Symbol | Gene full name | PMID | Gene ID | |
| --- | --- | --- | --- | --- | --- | --- | --- |
| | | | | | | Hs | Mm |
| Macrophage subset transcription factors(18) | M1 | HIF1a | HIF1A✼ | hypoxia inducible factor 1 subunit alpha | 25228902 | 3091 | 15251 |
| | (5) | p65 | RELA | RELA proto-oncogene | 25506346 | 5970 | 19697 |
| | | p-IRF3 | IRF3 | interferon regulatory factor 3 | 25506346 | 3661 | 54131 |
| | | p-STAT1 | STAT1 | signal transducer and activator of transcription 1 | 28228760 | 6772 | 20846 |
| | | pSTAT2 | STAT2 | signal transducer and activator of transcription 2 | 28228760 | 6773 | 20847 |
| | M2a | PPARd | PPARD | peroxisome proliferator activated receptor delta | 23640482 | 5467 | 19015 |
| | (4) | PPARg | PPARG | peroxisome proliferator activated receptor gamma | 23640482 | 5468 | 19016 |
| | | KLF4 | KLF4 | Kruppel like factor 4 | 25505468 | 9314 | 16600 |
| | | AKT | AKT1 | AKT serine/threonine kinase 1 | 26954942 | 207 | 11651 |
| | M2b | ERK | MAPK1 | mitogen-activated protein kinase 1 | 26954942 | 5594 | 26413 |
| | (2) | STAT3 | STAT3✼ | signal transducer and activator of transcription 3 | 26954942 | 6774 | 20848 |
| | M2c | NFkB | NFKB1✼ | nuclear factor kappa B subunit 1 | 26954942 | 4790 | 18033 |
| | (4) | | NFKB2 | nuclear factor kappa B subunit 2 | 26954942 | 4791 | 18034 |
| | | GCR | NR3C1 | nuclear receptor subfamily 3 group C member 1 | 26954942 | 2908 | 14815 |
| | | NFE2 | NFE2✼ | nuclear factor, erythroid 2 | 26972048 | 4778 | 18022 |
| | Mox | LXRa | NR1H3 ✭ | nuclear receptor subfamily 1 group H member 3 | 25755062 | 10062 | 22259 |
| | M(hb) | ATF-1 | ATF1 | activating transcription factor 1 | 25367649 | 466 | 11908 |
| | Mhem | LXRa | NR1H3✭ | nuclear receptor subfamily 1 group H member 3 | 25755062 | 10062 | 22259 |
| | (2) | LXRb | NR1H2 | nuclear receptor subfamily 1 group H member 2 | 25755062 | 7376 | 22260 |
| Macrophage general transcription factors(27) | | CREB1 | CREB1 | cAMP responsive element binding protein 1 | 24530056 | 1385 | 12912 |
| | | HMGA1 | HMGA1 | high mobility group AT-hook 1 | 24530056 | 3159 | 15361 |
| | | SMAD4 | SMAD4 | SMAD family member 4 | 24530056 | 4089 | 17128 |
| | | ZNF148 | ZNF148 | zinc finger protein 148 | 24530056 | 7707 | 22661 |
| | | HBP1 | HBP1 | HMG-box transcription factor 1 | 24530056 | 26959 | 73389 |
| | | CKLF | CKLF | chemokine like factor | 24530056 | 51192 | 75458 |
| | | ZNF281 | ZNF281 | zinc finger protein 281 | 24530056 | 23528 | 226442 |
| | | FOXO3 | FOXO3 | forkhead box O3 | 24530056 | 2309 | 56484 |
| | | HEY1 | HEY1 | hes related family bHLH transcription factor with YRPW motif 1 | 24530056 | 23462 | 15213 |
| | | ETS2 | ETS2 | ETS proto-oncogene 2, transcription factor | 24530056 | 2114 | 23872 |
| | | HIF1A | HIF1A✼ | hypoxia inducible factor 1 subunit alpha | 24530056 | 3091 | 15251 |
| | | STAT4 | STAT4 | signal transducer and activator of transcription 4 | 24530056 | 6775 | 20849 |
| | | MTF1 | MELTF | melanotransferrin | 24530056 | 4241 | 30060 |
| | | BATF3 | BATF3 | basic leucine zipper ATF-like transcription factor 3 | 24530056 | 55509 | 381319 |
| | | NFE2 | NFE2✼ | nuclear factor, erythroid 2 | 24530056 | 4778 | 18022 |
| | | NFKB1 | NFKB1✼ | nuclear factor kappa B subunit 1 | 24530056 | 4790 | 18033 |
| | | RIT1 | RIT1 | Ras like without CAAX 1 | 24530056 | 6016 | 19769 |
| | | HIVEP1 | HIVEP1 | human immunodeficiency virus type I enhancer binding protein 1 | 24530056 | 3096 | 110521 |
| | | JUNB | JUNB | JunB proto-oncogene, AP-1 transcription factor subunit | 24530056 | 3726 | 16477 |
| | | NFX1 | NFX1 | nuclear transcription factor, X-box binding 1 | 24530056 | 4799 | 74164 |
| | | CHES1 | FOXN3 | forkhead box N3 | 24530056 | 1112 | 71375 |
| | | STAT3 | STAT3✼ | signal transducer and activator of transcription 3 | 24530056 | 6774 | 20848 |
| | | MUM1 | PWWP3A | PWWP domain containing 3A, DNA repair factor | 24530056 | 84939 | 68114 |
| | | MXD4 | MXD4 | MAX dimerization protein 4 | 24530056 | 10608 | 17122 |
| | | E2F3 | E2F3 | E2F transcription factor 3 | 24530056 | 1871 | 13557 |
| | | CEBPD | CEBPD | CCAAT enhancer binding protein delta | 24530056 | 1052 | 12609 |
| | | NME1 | NME1 | NME/NM23 nucleoside diphosphate kinase 1 | 24530056 | 4830 | 18102 |
 ✭NR1H3 is overlapped in different macrophage subsets transcription factors.
 ✼ 4 genes such as NFE2, HIF1A, STAT3, NFKB1 are overlapped in macrophage subset transcription factors and general transcription factors

## Slide 4
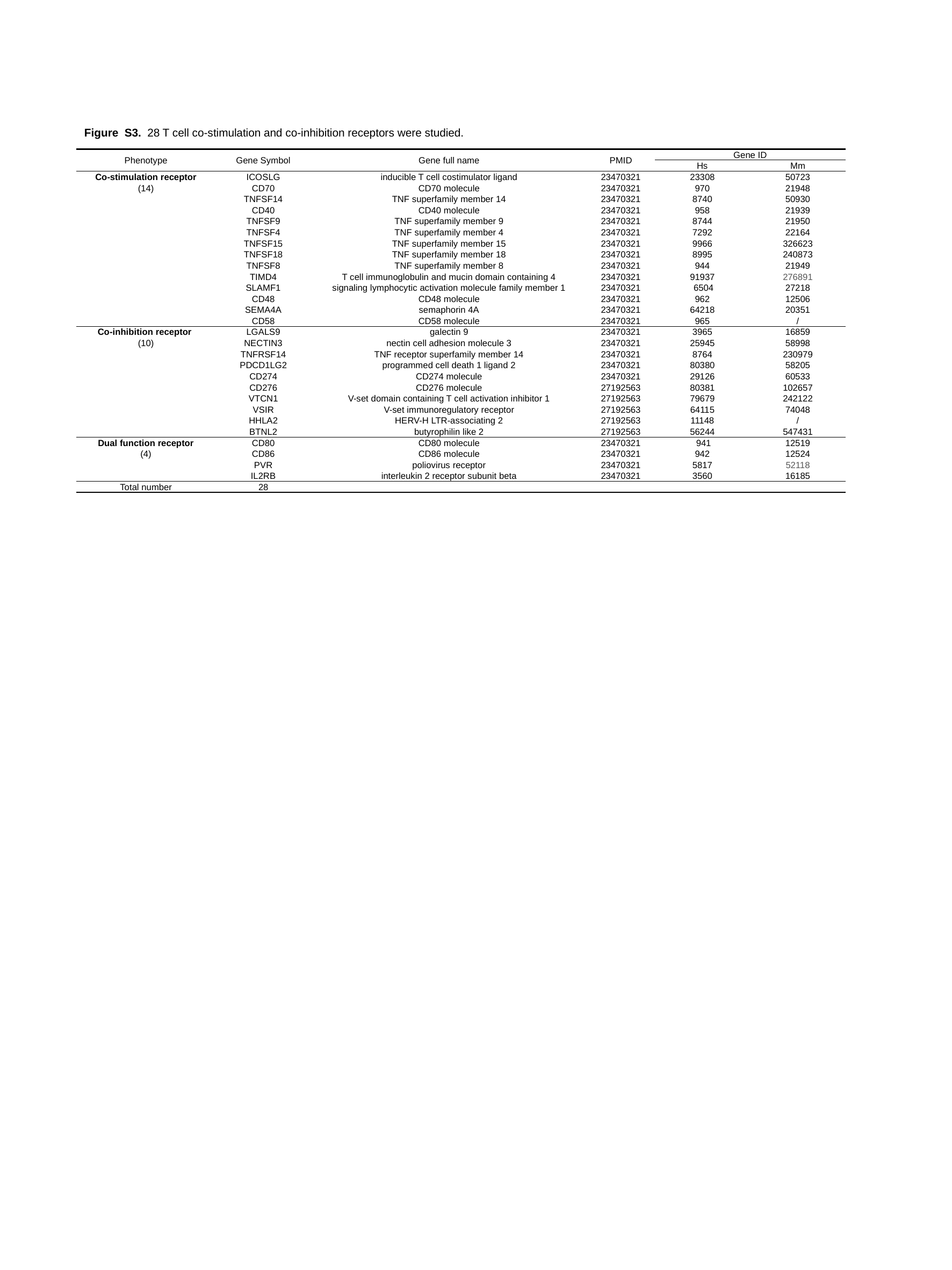

# Figure S3. 28 T cell co-stimulation and co-inhibition receptors were studied.
| Phenotype | Gene Symbol | Gene full name | PMID | Gene ID | |
| --- | --- | --- | --- | --- | --- |
| | | | | Hs | Mm |
| Co-stimulation receptor | ICOSLG | inducible T cell costimulator ligand | 23470321 | 23308 | 50723 |
| (14) | CD70 | CD70 molecule | 23470321 | 970 | 21948 |
| | TNFSF14 | TNF superfamily member 14 | 23470321 | 8740 | 50930 |
| | CD40 | CD40 molecule | 23470321 | 958 | 21939 |
| | TNFSF9 | TNF superfamily member 9 | 23470321 | 8744 | 21950 |
| | TNFSF4 | TNF superfamily member 4 | 23470321 | 7292 | 22164 |
| | TNFSF15 | TNF superfamily member 15 | 23470321 | 9966 | 326623 |
| | TNFSF18 | TNF superfamily member 18 | 23470321 | 8995 | 240873 |
| | TNFSF8 | TNF superfamily member 8 | 23470321 | 944 | 21949 |
| | TIMD4 | T cell immunoglobulin and mucin domain containing 4 | 23470321 | 91937 | 276891 |
| | SLAMF1 | signaling lymphocytic activation molecule family member 1 | 23470321 | 6504 | 27218 |
| | CD48 | CD48 molecule | 23470321 | 962 | 12506 |
| | SEMA4A | semaphorin 4A | 23470321 | 64218 | 20351 |
| | CD58 | CD58 molecule | 23470321 | 965 | / |
| Co-inhibition receptor | LGALS9 | galectin 9 | 23470321 | 3965 | 16859 |
| (10) | NECTIN3 | nectin cell adhesion molecule 3 | 23470321 | 25945 | 58998 |
| | TNFRSF14 | TNF receptor superfamily member 14 | 23470321 | 8764 | 230979 |
| | PDCD1LG2 | programmed cell death 1 ligand 2 | 23470321 | 80380 | 58205 |
| | CD274 | CD274 molecule | 23470321 | 29126 | 60533 |
| | CD276 | CD276 molecule | 27192563 | 80381 | 102657 |
| | VTCN1 | V-set domain containing T cell activation inhibitor 1 | 27192563 | 79679 | 242122 |
| | VSIR | V-set immunoregulatory receptor | 27192563 | 64115 | 74048 |
| | HHLA2 | HERV-H LTR-associating 2 | 27192563 | 11148 | / |
| | BTNL2 | butyrophilin like 2 | 27192563 | 56244 | 547431 |
| Dual function receptor | CD80 | CD80 molecule | 23470321 | 941 | 12519 |
| (4) | CD86 | CD86 molecule | 23470321 | 942 | 12524 |
| | PVR | poliovirus receptor | 23470321 | 5817 | 52118 |
| | IL2RB | interleukin 2 receptor subunit beta | 23470321 | 3560 | 16185 |
| Total number | 28 | | | | |

## Slide 5
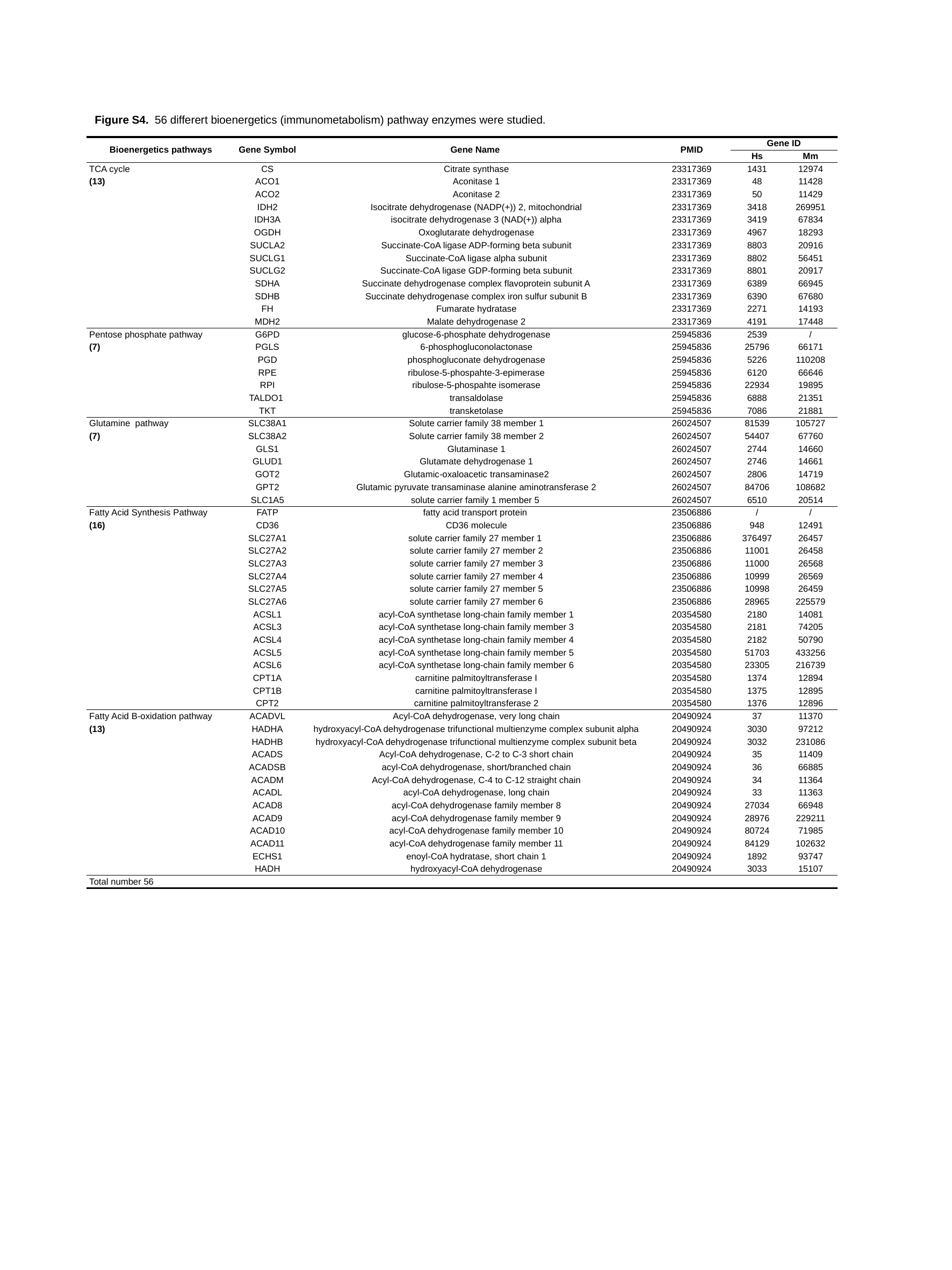

# Figure S4. 56 differert bioenergetics (immunometabolism) pathway enzymes were studied.
| Bioenergetics pathways | Gene Symbol | Gene Name | PMID | Gene ID | |
| --- | --- | --- | --- | --- | --- |
| | | | | Hs | Mm |
| TCA cycle | CS | Citrate synthase | 23317369 | 1431 | 12974 |
| (13) | ACO1 | Aconitase 1 | 23317369 | 48 | 11428 |
| | ACO2 | Aconitase 2 | 23317369 | 50 | 11429 |
| | IDH2 | Isocitrate dehydrogenase (NADP(+)) 2, mitochondrial | 23317369 | 3418 | 269951 |
| | IDH3A | isocitrate dehydrogenase 3 (NAD(+)) alpha | 23317369 | 3419 | 67834 |
| | OGDH | Oxoglutarate dehydrogenase | 23317369 | 4967 | 18293 |
| | SUCLA2 | Succinate-CoA ligase ADP-forming beta subunit | 23317369 | 8803 | 20916 |
| | SUCLG1 | Succinate-CoA ligase alpha subunit | 23317369 | 8802 | 56451 |
| | SUCLG2 | Succinate-CoA ligase GDP-forming beta subunit | 23317369 | 8801 | 20917 |
| | SDHA | Succinate dehydrogenase complex flavoprotein subunit A | 23317369 | 6389 | 66945 |
| | SDHB | Succinate dehydrogenase complex iron sulfur subunit B | 23317369 | 6390 | 67680 |
| | FH | Fumarate hydratase | 23317369 | 2271 | 14193 |
| | MDH2 | Malate dehydrogenase 2 | 23317369 | 4191 | 17448 |
| Pentose phosphate pathway | G6PD | glucose-6-phosphate dehydrogenase | 25945836 | 2539 | / |
| (7) | PGLS | 6-phosphogluconolactonase | 25945836 | 25796 | 66171 |
| | PGD | phosphogluconate dehydrogenase | 25945836 | 5226 | 110208 |
| | RPE | ribulose-5-phospahte-3-epimerase | 25945836 | 6120 | 66646 |
| | RPI | ribulose-5-phospahte isomerase | 25945836 | 22934 | 19895 |
| | TALDO1 | transaldolase | 25945836 | 6888 | 21351 |
| | TKT | transketolase | 25945836 | 7086 | 21881 |
| Glutamine pathway | SLC38A1 | Solute carrier family 38 member 1 | 26024507 | 81539 | 105727 |
| (7) | SLC38A2 | Solute carrier family 38 member 2 | 26024507 | 54407 | 67760 |
| | GLS1 | Glutaminase 1 | 26024507 | 2744 | 14660 |
| | GLUD1 | Glutamate dehydrogenase 1 | 26024507 | 2746 | 14661 |
| | GOT2 | Glutamic-oxaloacetic transaminase2 | 26024507 | 2806 | 14719 |
| | GPT2 | Glutamic pyruvate transaminase alanine aminotransferase 2 | 26024507 | 84706 | 108682 |
| | SLC1A5 | solute carrier family 1 member 5 | 26024507 | 6510 | 20514 |
| Fatty Acid Synthesis Pathway | FATP | fatty acid transport protein | 23506886 | / | / |
| (16) | CD36 | CD36 molecule | 23506886 | 948 | 12491 |
| | SLC27A1 | solute carrier family 27 member 1 | 23506886 | 376497 | 26457 |
| | SLC27A2 | solute carrier family 27 member 2 | 23506886 | 11001 | 26458 |
| | SLC27A3 | solute carrier family 27 member 3 | 23506886 | 11000 | 26568 |
| | SLC27A4 | solute carrier family 27 member 4 | 23506886 | 10999 | 26569 |
| | SLC27A5 | solute carrier family 27 member 5 | 23506886 | 10998 | 26459 |
| | SLC27A6 | solute carrier family 27 member 6 | 23506886 | 28965 | 225579 |
| | ACSL1 | acyl-CoA synthetase long-chain family member 1 | 20354580 | 2180 | 14081 |
| | ACSL3 | acyl-CoA synthetase long-chain family member 3 | 20354580 | 2181 | 74205 |
| | ACSL4 | acyl-CoA synthetase long-chain family member 4 | 20354580 | 2182 | 50790 |
| | ACSL5 | acyl-CoA synthetase long-chain family member 5 | 20354580 | 51703 | 433256 |
| | ACSL6 | acyl-CoA synthetase long-chain family member 6 | 20354580 | 23305 | 216739 |
| | CPT1A | carnitine palmitoyltransferase I | 20354580 | 1374 | 12894 |
| | CPT1B | carnitine palmitoyltransferase I | 20354580 | 1375 | 12895 |
| | CPT2 | carnitine palmitoyltransferase 2 | 20354580 | 1376 | 12896 |
| Fatty Acid B-oxidation pathway | ACADVL | Acyl-CoA dehydrogenase, very long chain | 20490924 | 37 | 11370 |
| (13) | HADHA | hydroxyacyl-CoA dehydrogenase trifunctional multienzyme complex subunit alpha | 20490924 | 3030 | 97212 |
| | HADHB | hydroxyacyl-CoA dehydrogenase trifunctional multienzyme complex subunit beta | 20490924 | 3032 | 231086 |
| | ACADS | Acyl-CoA dehydrogenase, C-2 to C-3 short chain | 20490924 | 35 | 11409 |
| | ACADSB | acyl-CoA dehydrogenase, short/branched chain | 20490924 | 36 | 66885 |
| | ACADM | Acyl-CoA dehydrogenase, C-4 to C-12 straight chain | 20490924 | 34 | 11364 |
| | ACADL | acyl-CoA dehydrogenase, long chain | 20490924 | 33 | 11363 |
| | ACAD8 | acyl-CoA dehydrogenase family member 8 | 20490924 | 27034 | 66948 |
| | ACAD9 | acyl-CoA dehydrogenase family member 9 | 20490924 | 28976 | 229211 |
| | ACAD10 | acyl-CoA dehydrogenase family member 10 | 20490924 | 80724 | 71985 |
| | ACAD11 | acyl-CoA dehydrogenase family member 11 | 20490924 | 84129 | 102632 |
| | ECHS1 | enoyl-CoA hydratase, short chain 1 | 20490924 | 1892 | 93747 |
| | HADH | hydroxyacyl-CoA dehydrogenase | 20490924 | 3033 | 15107 |
| Total number 56 | | | | | |

## Slide 6
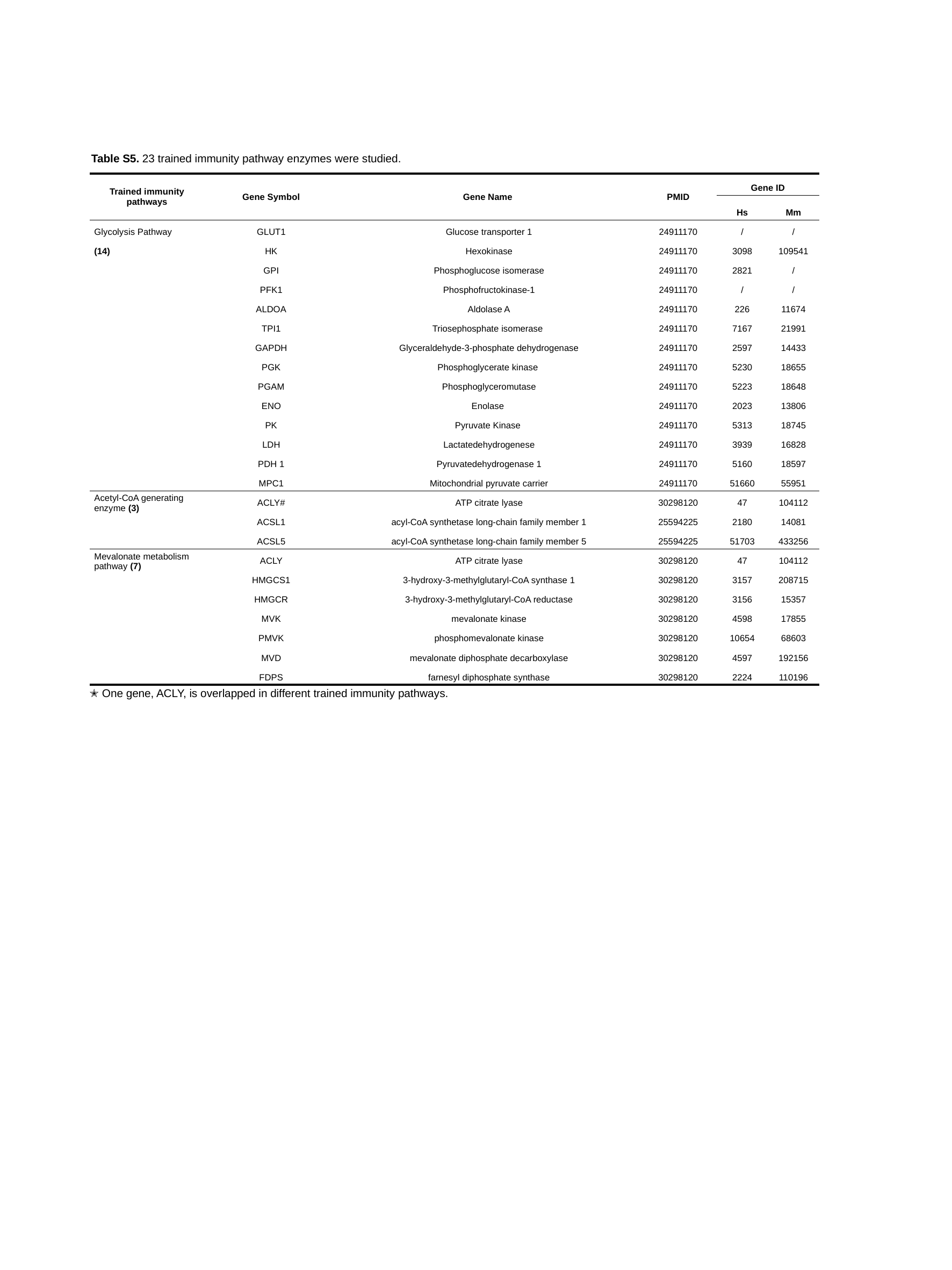

Table S5. 23 trained immunity pathway enzymes were studied.
| Trained immunity pathways | Gene Symbol | Gene Name | PMID | Gene ID | |
| --- | --- | --- | --- | --- | --- |
| | | | | Hs | Mm |
| Glycolysis Pathway | GLUT1 | Glucose transporter 1 | 24911170 | / | / |
| (14) | HK | Hexokinase | 24911170 | 3098 | 109541 |
| | GPI | Phosphoglucose isomerase | 24911170 | 2821 | / |
| | PFK1 | Phosphofructokinase-1 | 24911170 | / | / |
| | ALDOA | Aldolase A | 24911170 | 226 | 11674 |
| | TPI1 | Triosephosphate isomerase | 24911170 | 7167 | 21991 |
| | GAPDH | Glyceraldehyde-3-phosphate dehydrogenase | 24911170 | 2597 | 14433 |
| | PGK | Phosphoglycerate kinase | 24911170 | 5230 | 18655 |
| | PGAM | Phosphoglyceromutase | 24911170 | 5223 | 18648 |
| | ENO | Enolase | 24911170 | 2023 | 13806 |
| | PK | Pyruvate Kinase | 24911170 | 5313 | 18745 |
| | LDH | Lactatedehydrogenese | 24911170 | 3939 | 16828 |
| | PDH 1 | Pyruvatedehydrogenase 1 | 24911170 | 5160 | 18597 |
| | MPC1 | Mitochondrial pyruvate carrier | 24911170 | 51660 | 55951 |
| Acetyl-CoA generating enzyme (3) | ACLY# | ATP citrate lyase | 30298120 | 47 | 104112 |
| | ACSL1 | acyl-CoA synthetase long-chain family member 1 | 25594225 | 2180 | 14081 |
| | ACSL5 | acyl-CoA synthetase long-chain family member 5 | 25594225 | 51703 | 433256 |
| Mevalonate metabolism pathway (7) | ACLY | ATP citrate lyase | 30298120 | 47 | 104112 |
| | HMGCS1 | 3-hydroxy-3-methylglutaryl-CoA synthase 1 | 30298120 | 3157 | 208715 |
| | HMGCR | 3-hydroxy-3-methylglutaryl-CoA reductase | 30298120 | 3156 | 15357 |
| | MVK | mevalonate kinase | 30298120 | 4598 | 17855 |
| | PMVK | phosphomevalonate kinase | 30298120 | 10654 | 68603 |
| | MVD | mevalonate diphosphate decarboxylase | 30298120 | 4597 | 192156 |
| | FDPS | farnesyl diphosphate synthase | 30298120 | 2224 | 110196 |
 ✭ One gene, ACLY, is overlapped in different trained immunity pathways.

## Slide 7
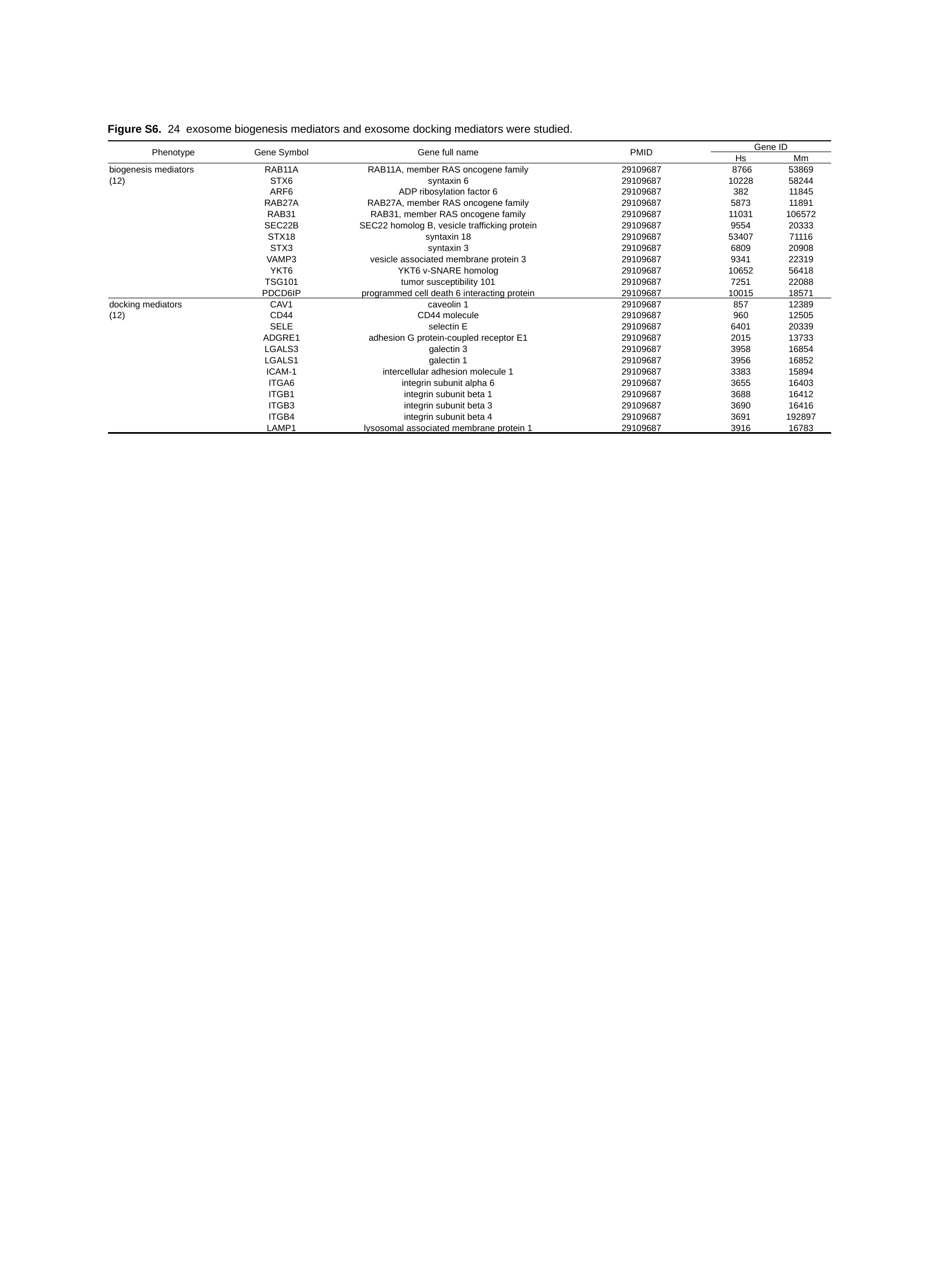

# Figure S6. 24 exosome biogenesis mediators and exosome docking mediators were studied.
| Phenotype | Gene Symbol | Gene full name | PMID | Gene ID | |
| --- | --- | --- | --- | --- | --- |
| | | | | Hs | Mm |
| biogenesis mediators | RAB11A | RAB11A, member RAS oncogene family | 29109687 | 8766 | 53869 |
| (12) | STX6 | syntaxin 6 | 29109687 | 10228 | 58244 |
| | ARF6 | ADP ribosylation factor 6 | 29109687 | 382 | 11845 |
| | RAB27A | RAB27A, member RAS oncogene family | 29109687 | 5873 | 11891 |
| | RAB31 | RAB31, member RAS oncogene family | 29109687 | 11031 | 106572 |
| | SEC22B | SEC22 homolog B, vesicle trafficking protein | 29109687 | 9554 | 20333 |
| | STX18 | syntaxin 18 | 29109687 | 53407 | 71116 |
| | STX3 | syntaxin 3 | 29109687 | 6809 | 20908 |
| | VAMP3 | vesicle associated membrane protein 3 | 29109687 | 9341 | 22319 |
| | YKT6 | YKT6 v-SNARE homolog | 29109687 | 10652 | 56418 |
| | TSG101 | tumor susceptibility 101 | 29109687 | 7251 | 22088 |
| | PDCD6IP | programmed cell death 6 interacting protein | 29109687 | 10015 | 18571 |
| docking mediators | CAV1 | caveolin 1 | 29109687 | 857 | 12389 |
| (12) | CD44 | CD44 molecule | 29109687 | 960 | 12505 |
| | SELE | selectin E | 29109687 | 6401 | 20339 |
| | ADGRE1 | adhesion G protein-coupled receptor E1 | 29109687 | 2015 | 13733 |
| | LGALS3 | galectin 3 | 29109687 | 3958 | 16854 |
| | LGALS1 | galectin 1 | 29109687 | 3956 | 16852 |
| | ICAM-1 | intercellular adhesion molecule 1 | 29109687 | 3383 | 15894 |
| | ITGA6 | integrin subunit alpha 6 | 29109687 | 3655 | 16403 |
| | ITGB1 | integrin subunit beta 1 | 29109687 | 3688 | 16412 |
| | ITGB3 | integrin subunit beta 3 | 29109687 | 3690 | 16416 |
| | ITGB4 | integrin subunit beta 4 | 29109687 | 3691 | 192897 |
| | LAMP1 | lysosomal associated membrane protein 1 | 29109687 | 3916 | 16783 |

## Slide 8
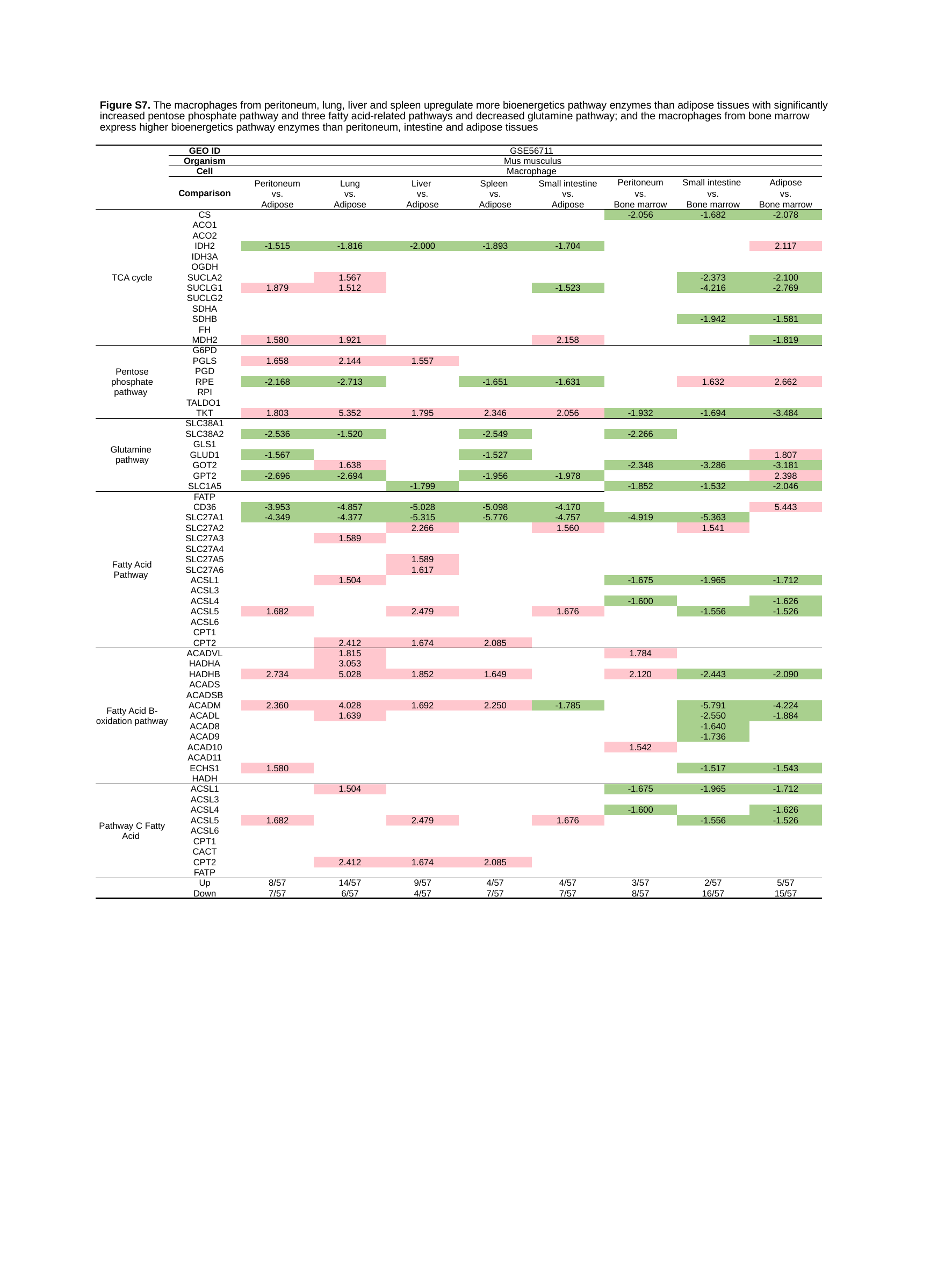

# Figure S7. The macrophages from peritoneum, lung, liver and spleen upregulate more bioenergetics pathway enzymes than adipose tissues with significantly increased pentose phosphate pathway and three fatty acid-related pathways and decreased glutamine pathway; and the macrophages from bone marrow express higher bioenergetics pathway enzymes than peritoneum, intestine and adipose tissues
| | GEO ID | GSE56711 | | | | | | | |
| --- | --- | --- | --- | --- | --- | --- | --- | --- | --- |
| | Organism | Mus musculus | | | | | | | |
| | Cell | Macrophage | | | | | | | |
| | Comparison | Peritoneum | Lung | Liver | Spleen | Small intestine | Peritoneum | Small intestine | Adipose |
| | | vs. | vs. | vs. | vs. | vs. | vs. | vs. | vs. |
| | | Adipose | Adipose | Adipose | Adipose | Adipose | Bone marrow | Bone marrow | Bone marrow |
| TCA cycle | CS | | | | | | -2.056 | -1.682 | -2.078 |
| | ACO1 | | | | | | | | |
| | ACO2 | | | | | | | | |
| | IDH2 | -1.515 | -1.816 | -2.000 | -1.893 | -1.704 | | | 2.117 |
| | IDH3A | | | | | | | | |
| | OGDH | | | | | | | | |
| | SUCLA2 | | 1.567 | | | | | -2.373 | -2.100 |
| | SUCLG1 | 1.879 | 1.512 | | | -1.523 | | -4.216 | -2.769 |
| | SUCLG2 | | | | | | | | |
| | SDHA | | | | | | | | |
| | SDHB | | | | | | | -1.942 | -1.581 |
| | FH | | | | | | | | |
| | MDH2 | 1.580 | 1.921 | | | 2.158 | | | -1.819 |
| Pentose phosphate pathway | G6PD | | | | | | | | |
| | PGLS | 1.658 | 2.144 | 1.557 | | | | | |
| | PGD | | | | | | | | |
| | RPE | -2.168 | -2.713 | | -1.651 | -1.631 | | 1.632 | 2.662 |
| | RPI | | | | | | | | |
| | TALDO1 | | | | | | | | |
| | TKT | 1.803 | 5.352 | 1.795 | 2.346 | 2.056 | -1.932 | -1.694 | -3.484 |
| Glutamine pathway | SLC38A1 | | | | | | | | |
| | SLC38A2 | -2.536 | -1.520 | | -2.549 | | -2.266 | | |
| | GLS1 | | | | | | | | |
| | GLUD1 | -1.567 | | | -1.527 | | | | 1.807 |
| | GOT2 | | 1.638 | | | | -2.348 | -3.286 | -3.181 |
| | GPT2 | -2.696 | -2.694 | | -1.956 | -1.978 | | | 2.398 |
| | SLC1A5 | | | -1.799 | | | -1.852 | -1.532 | -2.046 |
| Fatty Acid Pathway | FATP | | | | | | | | |
| | CD36 | -3.953 | -4.857 | -5.028 | -5.098 | -4.170 | | | 5.443 |
| | SLC27A1 | -4.349 | -4.377 | -5.315 | -5.776 | -4.757 | -4.919 | -5.363 | |
| | SLC27A2 | | | 2.266 | | 1.560 | | 1.541 | |
| | SLC27A3 | | 1.589 | | | | | | |
| | SLC27A4 | | | | | | | | |
| | SLC27A5 | | | 1.589 | | | | | |
| | SLC27A6 | | | 1.617 | | | | | |
| | ACSL1 | | 1.504 | | | | -1.675 | -1.965 | -1.712 |
| | ACSL3 | | | | | | | | |
| | ACSL4 | | | | | | -1.600 | | -1.626 |
| | ACSL5 | 1.682 | | 2.479 | | 1.676 | | -1.556 | -1.526 |
| | ACSL6 | | | | | | | | |
| | CPT1 | | | | | | | | |
| | CPT2 | | 2.412 | 1.674 | 2.085 | | | | |
| Fatty Acid B-oxidation pathway | ACADVL | | 1.815 | | | | 1.784 | | |
| | HADHA | | 3.053 | | | | | | |
| | HADHB | 2.734 | 5.028 | 1.852 | 1.649 | | 2.120 | -2.443 | -2.090 |
| | ACADS | | | | | | | | |
| | ACADSB | | | | | | | | |
| | ACADM | 2.360 | 4.028 | 1.692 | 2.250 | -1.785 | | -5.791 | -4.224 |
| | ACADL | | 1.639 | | | | | -2.550 | -1.884 |
| | ACAD8 | | | | | | | -1.640 | |
| | ACAD9 | | | | | | | -1.736 | |
| | ACAD10 | | | | | | 1.542 | | |
| | ACAD11 | | | | | | | | |
| | ECHS1 | 1.580 | | | | | | -1.517 | -1.543 |
| | HADH | | | | | | | | |
| Pathway C Fatty Acid | ACSL1 | | 1.504 | | | | -1.675 | -1.965 | -1.712 |
| | ACSL3 | | | | | | | | |
| | ACSL4 | | | | | | -1.600 | | -1.626 |
| | ACSL5 | 1.682 | | 2.479 | | 1.676 | | -1.556 | -1.526 |
| | ACSL6 | | | | | | | | |
| | CPT1 | | | | | | | | |
| | CACT | | | | | | | | |
| | CPT2 | | 2.412 | 1.674 | 2.085 | | | | |
| | FATP | | | | | | | | |
| | Up | 8/57 | 14/57 | 9/57 | 4/57 | 4/57 | 3/57 | 2/57 | 5/57 |
| | Down | 7/57 | 6/57 | 4/57 | 7/57 | 7/57 | 8/57 | 16/57 | 15/57 |

## Slide 9
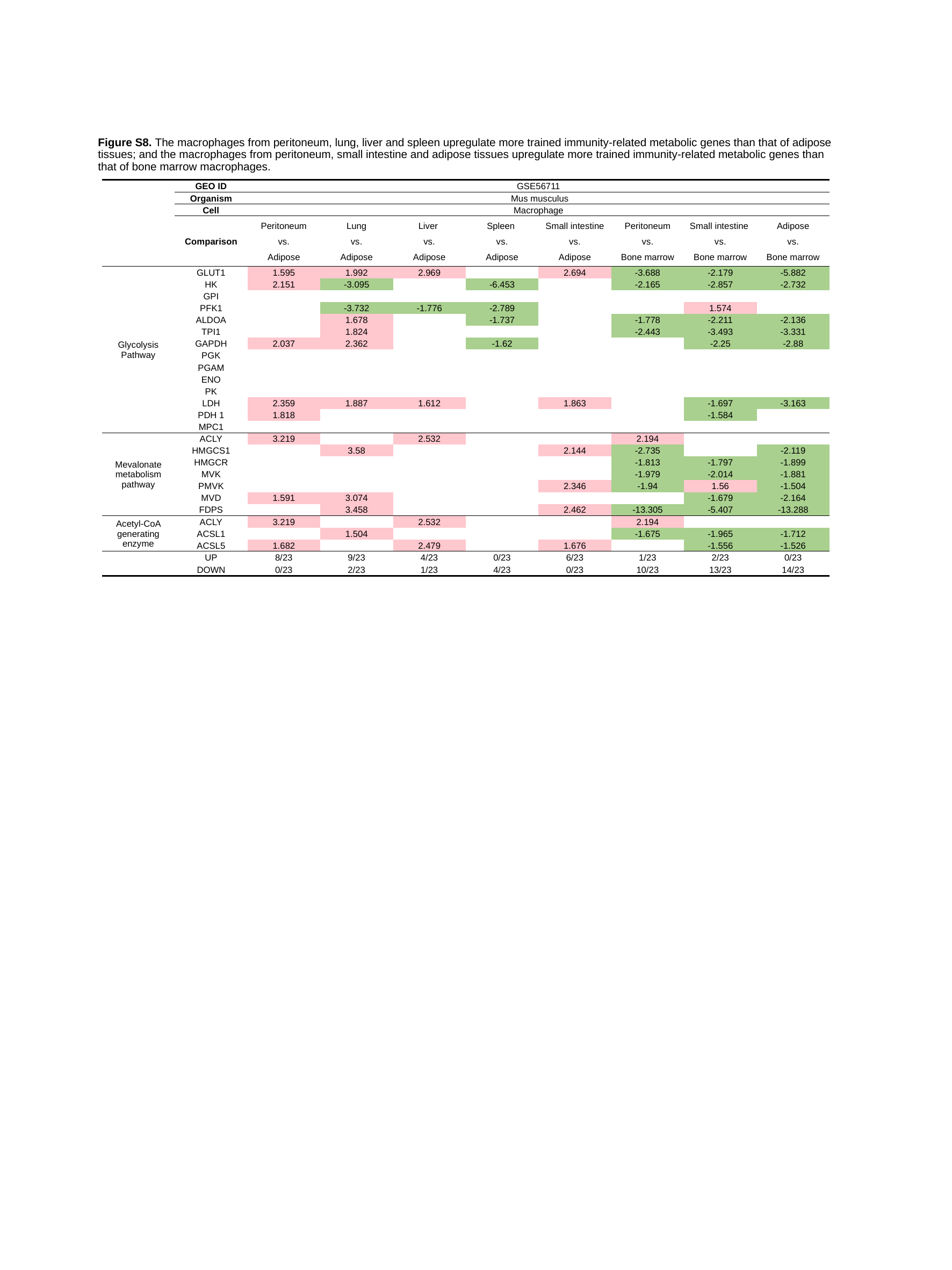

# Figure S8. The macrophages from peritoneum, lung, liver and spleen upregulate more trained immunity-related metabolic genes than that of adipose tissues; and the macrophages from peritoneum, small intestine and adipose tissues upregulate more trained immunity-related metabolic genes than that of bone marrow macrophages.
| | GEO ID | GSE56711 | | | | | | | |
| --- | --- | --- | --- | --- | --- | --- | --- | --- | --- |
| | Organism | Mus musculus | | | | | | | |
| | Cell | Macrophage | | | | | | | |
| | Comparison | Peritoneum | Lung | Liver | Spleen | Small intestine | Peritoneum | Small intestine | Adipose |
| | | vs. | vs. | vs. | vs. | vs. | vs. | vs. | vs. |
| | | Adipose | Adipose | Adipose | Adipose | Adipose | Bone marrow | Bone marrow | Bone marrow |
| Glycolysis Pathway | GLUT1 | 1.595 | 1.992 | 2.969 | | 2.694 | -3.688 | -2.179 | -5.882 |
| | HK | 2.151 | -3.095 | | -6.453 | | -2.165 | -2.857 | -2.732 |
| | GPI | | | | | | | | |
| | PFK1 | | -3.732 | -1.776 | -2.789 | | | 1.574 | |
| | ALDOA | | 1.678 | | -1.737 | | -1.778 | -2.211 | -2.136 |
| | TPI1 | | 1.824 | | | | -2.443 | -3.493 | -3.331 |
| | GAPDH | 2.037 | 2.362 | | -1.62 | | | -2.25 | -2.88 |
| | PGK | | | | | | | | |
| | PGAM | | | | | | | | |
| | ENO | | | | | | | | |
| | PK | | | | | | | | |
| | LDH | 2.359 | 1.887 | 1.612 | | 1.863 | | -1.697 | -3.163 |
| | PDH 1 | 1.818 | | | | | | -1.584 | |
| | MPC1 | | | | | | | | |
| Mevalonate metabolism pathway | ACLY | 3.219 | | 2.532 | | | 2.194 | | |
| | HMGCS1 | | 3.58 | | | 2.144 | -2.735 | | -2.119 |
| | HMGCR | | | | | | -1.813 | -1.797 | -1.899 |
| | MVK | | | | | | -1.979 | -2.014 | -1.881 |
| | PMVK | | | | | 2.346 | -1.94 | 1.56 | -1.504 |
| | MVD | 1.591 | 3.074 | | | | | -1.679 | -2.164 |
| | FDPS | | 3.458 | | | 2.462 | -13.305 | -5.407 | -13.288 |
| Acetyl-CoA generating enzyme | ACLY | 3.219 | | 2.532 | | | 2.194 | | |
| | ACSL1 | | 1.504 | | | | -1.675 | -1.965 | -1.712 |
| | ACSL5 | 1.682 | | 2.479 | | 1.676 | | -1.556 | -1.526 |
| | UP | 8/23 | 9/23 | 4/23 | 0/23 | 6/23 | 1/23 | 2/23 | 0/23 |
| | DOWN | 0/23 | 2/23 | 1/23 | 4/23 | 0/23 | 10/23 | 13/23 | 14/23 |

## Slide 10
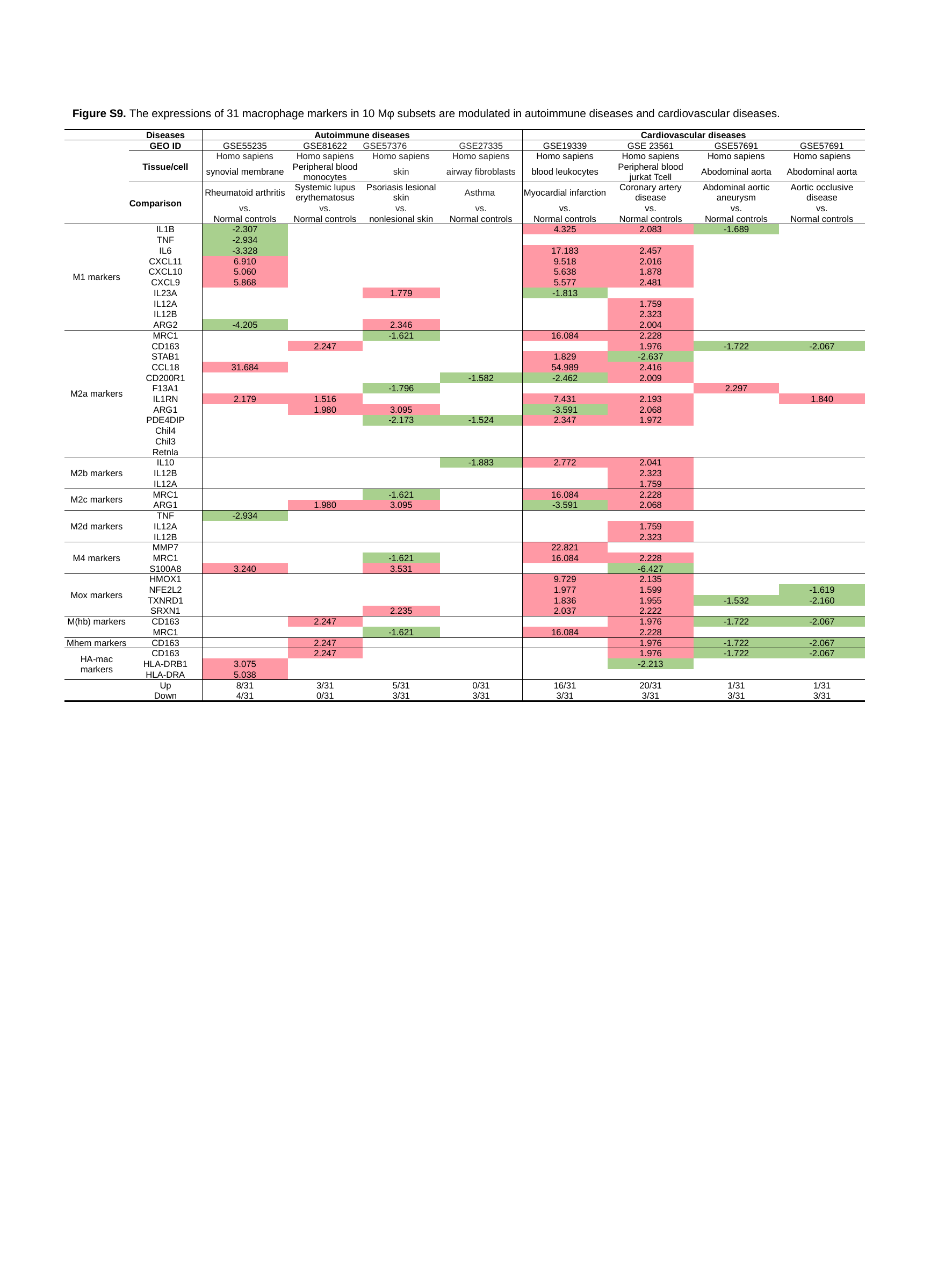

Figure S9. The expressions of 31 macrophage markers in 10 Mφ subsets are modulated in autoimmune diseases and cardiovascular diseases.
| | Diseases | Autoimmune diseases | | | | Cardiovascular diseases | | | |
| --- | --- | --- | --- | --- | --- | --- | --- | --- | --- |
| | GEO ID | GSE55235 | GSE81622 | GSE57376 | GSE27335 | GSE19339 | GSE 23561 | GSE57691 | GSE57691 |
| | Tissue/cell | Homo sapiens | Homo sapiens | Homo sapiens | Homo sapiens | Homo sapiens | Homo sapiens | Homo sapiens | Homo sapiens |
| | | synovial membrane | Peripheral blood monocytes | skin | airway fibroblasts | blood leukocytes | Peripheral blood jurkat Tcell | Abodominal aorta | Abodominal aorta |
| | Comparison | Rheumatoid arthritis | Systemic lupus erythematosus | Psoriasis lesional skin | Asthma | Myocardial infarction | Coronary artery disease | Abdominal aortic aneurysm | Aortic occlusive disease |
| | | vs. | vs. | vs. | vs. | vs. | vs. | vs. | vs. |
| | | Normal controls | Normal controls | nonlesional skin | Normal controls | Normal controls | Normal controls | Normal controls | Normal controls |
| M1 markers | IL1B | -2.307 | | | | 4.325 | 2.083 | -1.689 | |
| | TNF | -2.934 | | | | | | | |
| | IL6 | -3.328 | | | | 17.183 | 2.457 | | |
| | CXCL11 | 6.910 | | | | 9.518 | 2.016 | | |
| | CXCL10 | 5.060 | | | | 5.638 | 1.878 | | |
| | CXCL9 | 5.868 | | | | 5.577 | 2.481 | | |
| | IL23A | | | 1.779 | | -1.813 | | | |
| | IL12A | | | | | | 1.759 | | |
| | IL12B | | | | | | 2.323 | | |
| | ARG2 | -4.205 | | 2.346 | | | 2.004 | | |
| M2a markers | MRC1 | | | -1.621 | | 16.084 | 2.228 | | |
| | CD163 | | 2.247 | | | | 1.976 | -1.722 | -2.067 |
| | STAB1 | | | | | 1.829 | -2.637 | | |
| | CCL18 | 31.684 | | | | 54.989 | 2.416 | | |
| | CD200R1 | | | | -1.582 | -2.462 | 2.009 | | |
| | F13A1 | | | -1.796 | | | | 2.297 | |
| | IL1RN | 2.179 | 1.516 | | | 7.431 | 2.193 | | 1.840 |
| | ARG1 | | 1.980 | 3.095 | | -3.591 | 2.068 | | |
| | PDE4DIP | | | -2.173 | -1.524 | 2.347 | 1.972 | | |
| | Chil4 | | | | | | | | |
| | Chil3 | | | | | | | | |
| | Retnla | | | | | | | | |
| M2b markers | IL10 | | | | -1.883 | 2.772 | 2.041 | | |
| | IL12B | | | | | | 2.323 | | |
| | IL12A | | | | | | 1.759 | | |
| M2c markers | MRC1 | | | -1.621 | | 16.084 | 2.228 | | |
| | ARG1 | | 1.980 | 3.095 | | -3.591 | 2.068 | | |
| M2d markers | TNF | -2.934 | | | | | | | |
| | IL12A | | | | | | 1.759 | | |
| | IL12B | | | | | | 2.323 | | |
| M4 markers | MMP7 | | | | | 22.821 | | | |
| | MRC1 | | | -1.621 | | 16.084 | 2.228 | | |
| | S100A8 | 3.240 | | 3.531 | | | -6.427 | | |
| Mox markers | HMOX1 | | | | | 9.729 | 2.135 | | |
| | NFE2L2 | | | | | 1.977 | 1.599 | | -1.619 |
| | TXNRD1 | | | | | 1.836 | 1.955 | -1.532 | -2.160 |
| | SRXN1 | | | 2.235 | | 2.037 | 2.222 | | |
| M(hb) markers | CD163 | | 2.247 | | | | 1.976 | -1.722 | -2.067 |
| | MRC1 | | | -1.621 | | 16.084 | 2.228 | | |
| Mhem markers | CD163 | | 2.247 | | | | 1.976 | -1.722 | -2.067 |
| HA-mac markers | CD163 | | 2.247 | | | | 1.976 | -1.722 | -2.067 |
| | HLA-DRB1 | 3.075 | | | | | -2.213 | | |
| | HLA-DRA | 5.038 | | | | | | | |
| | Up | 8/31 | 3/31 | 5/31 | 0/31 | 16/31 | 20/31 | 1/31 | 1/31 |
| | Down | 4/31 | 0/31 | 3/31 | 3/31 | 3/31 | 3/31 | 3/31 | 3/31 |

## Slide 11
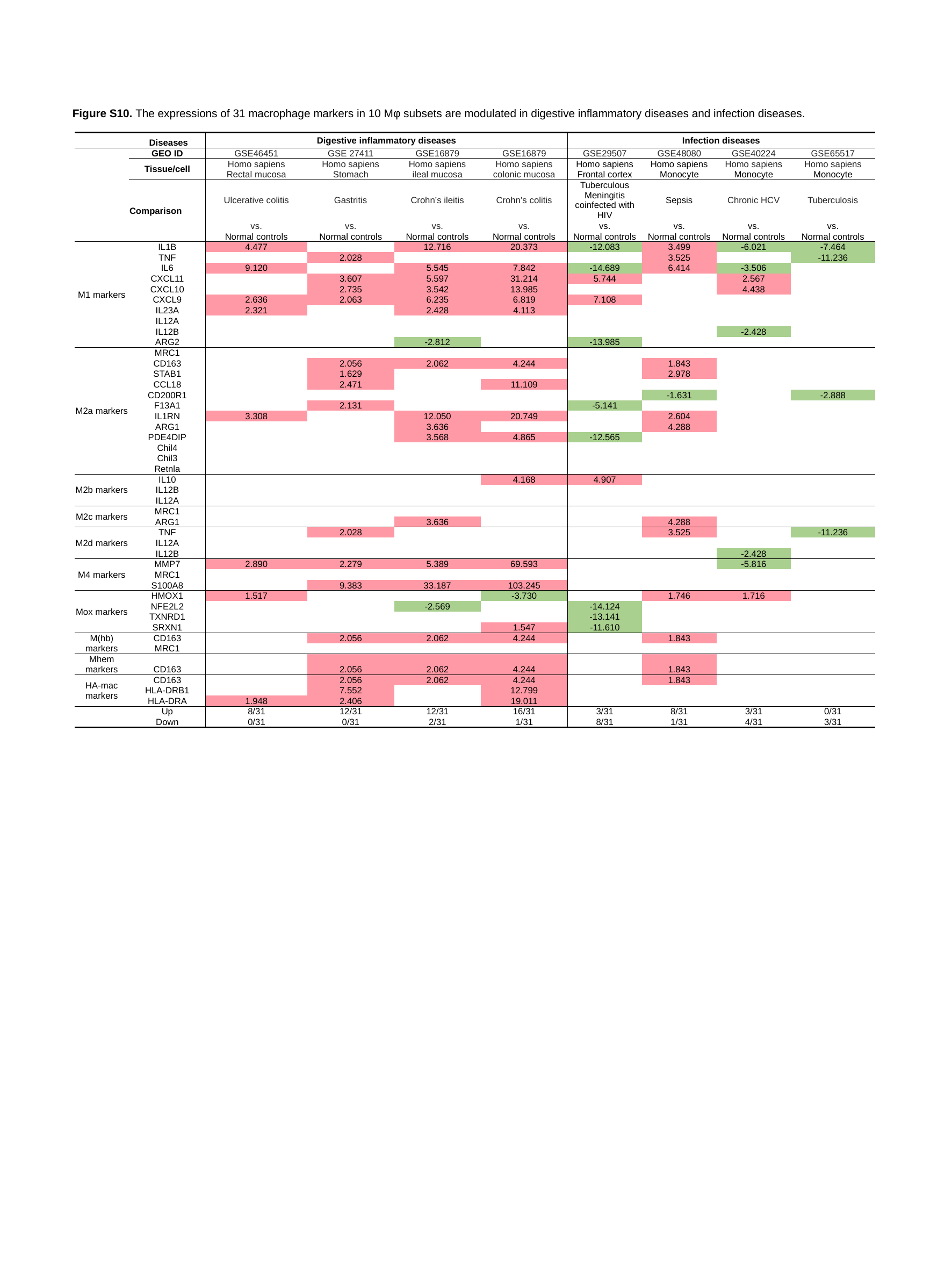

Figure S10. The expressions of 31 macrophage markers in 10 Mφ subsets are modulated in digestive inflammatory diseases and infection diseases.
| | Diseases | Digestive inflammatory diseases | | | | Infection diseases | | | |
| --- | --- | --- | --- | --- | --- | --- | --- | --- | --- |
| | GEO ID | GSE46451 | GSE 27411 | GSE16879 | GSE16879 | GSE29507 | GSE48080 | GSE40224 | GSE65517 |
| | Tissue/cell | Homo sapiens | Homo sapiens | Homo sapiens | Homo sapiens | Homo sapiens | Homo sapiens | Homo sapiens | Homo sapiens |
| | | Rectal mucosa | Stomach | ileal mucosa | colonic mucosa | Frontal cortex | Monocyte | Monocyte | Monocyte |
| | Comparison | Ulcerative colitis | Gastritis | Crohn’s ileitis | Crohn’s colitis | Tuberculous Meningitis coinfected with HIV | Sepsis | Chronic HCV | Tuberculosis |
| | | vs. | vs. | vs. | vs. | vs. | vs. | vs. | vs. |
| | | Normal controls | Normal controls | Normal controls | Normal controls | Normal controls | Normal controls | Normal controls | Normal controls |
| M1 markers | IL1B | 4.477 | | 12.716 | 20.373 | -12.083 | 3.499 | -6.021 | -7.464 |
| | TNF | | 2.028 | | | | 3.525 | | -11.236 |
| | IL6 | 9.120 | | 5.545 | 7.842 | -14.689 | 6.414 | -3.506 | |
| | CXCL11 | | 3.607 | 5.597 | 31.214 | 5.744 | | 2.567 | |
| | CXCL10 | | 2.735 | 3.542 | 13.985 | | | 4.438 | |
| | CXCL9 | 2.636 | 2.063 | 6.235 | 6.819 | 7.108 | | | |
| | IL23A | 2.321 | | 2.428 | 4.113 | | | | |
| | IL12A | | | | | | | | |
| | IL12B | | | | | | | -2.428 | |
| | ARG2 | | | -2.812 | | -13.985 | | | |
| M2a markers | MRC1 | | | | | | | | |
| | CD163 | | 2.056 | 2.062 | 4.244 | | 1.843 | | |
| | STAB1 | | 1.629 | | | | 2.978 | | |
| | CCL18 | | 2.471 | | 11.109 | | | | |
| | CD200R1 | | | | | | -1.631 | | -2.888 |
| | F13A1 | | 2.131 | | | -5.141 | | | |
| | IL1RN | 3.308 | | 12.050 | 20.749 | | 2.604 | | |
| | ARG1 | | | 3.636 | | | 4.288 | | |
| | PDE4DIP | | | 3.568 | 4.865 | -12.565 | | | |
| | Chil4 | | | | | | | | |
| | Chil3 | | | | | | | | |
| | Retnla | | | | | | | | |
| M2b markers | IL10 | | | | 4.168 | 4.907 | | | |
| | IL12B | | | | | | | | |
| | IL12A | | | | | | | | |
| M2c markers | MRC1 | | | | | | | | |
| | ARG1 | | | 3.636 | | | 4.288 | | |
| M2d markers | TNF | | 2.028 | | | | 3.525 | | -11.236 |
| | IL12A | | | | | | | | |
| | IL12B | | | | | | | -2.428 | |
| M4 markers | MMP7 | 2.890 | 2.279 | 5.389 | 69.593 | | | -5.816 | |
| | MRC1 | | | | | | | | |
| | S100A8 | | 9.383 | 33.187 | 103.245 | | | | |
| Mox markers | HMOX1 | 1.517 | | | -3.730 | | 1.746 | 1.716 | |
| | NFE2L2 | | | -2.569 | | -14.124 | | | |
| | TXNRD1 | | | | | -13.141 | | | |
| | SRXN1 | | | | 1.547 | -11.610 | | | |
| M(hb) markers | CD163 | | 2.056 | 2.062 | 4.244 | | 1.843 | | |
| | MRC1 | | | | | | | | |
| Mhem markers | CD163 | | 2.056 | 2.062 | 4.244 | | 1.843 | | |
| HA-mac markers | CD163 | | 2.056 | 2.062 | 4.244 | | 1.843 | | |
| | HLA-DRB1 | | 7.552 | | 12.799 | | | | |
| | HLA-DRA | 1.948 | 2.406 | | 19.011 | | | | |
| | Up | 8/31 | 12/31 | 12/31 | 16/31 | 3/31 | 8/31 | 3/31 | 0/31 |
| | Down | 0/31 | 0/31 | 2/31 | 1/31 | 8/31 | 1/31 | 4/31 | 3/31 |

## Slide 12
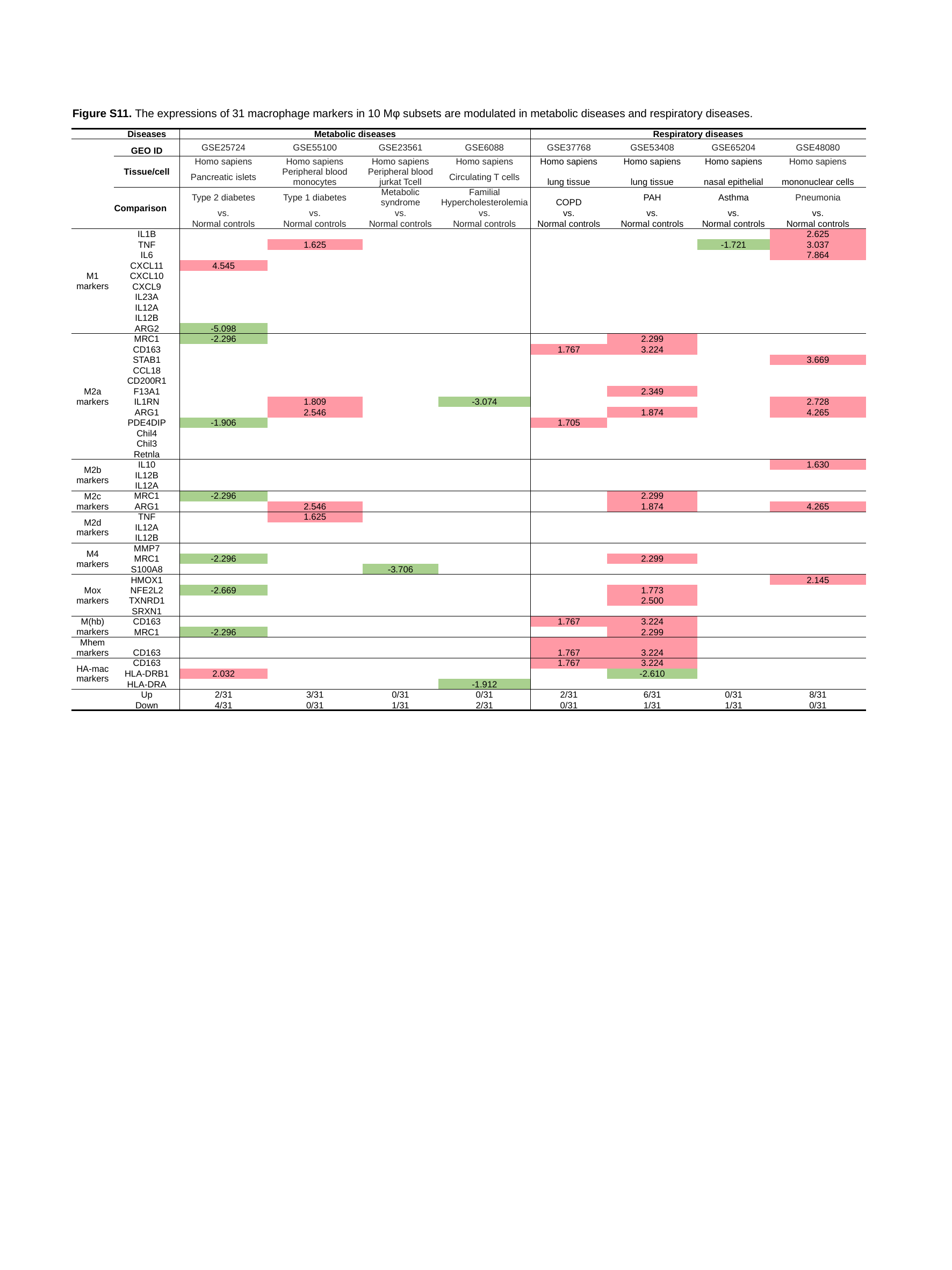

Figure S11. The expressions of 31 macrophage markers in 10 Mφ subsets are modulated in metabolic diseases and respiratory diseases.
| | Diseases | Metabolic diseases | | | | Respiratory diseases | | | |
| --- | --- | --- | --- | --- | --- | --- | --- | --- | --- |
| | GEO ID | GSE25724 | GSE55100 | GSE23561 | GSE6088 | GSE37768 | GSE53408 | GSE65204 | GSE48080 |
| | Tissue/cell | Homo sapiens | Homo sapiens | Homo sapiens | Homo sapiens | Homo sapiens | Homo sapiens | Homo sapiens | Homo sapiens |
| | | Pancreatic islets | Peripheral blood monocytes | Peripheral blood jurkat Tcell | Circulating T cells | lung tissue | lung tissue | nasal epithelial | mononuclear cells |
| | Comparison | Type 2 diabetes | Type 1 diabetes | Metabolic syndrome | Familial Hypercholesterolemia | COPD | PAH | Asthma | Pneumonia |
| | | vs. | vs. | vs. | vs. | vs. | vs. | vs. | vs. |
| | | Normal controls | Normal controls | Normal controls | Normal controls | Normal controls | Normal controls | Normal controls | Normal controls |
| M1 markers | IL1B | | | | | | | | 2.625 |
| | TNF | | 1.625 | | | | | -1.721 | 3.037 |
| | IL6 | | | | | | | | 7.864 |
| | CXCL11 | 4.545 | | | | | | | |
| | CXCL10 | | | | | | | | |
| | CXCL9 | | | | | | | | |
| | IL23A | | | | | | | | |
| | IL12A | | | | | | | | |
| | IL12B | | | | | | | | |
| | ARG2 | -5.098 | | | | | | | |
| M2a markers | MRC1 | -2.296 | | | | | 2.299 | | |
| | CD163 | | | | | 1.767 | 3.224 | | |
| | STAB1 | | | | | | | | 3.669 |
| | CCL18 | | | | | | | | |
| | CD200R1 | | | | | | | | |
| | F13A1 | | | | | | 2.349 | | |
| | IL1RN | | 1.809 | | -3.074 | | | | 2.728 |
| | ARG1 | | 2.546 | | | | 1.874 | | 4.265 |
| | PDE4DIP | -1.906 | | | | 1.705 | | | |
| | Chil4 | | | | | | | | |
| | Chil3 | | | | | | | | |
| | Retnla | | | | | | | | |
| M2b markers | IL10 | | | | | | | | 1.630 |
| | IL12B | | | | | | | | |
| | IL12A | | | | | | | | |
| M2c markers | MRC1 | -2.296 | | | | | 2.299 | | |
| | ARG1 | | 2.546 | | | | 1.874 | | 4.265 |
| M2d markers | TNF | | 1.625 | | | | | | |
| | IL12A | | | | | | | | |
| | IL12B | | | | | | | | |
| M4 markers | MMP7 | | | | | | | | |
| | MRC1 | -2.296 | | | | | 2.299 | | |
| | S100A8 | | | -3.706 | | | | | |
| Mox markers | HMOX1 | | | | | | | | 2.145 |
| | NFE2L2 | -2.669 | | | | | 1.773 | | |
| | TXNRD1 | | | | | | 2.500 | | |
| | SRXN1 | | | | | | | | |
| M(hb) markers | CD163 | | | | | 1.767 | 3.224 | | |
| | MRC1 | -2.296 | | | | | 2.299 | | |
| Mhem markers | CD163 | | | | | 1.767 | 3.224 | | |
| HA-mac markers | CD163 | | | | | 1.767 | 3.224 | | |
| | HLA-DRB1 | 2.032 | | | | | -2.610 | | |
| | HLA-DRA | | | | -1.912 | | | | |
| | Up | 2/31 | 3/31 | 0/31 | 0/31 | 2/31 | 6/31 | 0/31 | 8/31 |
| | Down | 4/31 | 0/31 | 1/31 | 2/31 | 0/31 | 1/31 | 1/31 | 0/31 |

## Slide 13
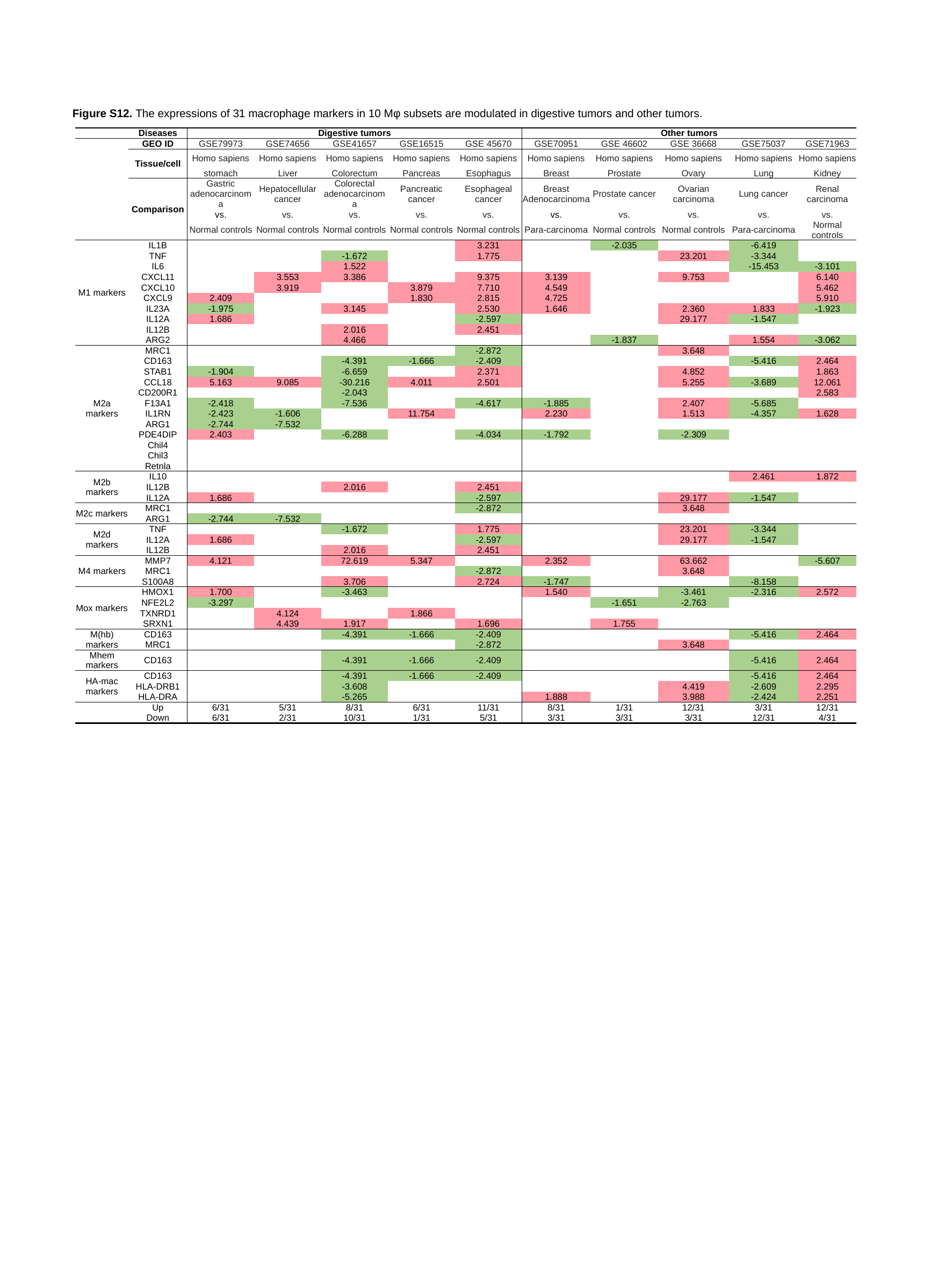

Figure S12. The expressions of 31 macrophage markers in 10 Mφ subsets are modulated in digestive tumors and other tumors.
| | Diseases | Digestive tumors | | | | | Other tumors | | | | |
| --- | --- | --- | --- | --- | --- | --- | --- | --- | --- | --- | --- |
| | GEO ID | GSE79973 | GSE74656 | GSE41657 | GSE16515 | GSE 45670 | GSE70951 | GSE 46602 | GSE 36668 | GSE75037 | GSE71963 |
| | Tissue/cell | Homo sapiens | Homo sapiens | Homo sapiens | Homo sapiens | Homo sapiens | Homo sapiens | Homo sapiens | Homo sapiens | Homo sapiens | Homo sapiens |
| | | stomach | Liver | Colorectum | Pancreas | Esophagus | Breast | Prostate | Ovary | Lung | Kidney |
| | Comparison | Gastric adenocarcinoma | Hepatocellular cancer | Colorectal adenocarcinoma | Pancreatic cancer | Esophageal cancer | Breast Adenocarcinoma | Prostate cancer | Ovarian carcinoma | Lung cancer | Renal carcinoma |
| | | vs. | vs. | vs. | vs. | vs. | vs. | vs. | vs. | vs. | vs. |
| | | Normal controls | Normal controls | Normal controls | Normal controls | Normal controls | Para-carcinoma | Normal controls | Normal controls | Para-carcinoma | Normal controls |
| M1 markers | IL1B | | | | | 3.231 | | -2.035 | | -6.419 | |
| | TNF | | | -1.672 | | 1.775 | | | 23.201 | -3.344 | |
| | IL6 | | | 1.522 | | | | | | -15.453 | -3.101 |
| | CXCL11 | | 3.553 | 3.386 | | 9.375 | 3.139 | | 9.753 | | 6.140 |
| | CXCL10 | | 3.919 | | 3.879 | 7.710 | 4.549 | | | | 5.462 |
| | CXCL9 | 2.409 | | | 1.830 | 2.815 | 4.725 | | | | 5.910 |
| | IL23A | -1.975 | | 3.145 | | 2.530 | 1.646 | | 2.360 | 1.833 | -1.923 |
| | IL12A | 1.686 | | | | -2.597 | | | 29.177 | -1.547 | |
| | IL12B | | | 2.016 | | 2.451 | | | | | |
| | ARG2 | | | 4.466 | | | | -1.837 | | 1.554 | -3.062 |
| M2a markers | MRC1 | | | | | -2.872 | | | 3.648 | | |
| | CD163 | | | -4.391 | -1.666 | -2.409 | | | | -5.416 | 2.464 |
| | STAB1 | -1.904 | | -6.659 | | 2.371 | | | 4.852 | | 1.863 |
| | CCL18 | 5.163 | 9.085 | -30.216 | 4.011 | 2.501 | | | 5.255 | -3.689 | 12.061 |
| | CD200R1 | | | -2.043 | | | | | | | 2.583 |
| | F13A1 | -2.418 | | -7.536 | | -4.617 | -1.885 | | 2.407 | -5.685 | |
| | IL1RN | -2.423 | -1.606 | | 11.754 | | 2.230 | | 1.513 | -4.357 | 1.628 |
| | ARG1 | -2.744 | -7.532 | | | | | | | | |
| | PDE4DIP | 2.403 | | -6.288 | | -4.034 | -1.792 | | -2.309 | | |
| | Chil4 | | | | | | | | | | |
| | Chil3 | | | | | | | | | | |
| | Retnla | | | | | | | | | | |
| M2b markers | IL10 | | | | | | | | | 2.461 | 1.872 |
| | IL12B | | | 2.016 | | 2.451 | | | | | |
| | IL12A | 1.686 | | | | -2.597 | | | 29.177 | -1.547 | |
| M2c markers | MRC1 | | | | | -2.872 | | | 3.648 | | |
| | ARG1 | -2.744 | -7.532 | | | | | | | | |
| M2d markers | TNF | | | -1.672 | | 1.775 | | | 23.201 | -3.344 | |
| | IL12A | 1.686 | | | | -2.597 | | | 29.177 | -1.547 | |
| | IL12B | | | 2.016 | | 2.451 | | | | | |
| M4 markers | MMP7 | 4.121 | | 72.619 | 5.347 | | 2.352 | | 63.662 | | -5.607 |
| | MRC1 | | | | | -2.872 | | | 3.648 | | |
| | S100A8 | | | 3.706 | | 2.724 | -1.747 | | | -8.158 | |
| Mox markers | HMOX1 | 1.700 | | -3.463 | | | 1.540 | | -3.461 | -2.316 | 2.572 |
| | NFE2L2 | -3.297 | | | | | | -1.651 | -2.763 | | |
| | TXNRD1 | | 4.124 | | 1.866 | | | | | | |
| | SRXN1 | | 4.439 | 1.917 | | 1.696 | | 1.755 | | | |
| M(hb) markers | CD163 | | | -4.391 | -1.666 | -2.409 | | | | -5.416 | 2.464 |
| | MRC1 | | | | | -2.872 | | | 3.648 | | |
| Mhem markers | CD163 | | | -4.391 | -1.666 | -2.409 | | | | -5.416 | 2.464 |
| HA-mac markers | CD163 | | | -4.391 | -1.666 | -2.409 | | | | -5.416 | 2.464 |
| | HLA-DRB1 | | | -3.608 | | | | | 4.419 | -2.609 | 2.295 |
| | HLA-DRA | | | -5.265 | | | 1.888 | | 3.988 | -2.424 | 2.251 |
| | Up | 6/31 | 5/31 | 8/31 | 6/31 | 11/31 | 8/31 | 1/31 | 12/31 | 3/31 | 12/31 |
| | Down | 6/31 | 2/31 | 10/31 | 1/31 | 5/31 | 3/31 | 3/31 | 3/31 | 12/31 | 4/31 |

## Slide 14
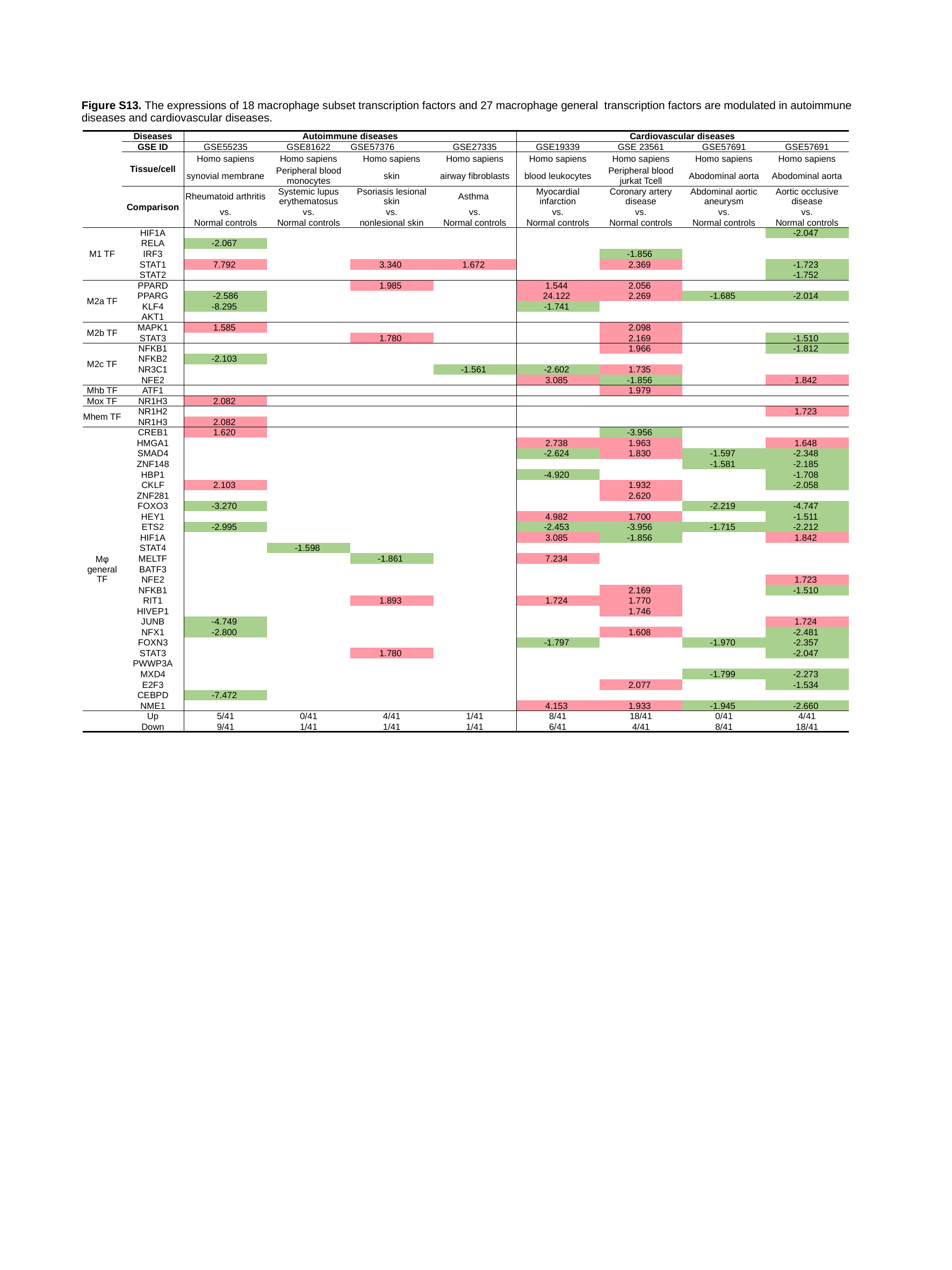

Figure S13. The expressions of 18 macrophage subset transcription factors and 27 macrophage general transcription factors are modulated in autoimmune diseases and cardiovascular diseases.
| | Diseases | Autoimmune diseases | | | | Cardiovascular diseases | | | |
| --- | --- | --- | --- | --- | --- | --- | --- | --- | --- |
| | GSE ID | GSE55235 | GSE81622 | GSE57376 | GSE27335 | GSE19339 | GSE 23561 | GSE57691 | GSE57691 |
| | Tissue/cell | Homo sapiens | Homo sapiens | Homo sapiens | Homo sapiens | Homo sapiens | Homo sapiens | Homo sapiens | Homo sapiens |
| | | synovial membrane | Peripheral blood monocytes | skin | airway fibroblasts | blood leukocytes | Peripheral blood jurkat Tcell | Abodominal aorta | Abodominal aorta |
| | Comparison | Rheumatoid arthritis | Systemic lupus erythematosus | Psoriasis lesional skin | Asthma | Myocardial infarction | Coronary artery disease | Abdominal aortic aneurysm | Aortic occlusive disease |
| | | vs. | vs. | vs. | vs. | vs. | vs. | vs. | vs. |
| | | Normal controls | Normal controls | nonlesional skin | Normal controls | Normal controls | Normal controls | Normal controls | Normal controls |
| M1 TF | HIF1A | | | | | | | | -2.047 |
| | RELA | -2.067 | | | | | | | |
| | IRF3 | | | | | | -1.856 | | |
| | STAT1 | 7.792 | | 3.340 | 1.672 | | 2.369 | | -1.723 |
| | STAT2 | | | | | | | | -1.752 |
| M2a TF | PPARD | | | 1.985 | | 1.544 | 2.056 | | |
| | PPARG | -2.586 | | | | 24.122 | 2.269 | -1.685 | -2.014 |
| | KLF4 | -8.295 | | | | -1.741 | | | |
| | AKT1 | | | | | | | | |
| M2b TF | MAPK1 | 1.585 | | | | | 2.098 | | |
| | STAT3 | | | 1.780 | | | 2.169 | | -1.510 |
| M2c TF | NFKB1 | | | | | | 1.966 | | -1.812 |
| | NFKB2 | -2.103 | | | | | | | |
| | NR3C1 | | | | -1.561 | -2.602 | 1.735 | | |
| | NFE2 | | | | | 3.085 | -1.856 | | 1.842 |
| Mhb TF | ATF1 | | | | | | 1.979 | | |
| Mox TF | NR1H3 | 2.082 | | | | | | | |
| Mhem TF | NR1H2 | | | | | | | | 1.723 |
| | NR1H3 | 2.082 | | | | | | | |
| Mφ general TF | CREB1 | 1.620 | | | | | -3.956 | | |
| | HMGA1 | | | | | 2.738 | 1.963 | | 1.648 |
| | SMAD4 | | | | | -2.624 | 1.830 | -1.597 | -2.348 |
| | ZNF148 | | | | | | | -1.581 | -2.185 |
| | HBP1 | | | | | -4.920 | | | -1.708 |
| | CKLF | 2.103 | | | | | 1.932 | | -2.058 |
| | ZNF281 | | | | | | 2.620 | | |
| | FOXO3 | -3.270 | | | | | | -2.219 | -4.747 |
| | HEY1 | | | | | 4.982 | 1.700 | | -1.511 |
| | ETS2 | -2.995 | | | | -2.453 | -3.956 | -1.715 | -2.212 |
| | HIF1A | | | | | 3.085 | -1.856 | | 1.842 |
| | STAT4 | | -1.598 | | | | | | |
| | MELTF | | | -1.861 | | 7.234 | | | |
| | BATF3 | | | | | | | | |
| | NFE2 | | | | | | | | 1.723 |
| | NFKB1 | | | | | | 2.169 | | -1.510 |
| | RIT1 | | | 1.893 | | 1.724 | 1.770 | | |
| | HIVEP1 | | | | | | 1.746 | | |
| | JUNB | -4.749 | | | | | | | 1.724 |
| | NFX1 | -2.800 | | | | | 1.608 | | -2.481 |
| | FOXN3 | | | | | -1.797 | | -1.970 | -2.357 |
| | STAT3 | | | 1.780 | | | | | -2.047 |
| | PWWP3A | | | | | | | | |
| | MXD4 | | | | | | | -1.799 | -2.273 |
| | E2F3 | | | | | | 2.077 | | -1.534 |
| | CEBPD | -7.472 | | | | | | | |
| | NME1 | | | | | 4.153 | 1.933 | -1.945 | -2.660 |
| | Up | 5/41 | 0/41 | 4/41 | 1/41 | 8/41 | 18/41 | 0/41 | 4/41 |
| | Down | 9/41 | 1/41 | 1/41 | 1/41 | 6/41 | 4/41 | 8/41 | 18/41 |

## Slide 15
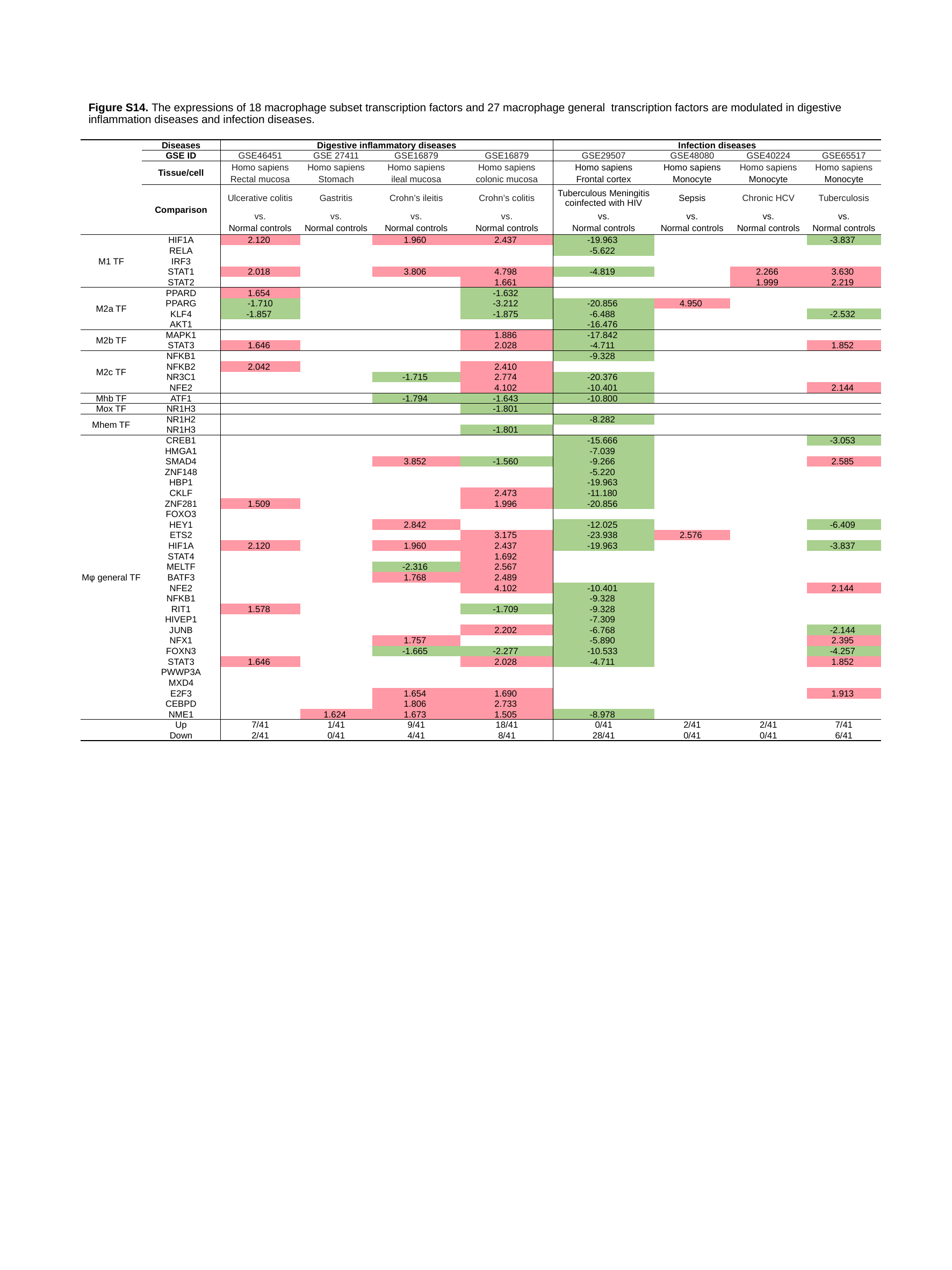

Figure S14. The expressions of 18 macrophage subset transcription factors and 27 macrophage general transcription factors are modulated in digestive inflammation diseases and infection diseases.
| | Diseases | Digestive inflammatory diseases | | | | Infection diseases | | | |
| --- | --- | --- | --- | --- | --- | --- | --- | --- | --- |
| | GSE ID | GSE46451 | GSE 27411 | GSE16879 | GSE16879 | GSE29507 | GSE48080 | GSE40224 | GSE65517 |
| | Tissue/cell | Homo sapiens | Homo sapiens | Homo sapiens | Homo sapiens | Homo sapiens | Homo sapiens | Homo sapiens | Homo sapiens |
| | | Rectal mucosa | Stomach | ileal mucosa | colonic mucosa | Frontal cortex | Monocyte | Monocyte | Monocyte |
| | Comparison | Ulcerative colitis | Gastritis | Crohn’s ileitis | Crohn’s colitis | Tuberculous Meningitis coinfected with HIV | Sepsis | Chronic HCV | Tuberculosis |
| | | vs. | vs. | vs. | vs. | vs. | vs. | vs. | vs. |
| | | Normal controls | Normal controls | Normal controls | Normal controls | Normal controls | Normal controls | Normal controls | Normal controls |
| M1 TF | HIF1A | 2.120 | | 1.960 | 2.437 | -19.963 | | | -3.837 |
| | RELA | | | | | -5.622 | | | |
| | IRF3 | | | | | | | | |
| | STAT1 | 2.018 | | 3.806 | 4.798 | -4.819 | | 2.266 | 3.630 |
| | STAT2 | | | | 1.661 | | | 1.999 | 2.219 |
| M2a TF | PPARD | 1.654 | | | -1.632 | | | | |
| | PPARG | -1.710 | | | -3.212 | -20.856 | 4.950 | | |
| | KLF4 | -1.857 | | | -1.875 | -6.488 | | | -2.532 |
| | AKT1 | | | | | -16.476 | | | |
| M2b TF | MAPK1 | | | | 1.886 | -17.842 | | | |
| | STAT3 | 1.646 | | | 2.028 | -4.711 | | | 1.852 |
| M2c TF | NFKB1 | | | | | -9.328 | | | |
| | NFKB2 | 2.042 | | | 2.410 | | | | |
| | NR3C1 | | | -1.715 | 2.774 | -20.376 | | | |
| | NFE2 | | | | 4.102 | -10.401 | | | 2.144 |
| Mhb TF | ATF1 | | | -1.794 | -1.643 | -10.800 | | | |
| Mox TF | NR1H3 | | | | -1.801 | | | | |
| Mhem TF | NR1H2 | | | | | -8.282 | | | |
| | NR1H3 | | | | -1.801 | | | | |
| Mφ general TF | CREB1 | | | | | -15.666 | | | -3.053 |
| | HMGA1 | | | | | -7.039 | | | |
| | SMAD4 | | | 3.852 | -1.560 | -9.266 | | | 2.585 |
| | ZNF148 | | | | | -5.220 | | | |
| | HBP1 | | | | | -19.963 | | | |
| | CKLF | | | | 2.473 | -11.180 | | | |
| | ZNF281 | 1.509 | | | 1.996 | -20.856 | | | |
| | FOXO3 | | | | | | | | |
| | HEY1 | | | 2.842 | | -12.025 | | | -6.409 |
| | ETS2 | | | | 3.175 | -23.938 | 2.576 | | |
| | HIF1A | 2.120 | | 1.960 | 2.437 | -19.963 | | | -3.837 |
| | STAT4 | | | | 1.692 | | | | |
| | MELTF | | | -2.316 | 2.567 | | | | |
| | BATF3 | | | 1.768 | 2.489 | | | | |
| | NFE2 | | | | 4.102 | -10.401 | | | 2.144 |
| | NFKB1 | | | | | -9.328 | | | |
| | RIT1 | 1.578 | | | -1.709 | -9.328 | | | |
| | HIVEP1 | | | | | -7.309 | | | |
| | JUNB | | | | 2.202 | -6.768 | | | -2.144 |
| | NFX1 | | | 1.757 | | -5.890 | | | 2.395 |
| | FOXN3 | | | -1.665 | -2.277 | -10.533 | | | -4.257 |
| | STAT3 | 1.646 | | | 2.028 | -4.711 | | | 1.852 |
| | PWWP3A | | | | | | | | |
| | MXD4 | | | | | | | | |
| | E2F3 | | | 1.654 | 1.690 | | | | 1.913 |
| | CEBPD | | | 1.806 | 2.733 | | | | |
| | NME1 | | 1.624 | 1.673 | 1.505 | -8.978 | | | |
| | Up | 7/41 | 1/41 | 9/41 | 18/41 | 0/41 | 2/41 | 2/41 | 7/41 |
| | Down | 2/41 | 0/41 | 4/41 | 8/41 | 28/41 | 0/41 | 0/41 | 6/41 |

## Slide 16
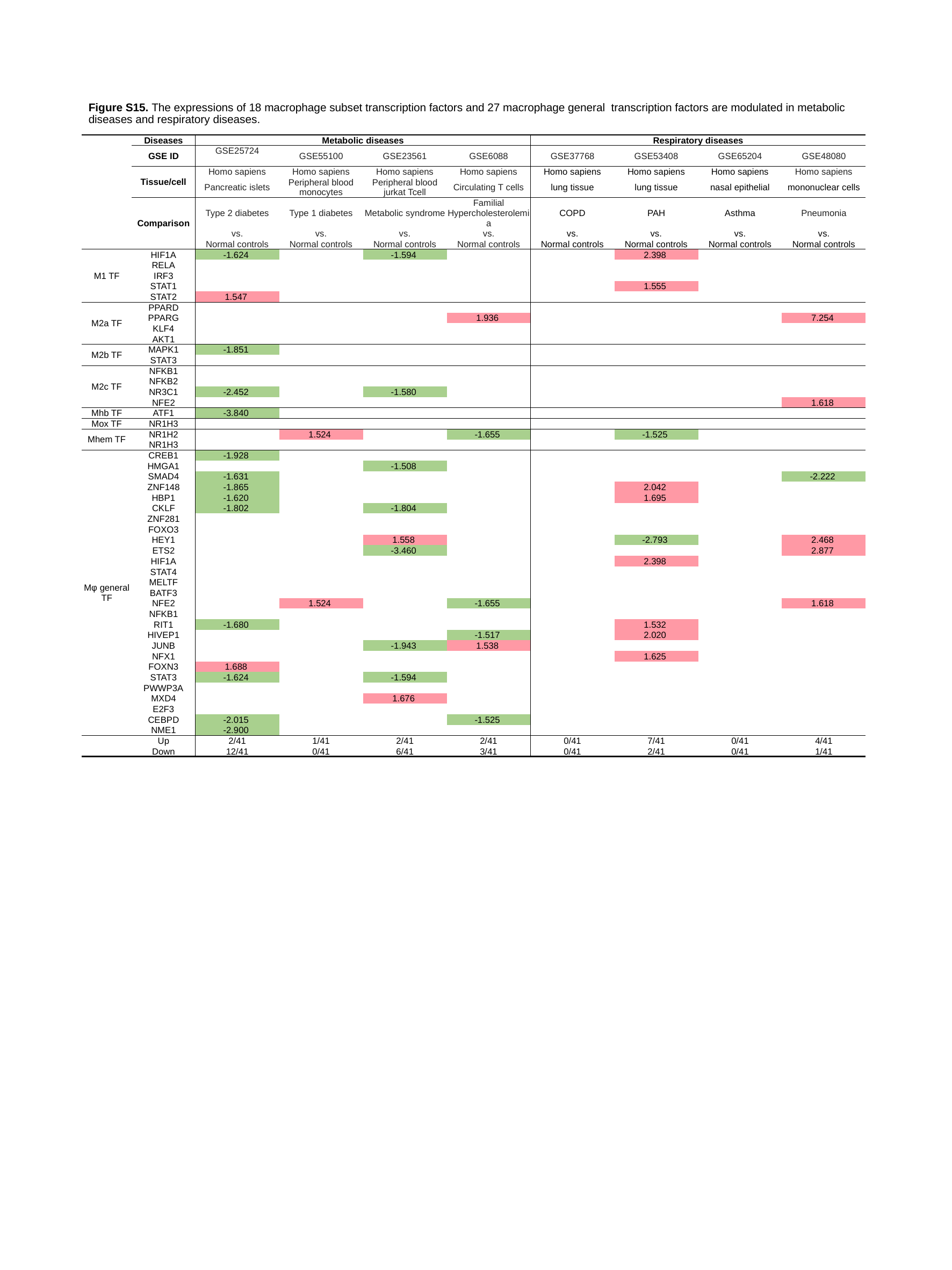

Figure S15. The expressions of 18 macrophage subset transcription factors and 27 macrophage general transcription factors are modulated in metabolic diseases and respiratory diseases.
| | Diseases | Metabolic diseases | | | | Respiratory diseases | | | |
| --- | --- | --- | --- | --- | --- | --- | --- | --- | --- |
| | GSE ID | GSE25724 | GSE55100 | GSE23561 | GSE6088 | GSE37768 | GSE53408 | GSE65204 | GSE48080 |
| | Tissue/cell | Homo sapiens | Homo sapiens | Homo sapiens | Homo sapiens | Homo sapiens | Homo sapiens | Homo sapiens | Homo sapiens |
| | | Pancreatic islets | Peripheral blood monocytes | Peripheral blood jurkat Tcell | Circulating T cells | lung tissue | lung tissue | nasal epithelial | mononuclear cells |
| | Comparison | Type 2 diabetes | Type 1 diabetes | Metabolic syndrome | Familial Hypercholesterolemia | COPD | PAH | Asthma | Pneumonia |
| | | vs. | vs. | vs. | vs. | vs. | vs. | vs. | vs. |
| | | Normal controls | Normal controls | Normal controls | Normal controls | Normal controls | Normal controls | Normal controls | Normal controls |
| M1 TF | HIF1A | -1.624 | | -1.594 | | | 2.398 | | |
| | RELA | | | | | | | | |
| | IRF3 | | | | | | | | |
| | STAT1 | | | | | | 1.555 | | |
| | STAT2 | 1.547 | | | | | | | |
| M2a TF | PPARD | | | | | | | | |
| | PPARG | | | | 1.936 | | | | 7.254 |
| | KLF4 | | | | | | | | |
| | AKT1 | | | | | | | | |
| M2b TF | MAPK1 | -1.851 | | | | | | | |
| | STAT3 | | | | | | | | |
| M2c TF | NFKB1 | | | | | | | | |
| | NFKB2 | | | | | | | | |
| | NR3C1 | -2.452 | | -1.580 | | | | | |
| | NFE2 | | | | | | | | 1.618 |
| Mhb TF | ATF1 | -3.840 | | | | | | | |
| Mox TF | NR1H3 | | | | | | | | |
| Mhem TF | NR1H2 | | 1.524 | | -1.655 | | -1.525 | | |
| | NR1H3 | | | | | | | | |
| Mφ general TF | CREB1 | -1.928 | | | | | | | |
| | HMGA1 | | | -1.508 | | | | | |
| | SMAD4 | -1.631 | | | | | | | -2.222 |
| | ZNF148 | -1.865 | | | | | 2.042 | | |
| | HBP1 | -1.620 | | | | | 1.695 | | |
| | CKLF | -1.802 | | -1.804 | | | | | |
| | ZNF281 | | | | | | | | |
| | FOXO3 | | | | | | | | |
| | HEY1 | | | 1.558 | | | -2.793 | | 2.468 |
| | ETS2 | | | -3.460 | | | | | 2.877 |
| | HIF1A | | | | | | 2.398 | | |
| | STAT4 | | | | | | | | |
| | MELTF | | | | | | | | |
| | BATF3 | | | | | | | | |
| | NFE2 | | 1.524 | | -1.655 | | | | 1.618 |
| | NFKB1 | | | | | | | | |
| | RIT1 | -1.680 | | | | | 1.532 | | |
| | HIVEP1 | | | | -1.517 | | 2.020 | | |
| | JUNB | | | -1.943 | 1.538 | | | | |
| | NFX1 | | | | | | 1.625 | | |
| | FOXN3 | 1.688 | | | | | | | |
| | STAT3 | -1.624 | | -1.594 | | | | | |
| | PWWP3A | | | | | | | | |
| | MXD4 | | | 1.676 | | | | | |
| | E2F3 | | | | | | | | |
| | CEBPD | -2.015 | | | -1.525 | | | | |
| | NME1 | -2.900 | | | | | | | |
| | Up | 2/41 | 1/41 | 2/41 | 2/41 | 0/41 | 7/41 | 0/41 | 4/41 |
| | Down | 12/41 | 0/41 | 6/41 | 3/41 | 0/41 | 2/41 | 0/41 | 1/41 |

## Slide 17
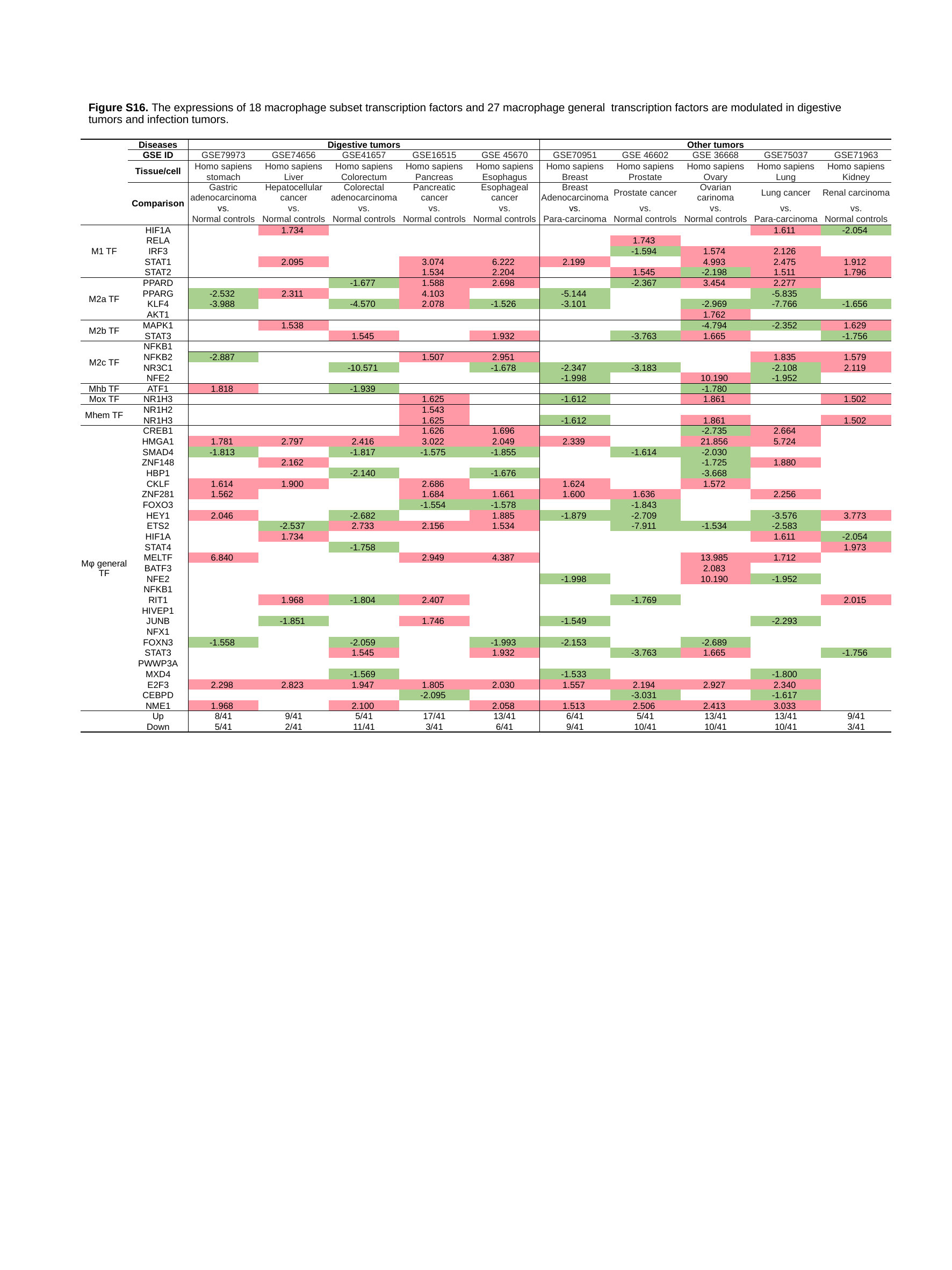

Figure S16. The expressions of 18 macrophage subset transcription factors and 27 macrophage general transcription factors are modulated in digestive tumors and infection tumors.
| | Diseases | Digestive tumors | | | | | Other tumors | | | | |
| --- | --- | --- | --- | --- | --- | --- | --- | --- | --- | --- | --- |
| | GSE ID | GSE79973 | GSE74656 | GSE41657 | GSE16515 | GSE 45670 | GSE70951 | GSE 46602 | GSE 36668 | GSE75037 | GSE71963 |
| | Tissue/cell | Homo sapiens | Homo sapiens | Homo sapiens | Homo sapiens | Homo sapiens | Homo sapiens | Homo sapiens | Homo sapiens | Homo sapiens | Homo sapiens |
| | | stomach | Liver | Colorectum | Pancreas | Esophagus | Breast | Prostate | Ovary | Lung | Kidney |
| | Comparison | Gastric adenocarcinoma | Hepatocellular cancer | Colorectal adenocarcinoma | Pancreatic cancer | Esophageal cancer | Breast Adenocarcinoma | Prostate cancer | Ovarian carinoma | Lung cancer | Renal carcinoma |
| | | vs. | vs. | vs. | vs. | vs. | vs. | vs. | vs. | vs. | vs. |
| | | Normal controls | Normal controls | Normal controls | Normal controls | Normal controls | Para-carcinoma | Normal controls | Normal controls | Para-carcinoma | Normal controls |
| M1 TF | HIF1A | | 1.734 | | | | | | | 1.611 | -2.054 |
| | RELA | | | | | | | 1.743 | | | |
| | IRF3 | | | | | | | -1.594 | 1.574 | 2.126 | |
| | STAT1 | | 2.095 | | 3.074 | 6.222 | 2.199 | | 4.993 | 2.475 | 1.912 |
| | STAT2 | | | | 1.534 | 2.204 | | 1.545 | -2.198 | 1.511 | 1.796 |
| M2a TF | PPARD | | | -1.677 | 1.588 | 2.698 | | -2.367 | 3.454 | 2.277 | |
| | PPARG | -2.532 | 2.311 | | 4.103 | | -5.144 | | | -5.835 | |
| | KLF4 | -3.988 | | -4.570 | 2.078 | -1.526 | -3.101 | | -2.969 | -7.766 | -1.656 |
| | AKT1 | | | | | | | | 1.762 | | |
| M2b TF | MAPK1 | | 1.538 | | | | | | -4.794 | -2.352 | 1.629 |
| | STAT3 | | | 1.545 | | 1.932 | | -3.763 | 1.665 | | -1.756 |
| M2c TF | NFKB1 | | | | | | | | | | |
| | NFKB2 | -2.887 | | | 1.507 | 2.951 | | | | 1.835 | 1.579 |
| | NR3C1 | | | -10.571 | | -1.678 | -2.347 | -3.183 | | -2.108 | 2.119 |
| | NFE2 | | | | | | -1.998 | | 10.190 | -1.952 | |
| Mhb TF | ATF1 | 1.818 | | -1.939 | | | | | -1.780 | | |
| Mox TF | NR1H3 | | | | 1.625 | | -1.612 | | 1.861 | | 1.502 |
| Mhem TF | NR1H2 | | | | 1.543 | | | | | | |
| | NR1H3 | | | | 1.625 | | -1.612 | | 1.861 | | 1.502 |
| Mφ general TF | CREB1 | | | | 1.626 | 1.696 | | | -2.735 | 2.664 | |
| | HMGA1 | 1.781 | 2.797 | 2.416 | 3.022 | 2.049 | 2.339 | | 21.856 | 5.724 | |
| | SMAD4 | -1.813 | | -1.817 | -1.575 | -1.855 | | -1.614 | -2.030 | | |
| | ZNF148 | | 2.162 | | | | | | -1.725 | 1.880 | |
| | HBP1 | | | -2.140 | | -1.676 | | | -3.668 | | |
| | CKLF | 1.614 | 1.900 | | 2.686 | | 1.624 | | 1.572 | | |
| | ZNF281 | 1.562 | | | 1.684 | 1.661 | 1.600 | 1.636 | | 2.256 | |
| | FOXO3 | | | | -1.554 | -1.578 | | -1.843 | | | |
| | HEY1 | 2.046 | | -2.682 | | 1.885 | -1.879 | -2.709 | | -3.576 | 3.773 |
| | ETS2 | | -2.537 | 2.733 | 2.156 | 1.534 | | -7.911 | -1.534 | -2.583 | |
| | HIF1A | | 1.734 | | | | | | | 1.611 | -2.054 |
| | STAT4 | | | -1.758 | | | | | | | 1.973 |
| | MELTF | 6.840 | | | 2.949 | 4.387 | | | 13.985 | 1.712 | |
| | BATF3 | | | | | | | | 2.083 | | |
| | NFE2 | | | | | | -1.998 | | 10.190 | -1.952 | |
| | NFKB1 | | | | | | | | | | |
| | RIT1 | | 1.968 | -1.804 | 2.407 | | | -1.769 | | | 2.015 |
| | HIVEP1 | | | | | | | | | | |
| | JUNB | | -1.851 | | 1.746 | | -1.549 | | | -2.293 | |
| | NFX1 | | | | | | | | | | |
| | FOXN3 | -1.558 | | -2.059 | | -1.993 | -2.153 | | -2.689 | | |
| | STAT3 | | | 1.545 | | 1.932 | | -3.763 | 1.665 | | -1.756 |
| | PWWP3A | | | | | | | | | | |
| | MXD4 | | | -1.569 | | | -1.533 | | | -1.800 | |
| | E2F3 | 2.298 | 2.823 | 1.947 | 1.805 | 2.030 | 1.557 | 2.194 | 2.927 | 2.340 | |
| | CEBPD | | | | -2.095 | | | -3.031 | | -1.617 | |
| | NME1 | 1.968 | | 2.100 | | 2.058 | 1.513 | 2.506 | 2.413 | 3.033 | |
| | Up | 8/41 | 9/41 | 5/41 | 17/41 | 13/41 | 6/41 | 5/41 | 13/41 | 13/41 | 9/41 |
| | Down | 5/41 | 2/41 | 11/41 | 3/41 | 6/41 | 9/41 | 10/41 | 10/41 | 10/41 | 3/41 |
